# Using BONCAT to dissect the proteome of *S. aureus* persisters

**DOI:** 10.1128/msphere.00431-25

**Published:** 2025-09-08

**Authors:** Eva D. C. George Matlalcuatzi, Thomas Bakkum, Pooja S. Thomas, Stephan Hacker, Bogdan I. Florea, Bastienne Vriesendorp, Daniel E. Rozen, Sander I. van Kasteren

**Affiliations:** 1 Leiden Institute of Chemistry and The Institute of Chemical Immunology, Leiden University 4496, Leiden, the Netherlands; 2 Institute of Biology Leiden, Leiden University 4496, Leiden, the Netherlands; University of Galway, Galway, Ireland

**Keywords:** BONCAT, bacterial persisters, proteomics

## Abstract

Bacterial persisters are a subpopulation of cells that exhibit a transient non-susceptible phenotype in the presence of bactericidal antibiotic concentrations. This phenotype can lead to the survival and regrowth of bacteria after treatment, resulting in relapse of infections. It is also a contributing factor to antibacterial resistance. Multiple processes are believed to cause persister formation; however, identifying the proteins expressed during the induction of persistence is challenging because the persister state is rare, transient, and does not result in genetic changes. In this study, we used Bio-Orthogonal Non-Canonical Amino Acid Tagging (BONCAT) to label and retrieve the proteome expressed during persistence and recovery for two strains of *Staphylococcus aureus* exposed to β-lactam and fluoroquinolone antibiotics. After incubating antibiotic-exposed bacteria with the methionine ortholog L-azidohomoalanine to label the proteins of persister cells, we retrieved labeled proteins using click chemistry-pulldown methodology. Analysis of the retrieved proteome of persisters with Label-Free Quantification-Liquid chromatography mass spectrometry (LFQ-LCMS)-based proteomics revealed widespread changes in translation. Our analysis uncovered previously identified persister genes, including, for example, *relA/spot*-system, changes in purine and amino acid metabolism, the upregulation and downregulation of transcription factors, and changes to influx and efflux pumps, thus validating our methodology. In addition, we also identified numerous novel persister-associated proteins. Few changes were conserved across the two strains and both antibiotics. Instead, results suggest that the mechanisms of persister formation vary across genotypes and the drugs to which strains are exposed. These findings provide evidence that the entry into persistence is an active process that dramatically alters the translational behavior of cells and suggest that downregulation of metabolism, by diverse but functionally similar processes, in persister cells enables cells to survive antibiotic pressure.

**IMPORTANCE:**

In this study, we have applied a technique called “Bioorthogonal Non-Canonical Amino Acid-Tagging,” or BONCAT, to identify which proteins are expressed when bacteria are in the persister state. Our work makes novel contributions to our understanding of persister cells, a bacterial sub-population that gives rise to recurrent infections, and establishes BONCAT as a valuable tool to study phenotypic heterogeneity in bacterial populations.

## INTRODUCTION

Persisters are a subpopulation of bacteria that adopt a dormant, slow/no-growth state after exposure to lethal antibiotic concentrations (1). Because these cells can survive antibiotic challenge and then resume growth after antibiotic removal, they are a central cause of treatment failure and relapse. They are also more likely to evolve antibiotic resistance (2). Persisters are ubiquitous in microbes and have been detected in fungi (3) as well as gram-positive and gram-negative bacteria, including major pathogens such as *Mycobacterium tuberculosis (4*), *Escherichia coli* (5), *Pseudomonas aeruginosa (6*), *Salmonella typhimurium (7*), and *Staphylococcus aureus (8*). However, the mechanisms underlying this phenotype remain uncertain.

Understanding how bacteria enter, maintain, and exit persistence is challenging because persister cells comprise only a small fraction of an antibiotic-treated population. Their rarity makes it difficult to specifically isolate this minority for mechanistic study (9–12). A second complicating factor is that the persister phenotype is transient, with bacteria reverting to antibiotic susceptibility after removal of the antibiotic challenge. Different approaches to study persisters have been developed, including proteomic and metabolomic analysis of flow cytometry-sorted cells (13–15), imaging persisters *in situ* (16), separating persisters using microfluidics (17), or analyzing the phenotypes of targeted knock-outs of putative “persister genes” (18, 19). These techniques are, however, all complicated by the presence of dead/dying cells during the experiments, the presence of highly dormant “deep” persisters, as well as cells reverting back to their non-persister form during isolation and analysis. These limitations make it difficult to distinguish the signal of true persister proteins and likely lead to an underestimation of changes in persister protein expression.

Our aim in this paper is to develop a novel method to analyze the proteome of persister cells in the gram-positive pathogen *S. aureus,* the causative agent for a variety of chronic and relapsing infections (8, 20–22). Persistence has been well studied in *S. aureus* since this phenomenon was discovered in this species in the 1940s and is the subject of numerous reviews (20–22). Results from different studies using a broad array of methods have uncovered common molecular changes arising in persister populations, including changes to stress response proteins, the stringent response via RelA/SpoT (23, 24), cell wall stress (25), and metabolism of purine (26) and amino acids (13, 27) and in toxin-antitoxin systems. Other studies have reported changes in ABC transporter efflux pumps, cell wall biosynthesis, protein synthesis, and other anabolic processes, virulence, cell-to-cell communication, and quorum sensing (28). Although there is some overlap between studies, significant differences likely reflect the different approaches used to isolate and analyze persister cells, as well as the fact that the mechanisms of persistence vary as a function of genotype and antibiotics studied. Accordingly, our understanding of the persister proteome remains limited.

The central problem of studying persisters using proteomics is that it is difficult to separate the proteome of persisters from the proteome of the remainder of the dead/dying population, especially if translational activity in persister cells is low. Brul and co-workers recently used a combination of a metabolically conditional dye (carboxyfluoresceindiacetate succinimidyl ester) with a viability dye (propidium iodide) to separate persisters from non-persisters and dying cells and isolate these cells by flow cytometry (29–31). This method allowed the isolation of those cells, likely the persister fraction, that were impermeable to propidium iodide, yet still had the esterase activity to activate CFSE (and that did not divide) and analyze these by proteomics after flow cytometry. However, methods that can easily achieve the same outcome from unsorted bulk populations, or from antibiotics that do not result in increased cell wall permeability, have not been reported. To address this problem, we hypothesized that bioorthogonal, or “click,” chemistry would allow deeper protein coverage of rare persister cells because it enables retrieval and isolation of the (very rare) proteome during the various stages of persistence, followed by characterization of only the proteins that were expressed in persister cells. Accordingly, this approach should reduce background “noise” in the measured proteome and focus attention on proteins that are more likely to be causally important in persisters.

**Fig 1:**
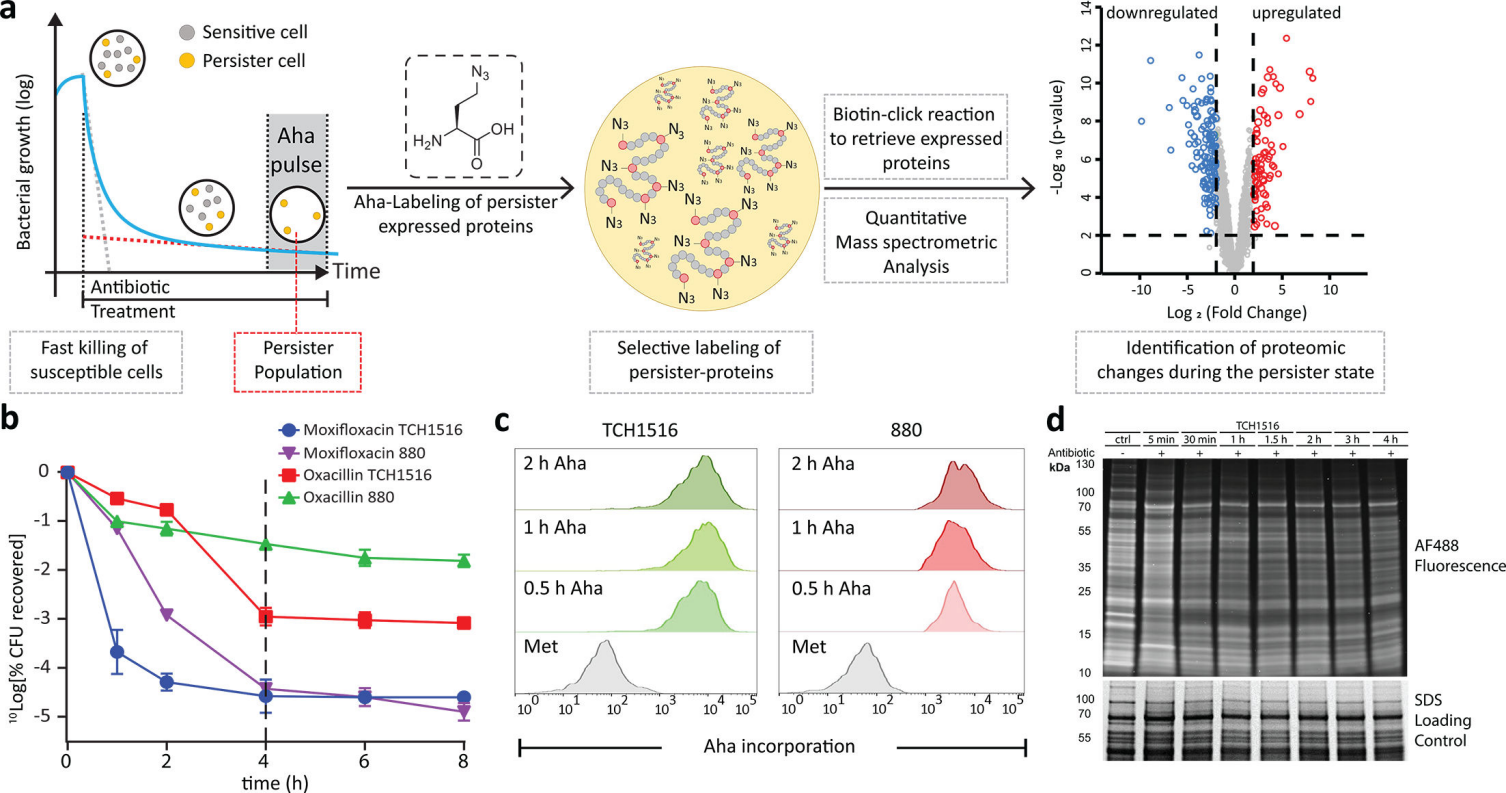
Overview of BONCAT labeling of persister cells. (**a**) Overview of the workflow: first, persisters are induced by the addition of 50× MIC of an antibiotic until the time-kill curve flattens. Then an azide-modified amino acid is added to label only those rare proteins expressed by the translationally active cells at this time. These can be retrieved by a click reaction with biotin, followed by streptavidin retrieval, trypsinization, and mass spectrometry-based quantification and identification. (**b**) Time-kill curves for the two strains (880 and TCH1516) and antibiotics (oxacillin and moxifloxacin) used. The Y-axis shows the number of CFU/mL retrieved from the treated culture. *N* = 3 and error bars indicate s.e.m. (**c**) Flow cytometry histogram after fluorescence of azide-labeled TCH1516 and 880 with Alexa-488 alkyne under log growth conditions. Representative image of *N* = 3, 100,000 events per condition. (**d**) Fluorescent SDS-PAGE analysis of cell lysate of TCH1516 treated with moxifloxacin at 50× MIC, which was clicked with Alexa-488-alkyne, showing incorporation of azides even after the addition of antibiotic and during persister induction.

Click reactions are a family of high-yielding, fast, and selective chemical ligation reactions that can be performed with a high degree of selectivity in biological environments (32). They have been used extensively to, for example, image the location of specific lipids in cells (33, 34), look at surface regulation of carbohydrates (35), quantify DNA synthesis in cells (36–38), and study nutrient channel activities (39, 40). This approach has also been used to label the expressed proteome across a given time window. This approach, called Bio-Orthogonal Non-Canonical Amino Acid Tagging (BONCAT) (41, 42), makes use of the fact that in many species, the methionine tRNA/tRNA-synthase pair can accept and incorporate unnatural amino acids with click-reactive chemical groups (43, 44). The two best mimics of methionine are azidohomoalanine (Aha, Fig. 1a) and homopropargylglycine (Hpg), which can both be ligated using copper-catalyzed [3 + 2]-cycloaddition reactions, which is a very low-background click reaction (42, 43, 45–47). When cells are pulsed with these amino acids, they are incorporated into the proteome of translationally active cells only during the pulse period, thereby enriching the proteome for the specific set of proteins expressed during a short time window. Here, we report using BONCAT-labeling (41, 42, 48, 49) to identify expressed proteins in persister cells that form during and after the exposure of two different *S. aureus* strains to oxacillin or moxifloxacin.

By characterizing persistence and recovery in two different strains with two antibiotics that vary in their mechanism of action, it was possible to identify strain and antibiotic-specific persistence-related proteins. Furthermore, by comparing the proteins expressed during different times after antibiotic challenge, we could monitor temporal changes in protein expression as bacteria enter and exit the persister phase. Our results uncovered widespread changes to hundreds of proteins during *S. aureus* exposure to two different types of antibiotics. While there were some shared responses to some proteins across strains and antibiotics, most changes were strain or antibiotic specific, consistent with the multifactorial nature of persistence. Despite differences in the specific proteome changes in the two strains, our results suggest that the translation activity of persisters reflects an active defense against antibiotic pressure for survival. They also strongly validate the utility of a BONCAT approach for analyzing the persister proteome.

## RESULTS

### Method optimization

To study the persister proteome, we examined the response of two strains of *S. aureus*, namely the VRSA-strain 880 (50) and the USA300 subspecies TCH1516 (51) to two antibiotics: a β-lactam that targets cell wall biosynthesis (oxacillin, Oxa) (52), and a fluoroquinolone that inhibits DNA-gyrase (moxifloxacin, or moxi) (53). These antibiotics have different modes of action and also result in different frequencies of persister cells. Oxacillin is a lytic antibiotic, facilitating the isolation of intact persisters more easily, while moxifloxacin serves as a rigorous challenge to assess the effectiveness of the method used in this study. Both strains are weakly resistant to methicillin but remain susceptible to the high Oxa concentrations used here, while 880 is also resistant to vancomycin (54, 55). Estimates for the minimum inhibitory concentrations (MIC) of each antibiotic-strain combination were consistent with previously reported values (56, 57). The MIC for TCH1516 was 10 mg/L for oxacillin and 0.08 mg/L for moxifloxacin, while strain 880 had an MIC of 20 mg/L for oxacillin and 2.5 mg/L for moxifloxacin.

To explore the dynamics of antibiotic-mediated killing, and to confirm induction of persisters, we used time-kill curves to measure bacterial CFU over time (58) during exposure to 50× the MIC of each antibiotic (for that particular strain) (Fig. 1b) (59, 60). As anticipated, we observed characteristic biphasic killing for both antibiotics, where an initially rapid drop in CFUs was followed by a second phase where the decline was far slower (61, 62). In accordance with earlier studies, we classified the fraction of surviving cells at 4 h as persisters, which varied both for strain and for each antibiotic (63). The persister fractions of TCH1516 and 880 for oxacillin were 0.1% and 3%, respectively, while the corresponding fractions for moxifloxacin were significantly lower, at 0.002% and 0.003%.

To assess whether bacterial persisters were able to resume growth following antibiotic exposure, we also measured recovery in TCH1516 after the antibiotic stress was removed after 4 hours of treatment. Oxacillin and moxifloxacin were removed via pelleting and washing the bacteria and then resuspending them in fresh medium. The subsequent outgrowth phase was monitored over 2.5 hours by CFU count (Fig. S1). This showed that persister cells that survived 4 hours of antibiotic exposure remained viable and could resume growth once antibiotic stress was removed, albeit with a small lag phase before growth resumed.

Having established the conditions for inducing persisters with moxifloxacin and oxacillin, we next determined whether a BONCAT approach (Fig. 1a) could identify whether these strains were translationally active, and whether the technique was sensitive and selective enough to retrieve and identify the translated proteome from this small minority of cells. To do this, we first established BONCAT treatment conditions by determining whether the bioorthogonal amino acid l-azidohomoalanine (Aha) was incorporated in unchallenged 880 MRSA by incubating cells with 4 mM of this amino acid, in line with the concentration range used to label other species (41, 64–66). To visualize Aha uptake, the Cu-catalyzed Huisgen cyclo-addition click reaction (CCHC) was then performed on fixed and permeabilized cells (67). This reaction leads to the specific ligation of a fluorophore to the azide residues of Aha, with relatively little background, and can thus be used to analyze the uptake of the amino acid on a per cell basis using flow cytometry (68, 69). These experiments showed an approximately 70-fold increase in fluorescence after 0.5 h incubation with Aha (Fig. 1c). To determine whether uptake was associated with incorporation into the proteome (41, 70–72), cells were lysed, subjected to CCHC with an alkyne-Alexa488 fluorophore, and analyzed by fluorescent SDS-PAGE. The presence of a fluorescent band at a given MW would indicate the presence of an Aha-labeled protein at that weight. These gels (Fig. 1d) showed incorporation of the azide into the *S. aureus* proteome, with a decrease in protein expression observed as the bacteria were incubated longer with the antibiotic.

Having confirmed the incorporation of Aha into the nascent MRSA-proteome, we next assessed whether labeling levels were sufficient to also retrieve and analyze the expressed proteome by mass spectrometry. This was first done on non-antibiotic-treated cells. The azides from the incorporated Aha of untreated bacteria were reacted with biotin-PEG-alkyne and retrieved using a neutravidin resin that could selectively pull down the Aha-containing proteins (Fig. 1a) (73). We chose the longer pulse length of 90 minutes to ensure the potential reduction in uptake of Aha in the persister state was negated. Retrieval of the proteins by the above protocol, followed by trypsin digestion and LFQ-LCMS analysis (74–77), resulted in the identification of 1,538 proteins produced by strain TCH1516 and 1,451 proteins by strain 880 during log phase growth (supplementary spreadsheet Tables S6 and S7). A direct comparison of the Aha-labeled proteome with LFQ-LCMS to that of unlabeled *S. aureus* that was not subjected to BONCAT, but directly trypsinized after lysis and analyzed by LFQ-LCMS showed that >90% of the whole proteome was recovered in the BONCAT experiment (Fig. S2), confirming that BONCAT-MS could be used to robustly retrieve the expressed MRSA proteome without bias from the Aha-labeling.

### BONCAT-MS of persister-expressed proteins

**Fig 2:**
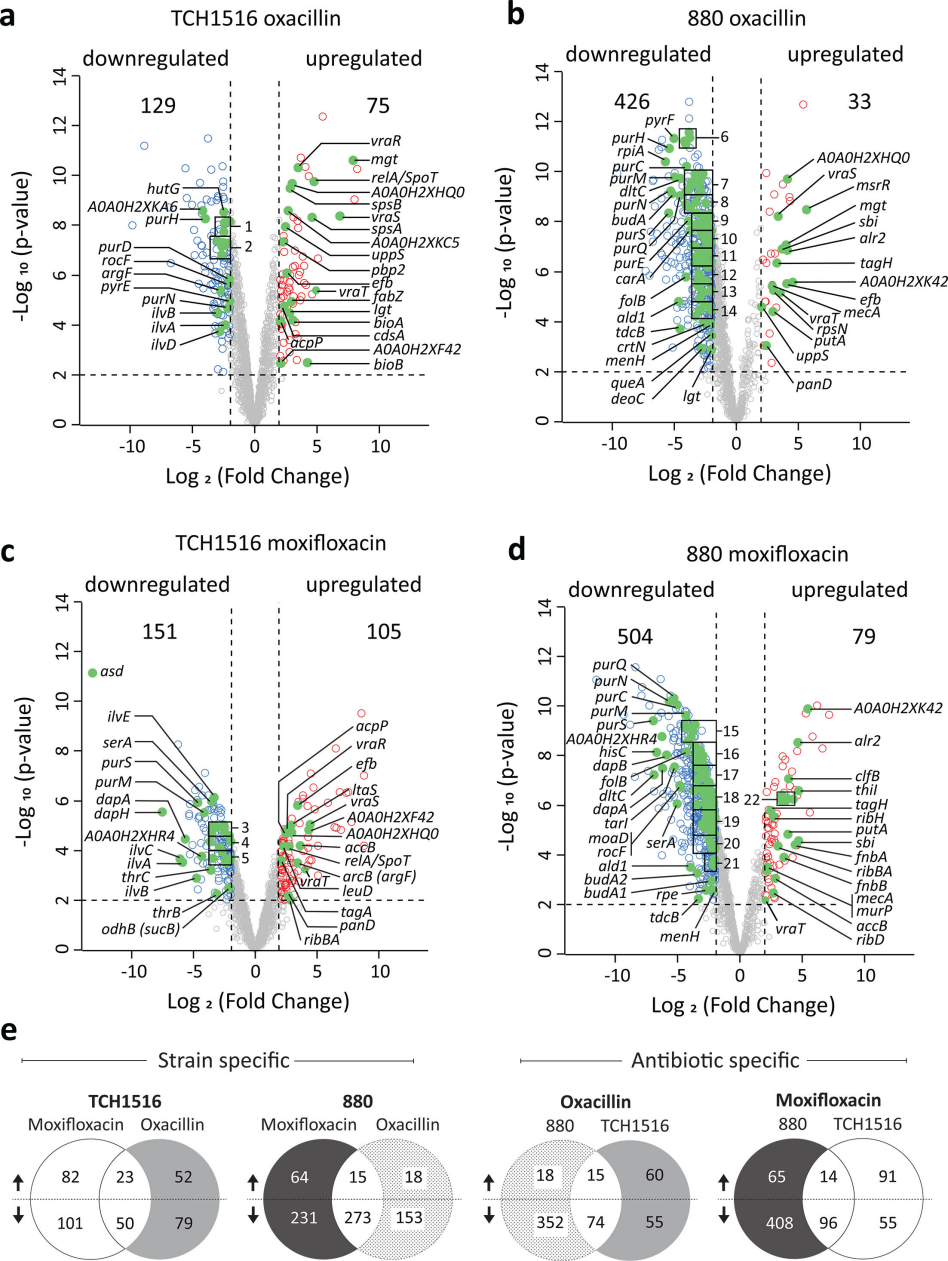
Identified proteins at 4 hours after antibiotic challenge. (**a–d**) show up/downregulated proteins for each antibiotic-strain combination. The list of pathway-identified proteins is found in Tables 1 to 4 and all proteins in Tables S1 to S4. (**e**) Quantification of proteins found shared by a strain in response to both antibiotics, or found across the strains in response to the same antibiotic.

We next applied BONCAT to the isolation and identification of proteins expressed in persisters during antibiotic exposure. Strains TCH1516 and 880 were treated with oxacillin and moxifloxacin for 4 hours as above, followed by the addition of Aha for 1.5 hours. After this time, the cells were lysed, and fluorescent SDS-PAGE was used to check whether detectable levels of Aha-positive proteins were produced during this period (Fig. 1d), consistent with the previously reported translational activity of the persister population (23, 78–81). Having confirmed incorporation, the Aha-treated persisters (4–5.5h in 50× MIC antibiotic) were analyzed by BONCAT-LFQ-LCMS and the expressed proteomes compared to those of the log-phase growing parent strains. Extensive proteome changes were observed (Tables 1 to 4; Tables S1 to S4), with the number and identity of the upregulated or downregulated proteins depending on the strain and antibiotic (Fig. 2a through d). Overall, we observed far more protein downregulation than upregulation. In strain 880, persisters induced during oxacillin challenge upregulated 33 proteins and downregulated 426; when challenged with moxifloxacin, 880 upregulated 79 proteins and downregulated 504. Strain TC1516 showed less downregulation compared to strain 880, with 75 proteins upregulated and 129 downregulated upon oxacillin challenge, and 105 proteins upregulated and 151 downregulated upon moxifloxacin challenge (Fig. 2e). A surprisingly small number of protein changes was shared between all strain/antibiotic combinations, with only *vraT* being upregulated, which is part of the VraTSR three-component sensory regulatory system, in all samples and 22 proteins downregulated across all samples. Even within the strains, or upon challenge with the same antibiotics, the overlap in proteins found was limited (Fig. 2e). These quantitative differences indicate the divergent strain and antibiotic specificities of the persister proteome. They also highlight the extreme sensitivity of the BONCAT approach to detect translational responses in rare cell populations, and thereby, the ability to compare different strain-antibiotic combinations. This is particularly clear in the cells treated with moxifloxacin, where robust proteomic analysis could be obtained even in the presence of >99.997% dead bacteria.

**TABLE 1:** Pathway assigned up/downregulated genes for TCH1516 under oxacillin treatment

Gene name	Pathway	Protein name	log2 Fold change (FC)
Pathway assigned upregulated genes			
*A0A0H2XKC5*	Beta-Lactam resistance	ABC transporter, ATP-binding protein	2.64
*pbp2*	Beta-Lactam resistance	Peptidoglycan glycosyltransferase	2.25
*fabZ*	Biotin metabolism	3-Hydroxyacyl-[acyl-carrier-protein] dehydratase FabZ	2.93
*mgt*	Cell wall biogenesis	Monofunctional glycosyltransferase	7.86
*bioB*	Cofactor biosynthesis	Biotin synthase	4.23
*bioA*	Cofactor biosynthesis	Adenosylmethionine-8-amino-7-oxononanoate aminotransferase	3.06
*cdsA*	Lipid metabolism	Phosphatidate cytidylyltransferase	2.34
*acpP*	Lipid metabolism	Acyl carrier protein (ACP)	2.04
*uppS*	Peptidoglycan biosynthesis	Isoprenyl transferase	2.46
*spsA*	Protein export	Signal peptidase I	4.60
*spsB*	Protein export	Signal peptidase I	2.82
*lgt*	Protein modification	Phosphatidylglycerol—prolipoprotein diacylglyceryl transferase	2.77
*RelA/SpoT*	Purine metabolism; ppGpp biosynthesis	RelA/SpoT domain-containing protein	4.73
*efb*	*Staphylococcus aureus* infection	Fibrinogen-binding protein	2.57
*A0A0H2XHQ0*	Teichoic acid biosynthesis	Putative transcriptional regulator	3.00
*vraS*	Two-component system	Sensor protein VraS	6.81
*vraR*	Two-component system	DNA-binding response regulator	3.48
*A0A0H2XF42*	Two-component system	Cytochrome D ubiquinol oxidase, subunit I	2.06
*vraT*	Two-component system	Cell wall-active antibiotics response LiaF-like C-terminal domain-containing protein	4.90
Pathway assigned downregulated genes			
*A0A0H2XKA6*	Amino acid biosynthesis	Probable succinyl-diaminopimelate desuccinylase	−4.21
*ilvB*	Amino acid biosynthesis	Acetolactate synthase	−3.00
*ilvD*	Amino acid biosynthesis	Dihydroxy-acid dehydratase (DAD)	−2.82
*argF*	Amino acid biosynthesis	Ornithine carbamoyltransferase (OTCase)	−2.76
*ilvA*	Amino acid biosynthesis	L-threonine dehydratase biosynthetic IlvA	−2.41
*ald1*	Amino acid degradation	Alanine dehydrogenase 1	−2.97
*tdcB*	Amino acid degradation	L-threonine dehydratase catabolic TdcB	−2.65
*hutG*	Amino acid degradation	Formimidoylglutamase	−2.49
*rpiA*	Carbohydrate degradation	Ribose-5-phosphate isomerase A	−2.38
*fda*	Carbohydrate degradation	Fructose-bisphosphate aldolase class 1	−2.21
*ldh2*	Fermentation	L-lactate dehydrogenase 2 (L-LDH 2)	−2.44
*rocF*	Nitrogen metabolism	Arginase	−2.06
*glmU*	Nucleotide-sugar biosynthesis	Bifunctional protein GlmU	−2.01
*hemB*	Porphyrin-containing compound metabolism	Delta-aminolevulinic acid dehydratase	−2.24
*purH*	Purine metabolism	Bifunctional purine biosynthesis protein PurH	−3.97
*purM*	Purine metabolism	Phosphoribosylformylglycinamidine cyclo-ligase	−2.54
*purN*	Purine metabolism	Phosphoribosylglycinamide formyltransferase	−2.21
*purD*	Purine metabolism	Phosphoribosylamine—glycine ligase	−2.10
*pyrC*	Pyrimidine metabolism	Dihydroorotase (DHOase)	−2.68
*pyrE*	Pyrimidine metabolism	Orotate phosphoribosyltransferase (OPRT)	−2.04
*serS*	tRNA aminoacylation	Serine—tRNA ligase	−2.49

**TABLE 2:** Pathway assigned up/downregulated genes for TCH1516 under moxifloxacin treatment

Gene name	Pathway	Protein name	log2 Fold change (FC)
Pathway assigned upregulated genes			
*arcB (argF*)	Amino-acid biosynthesis	Ornithine carbamoyltransferase (OTCase)	3.40
*leuD*	Amino-acid biosynthesis	3-Isopropylmalate dehydratase small subunit	2.31
*ltaS*	Cell wall biogenesis	Lipoteichoic acid synthase	2.88
*tagA*	Cell wall biogenesis	N-acetylglucosaminyldiphosphoundecaprenol	2.08
*ribBA*	Cofactor biosynthesis	Riboflavin biosynthesis protein RibBA	2.82
*panD*	Cofactor biosynthesis	Aspartate 1-decarboxylase	2.65
*accB*	Lipid metabolism	Biotin carboxyl carrier protein of acetyl-CoA carboxylase	3.61
*acpP*	Lipid metabolism	Acyl carrier protein (ACP)	2.43
*RelA/SpoT*	Purine metabolism; ppGpp biosynthesis	RelA/SpoT domain-containing protein	2.72
*efb*	*Staphylococcus aureus* infection	Fibrinogen-binding protein	3.42
*A0A0H2XHQ0*	Teichoic acid biosynthesis	Putative transcriptional regulator	2.90
*vraR*	Two-component system	DNA-binding response regulator	2.57
*A0A0H2XF42*	Two-component system	Cytochrome D ubiquinol oxidase, subunit I	4.30
*vraS*	Two-component system	Sensor protein VraS	4.41
*vraT*	Two-component system	Cell wall-active antibiotics response LiaF-like C-terminal domain-containing protein	3.97
Pathway assigned downregulated genes			
*asd*	Amino-acid biosynthesis	Aspartate-semialdehyde dehydrogenase (ASA dehydrogenase)	−13.20
*dapH*	Amino-acid biosynthesis	2,3,4,5-tetrahydropyridine-2,6-dicarboxylate N-acetyltransferase	−7.54
*ilvC*	Amino-acid biosynthesis	Ketol-acid reductoisomerase (NADP(+)) (KARI)	−6.10
*ilvA*	Amino-acid biosynthesis	L-threonine dehydratase biosynthetic IlvA	−5.89
*dapA*	Amino-acid biosynthesis	4-hydroxy-tetrahydrodipicolinate synthase (HTPA synthase)	−5.68
*ilvB*	Amino-acid biosynthesis	Acetolactate synthase	−4.77
*A0A0H2XHR4*	Amino-acid biosynthesis	Homoserine dehydrogenase	−4.34
*thrC*	Amino-acid biosynthesis	Threonine synthase	−3.60
*serA*	Amino-acid biosynthesis	D-3-phosphoglycerate dehydrogenase	−3.57
*dapB*	Amino-acid biosynthesis	4-Hydroxy-tetrahydrodipicolinate reductase (HTPA reductase)	−3.55
*A0A0H2XKA6*	Amino-acid biosynthesis	Probable succinyl-diaminopimelate desuccinylase	−3.45
*ilvE*	Amino-acid biosynthesis	Branched-chain-amino-acid aminotransferase	−3.36
*thrB*	Amino-acid biosynthesis	Homoserine kinase (HK)	−3.16
*metE*	Amino-acid biosynthesis	5-Methyltetrahydropteroyltriglutamate—homocysteine methyltransferase	−2.94
*proC*	Amino-acid biosynthesis	Pyrroline-5-carboxylate reductase (P5C reductase)	−2.03
*odhB sucB*	Amino-acid degradation	Dihydrolipoyllysine-residue succinyltransferase component of 2-oxoglutarate dehydrogenase complex	−2.17
*tpiA*	Carbohydrate biosynthesis	Triosephosphate isomerase (TIM)	−2.53
*acnA*	Carbohydrate metabolism	Aconitate hydratase (Aconitase)	−2.38
*aroA*	Metabolic intermediate biosynthesis	3-Phosphoshikimate 1-carboxyvinyltransferase	−2.15
*folD*	One-carbon metabolism	Bifunctional protein FolD	−2.86
*efp*	Protein biosynthesis	Elongation factor *P* (EF-P)	−2.47
*purS*	Purine metabolism	Phosphoribosylformylglycinamidine synthase subunit PurS (FGAM synthase)	−4.68
*purM*	Purine metabolism	Phosphoribosylformylglycinamidine cyclo-ligase	−4.13
*purN*	Purine metabolism	Phosphoribosylglycinamide formyltransferase	−3.68
*purH*	Purine metabolism	Bifunctional purine biosynthesis protein PurH	−3.56
*purQ*	Purine metabolism	Phosphoribosylformylglycinamidine synthase subunit PurQ (FGAM synthase)	−3.48
*purC*	Purine metabolism	Phosphoribosylaminoimidazole-succinocarboxamide synthase	−3.17
*purL*	Purine metabolism	Phosphoribosylformylglycinamidine synthase subunit PurL (FGAM synthase)	−3.14
*purD*	Purine metabolism	Phosphoribosylamine—glycine ligase	−2.71
*purF*	Purine metabolism	Amidophosphoribosyltransferase (ATase)	−2.01
*serS*	tRNA aminoacylation	Serine—tRNA ligase	−2.55

**TABLE 3:** Pathway assigned up/downregulated genes for 880 under oxacillin treatment

Gene name	Pathway	Protein name	.–log 10 (*P*-value)	log2 Fold change (FC)
Pathway assigned upregulated genes				
*A0A0H2XK42*	ABC transporters	Amino acid ABC transporter, amino acid-binding protein	5.60	4.51
*tagH*	ABC transporters	Teichoic acids export ATP-binding protein TagH	6.36	3.27
*alr2*	Amino-acid biosynthesis	Alanine racemase	6.86	4.10
*putA*	Amino-acid degradation	Proline dehydrogenase	4.43	2.95
*mecA*	Beta-lactam resistance	Penicillin-binding protein 2	5.43	2.86
*mgt*	Cell wall biogenesis	Monofunctional glycosyltransferase (MGT)	7.07	3.98
*panD*	Cofactor biosynthesis	Aspartate 1-decarboxylase	3.05	2.38
*uppS*	Peptidoglycan biosynthesis	Isoprenyl transferase	4.60	2.03
*rpsN rpsN2*	Ribosome	Small ribosomal subunit protein uS14A (30S ribosomal protein S14)	5.27	3.00
*sbi*	*Staphylococcus aureus* infection	Immunoglobulin-binding protein Sbi	6.92	3.65
*efb*	*Staphylococcus aureus* infection	Fibrinogen-binding protein	5.53	4.05
*msrR*	Teichoic acid biosynthesis	Regulatory protein MsrR	8.48	5.65
*A0A0H2XHQ0*	Teichoic acid biosynthesis	Putative transcriptional regulator	9.72	4.10
*vraS*	Two-component system	Sensor protein VraS	8.22	3.35
*vraT*	Two-component system	Cell wall-active antibiotics response LiaF-like C-terminal domain-containing protein	5.17	3.56
Pathway assigned downregulated genes				
*carB*	Amino-acid biosynthesis	Carbamoyl phosphate synthase large chain	8.35	−3.17
*carA*	Amino-acid biosynthesis	Carbamoyl phosphate synthase small chain	7.67	−3.74
*trpD*	Amino-acid biosynthesis	Anthranilate phosphoribosyltransferase	4.72	−2.62
*argR*	Amino-acid biosynthesis	Arginine repressor	7.90	−2.44
*pheA*	Amino-acid biosynthesis	Prephenate dehydratase	5.82	−3.03
*ald1*	Amino-acid degradation	Alanine dehydrogenase 1	4.82	−4.67
*tdcB*	Amino-acid degradation	L-threonine dehydratase catabolic TdcB	3.73	−4.59
*hutG*	Amino-acid degradation	Formimidoylglutamase	6.87	−3.09
*hutU*	Amino-acid degradation	Urocanate hydratase (Urocanase)	4.46	−3.03
*arcA*	Amino-acid degradation	Arginine deiminase (ADI)	4.95	−3.00
*nanE*	Amino-sugar metabolism	Putative N-acetylmannosamine-6-phosphate 2-epimerase	8.17	−2.66
*nagB*	Amino-sugar metabolism	Glucosamine-6-phosphate deaminase	7.33	−2.23
*sdaAB*	Carbohydrate biosynthesis	L-serine deaminase	6.59	−3.24
*tpiA*	Carbohydrate biosynthesis	Triosephosphate isomerase (TIM)	7.20	−2.38
*tkt*	Carbohydrate biosynthesis	Transketolase	8.16	−2.20
*rpiA*	Carbohydrate degradation	Ribose-5-phosphate isomerase A	10.41	−5.77
*fda*	Carbohydrate degradation	Fructose-bisphosphate aldolase class 1	9.59	−3.66
*pfkA*	Carbohydrate degradation	ATP-dependent 6-phosphofructokinase (ATP-PFK)	8.77	−3.31
*rpe*	Carbohydrate degradation	Ribulose-phosphate 3-epimerase	7.42	−2.60
*fba*	Carbohydrate degradation	Fructose-bisphosphate aldolase	8.28	−2.38
*deoC*	Carbohydrate degradation	Deoxyribose-phosphate aldolase (DERA)	2.95	−2.84
*gpmI*	Carbohydrate degradation	2,3-bisphosphoglycerate-independent phosphoglycerate mutase (BPG-independent PGAM)	6.36	−3.00
*pgk*	Carbohydrate degradation	Phosphoglycerate kinase	6.80	−2.22
*eno*	Carbohydrate degradation	Enolase	6.93	−2.18
*gap*	Carbohydrate degradation	Glyceraldehyde-3-phosphate dehydrogenase	7.73	−2.17
*pyk*	Carbohydrate degradation	Pyruvate kinase (PK)	8.30	−2.17
*acnA*	Carbohydrate metabolism	Aconitate hydratase (Aconitase)	9.72	−3.21
*galM*	Carbohydrate metabolism	Aldose 1-epimerase	6.83	−2.75
*mqo*	Carbohydrate metabolism	Probable malate:quinone oxidoreductase	5.18	−2.72
*rbsK*	Carbohydrate metabolism	Ribokinase (RK)	6.20	−2.36
*crtN*	Carotenoid biosynthesis	4,4′-diapophytoene desaturase	4.00	−2.66
*dltC*	Cell wall biogenesis	D-alanyl carrier protein (DCP)	9.74	−4.54
*tarJ*	Cell wall biogenesis	Ribulose-5-phosphate reductase (Ribulose-5-P reductase)	4.63	−2.24
*dltB*	Cell wall biogenesis	Teichoic acid D-alanyltransferase	4.48	−2.09
*tarI*	Cell wall biogenesis	Ribitol-5-phosphate cytidylyltransferase	7.21	−2.07
*folB*	Cofactor biosynthesis	7,8-dihydroneopterin aldolase	5.80	−4.09
*moaB*	Cofactor biosynthesis	Molybdenum cofactor biosynthesis protein B	4.23	−3.31
*moaE*	Cofactor biosynthesis	Molybdopterin synthase catalytic subunit	5.93	−3.03
*pdxT*	Cofactor biosynthesis	Pyridoxal 5′-phosphate synthase subunit PdxT	9.11	−3.02
*pdxS*	Cofactor biosynthesis	Pyridoxal 5′-phosphate synthase subunit PdxS	7.51	−2.85
*coaE*	Cofactor biosynthesis	Dephospho-CoA kinase	7.74	−2.62
*nadE*	Cofactor biosynthesis	NH(3)-dependent NAD(+) synthetase	8.77	−2.49
*A0A0H2XI81*	Cofactor biosynthesis	Nicotinate phosphoribosyltransferase	7.05	−2.45
*moeA*	Cofactor biosynthesis	Molybdopterin molybdenum transferase	7.87	−2.36
*ldh2*	Fermentation	L-lactate dehydrogenase 2 (L-LDH 2)	11.36	−3.80
*ldh1*	Fermentation	L-lactate dehydrogenase 1 (L-LDH 1)	8.58	−3.71
*gtaB galU*	Glycolipid metabolism	UTP--glucose-1-phosphate uridylyltransferase	11.22	−4.22
*acpP*	Lipid metabolism	Acyl carrier protein (ACP)	6.50	−2.95
*ackA*	Metabolic intermediate biosynthesis	Acetate kinase	9.65	−3.11
*aroK*	Metabolic intermediate biosynthesis	Shikimate kinase (SK)	5.63	−2.46
*aroC*	Metabolic intermediate biosynthesis	Chorismate synthase (CS)	7.41	−2.18
*prs*	Metabolic intermediate biosynthesis	Ribose-phosphate pyrophosphokinase (RPPK)	5.26	−2.09
*deoB*	Metabolic intermediate biosynthesis	Phosphopentomutase	7.67	−2.02
*A0A0H2XH90*	Metabolic intermediate metabolism	3-Hydroxy-3-methylglutaryl coenzyme A reductase (HMG-CoA reductase)	7.52	−2.14
*nirB*	Nitrogen metabolism	Nitrite reductase [NAD(P)H], large subunit	9.49	−3.21
*glmU*	Nucleotide-sugar biosynthesis	Bifunctional protein GlmU	5.59	−3.12
*folD*	One-carbon metabolism	Bifunctional protein FolD	9.96	−2.83
*fhs*	One-carbon metabolism	Formate—tetrahydrofolate ligase	8.05	−2.63
*budA*	Polyol metabolism	Alpha-acetolactate decarboxylase	9.13	−5.20
*hemB*	Porphyrin-containing compound metabolism	Delta-aminolevulinic acid dehydratase	9.92	−3.35
*hemD*	Porphyrin-containing compound metabolism	Uroporphyrinogen-III synthase	4.49	−2.85
*hemC*	Porphyrin-containing compound metabolism	Porphobilinogen deaminase (PBG)	9.93	−2.70
*hemG*	Porphyrin-containing compound metabolism	Coproporphyrinogen III oxidase	6.50	−2.48
*efp*	Protein biosynthesis	Elongation factor P (EF-P)	6.77	−2.62
*lipA*	Protein modification	Lipoyl synthase (Lip-syn)	6.36	−2.08
*lgt*	Protein modification	Phosphatidylglycerol—prolipoprotein diacylglyceryl transferase	2.91	−2.04
*purS*	Purine metabolism	Phosphoribosylformylglycinamidine synthase subunit PurS (FGAM synthase)	8.35	−5.47
*purH*	Purine metabolism	Bifunctional purine biosynthesis protein PurH	10.95	−5.45
*purN*	Purine metabolism	Phosphoribosylglycinamide formyltransferase	9.24	−5.32
*purM*	Purine metabolism	Phosphoribosylformylglycinamidine cyclo-ligase	9.79	−5.00
*purQ*	Purine metabolism	Phosphoribosylformylglycinamidine synthase subunit PurQ (FGAM synthase)	9.04	−4.57
*xpt*	Purine metabolism	Xanthine phosphoribosyltransferase (XPRTase)	9.22	−4.11
*purC*	Purine metabolism	Phosphoribosylaminoimidazole-succinocarboxamide synthase	10.22	−4.03
*purE*	Purine metabolism	N5-carboxyaminoimidazole ribonucleotide mutase (N5-CAIR mutase)	8.03	−3.86
*purD*	Purine metabolism	Phosphoribosylamine—glycine ligase	9.32	−3.86
*purL*	Purine metabolism	Phosphoribosylformylglycinamidine synthase subunit PurL (FGAM synthase)	11.59	−3.81
*purF*	Purine metabolism	Amidophosphoribosyltransferase (ATase)	7.93	−3.22
*A0A0H2XFD7*	Purine metabolism	6-carboxy-5,6,7,8-tetrahydropterin synthase	5.43	−3.07
*guaB*	Purine metabolism	Inosine-5'-monophosphate dehydrogenase (IMP dehydrogenase)	9.52	−3.05
*hpt*	Purine metabolism	Hypoxanthine phosphoribosyltransferase	7.72	−2.98
*purK*	Purine metabolism	N5-carboxyaminoimidazole ribonucleotide synthase (N5-CAIR synthase)	9.20	−2.86
*purA*	Purine metabolism	Adenylosuccinate synthetase (AdSS)	8.27	−2.58
*pyrF*	Pyrimidine metabolism	Orotidine 5′-phosphate decarboxylase	11.33	−5.03
*pyrC*	Pyrimidine metabolism	Dihydroorotase (DHOase)	8.71	−4.12
*pyrE*	Pyrimidine metabolism	Orotate phosphoribosyltransferase (OPRT)	7.04	−3.40
*pyrB*	Pyrimidine metabolism	Aspartate carbamoyltransferase catalytic subunit	6.33	−3.32
*pyrH*	Pyrimidine metabolism	Uridylate kinase (UK)	7.32	−2.58
*menB*	Quinol/quinone metabolism	1,4-dihydroxy-2-naphthoyl-CoA synthase (DHNA-CoA synthase)	9.80	−2.82
*menH*	Quinol/quinone metabolism	Putative 2-succinyl-6-hydroxy-2,4-cyclohexadiene-1-carboxylate synthase (SHCHC synthase)	3.93	−2.11
*serS*	tRNA aminoacylation	Serine—tRNA ligase	11.10	−4.10
*queH*	tRNA modification	Epoxyqueuosine reductase QueH	7.01	−3.31
*queA*	tRNA modification	S-adenosylmethionine:tRNA ribosyltransferase-isomerase	3.47	−2.04

**TABLE 4:** Pathway assigned upregulated/downregulated genes for 880 under moxifloxacin treatment

Gene name	Pathway	Protein name	log2 Fold change (FC)
Pathway assigned upregulated genes			
*A0A0H2XK42*	ABC transporters	Amino acid ABC transporter	5.45
*tagH*	ABC transporters	Teichoic acids export ATP-binding protein TagH	2.47
*alr2*	Amino-acid biosynthesis	Alanine racemase	4.61
*putA*	Amino-acid degradation	Proline dehydrogenase	3.84
*fnbA*	Bacterial invasion of epithelial cells	Fibronectin-binding protein A	4.41
*fnbB*	Bacterial invasion of epithelial cells	Fibronectin-binding protein B	3.53
*mecA*	Beta-Lactam resistance	Penicillin-binding protein 2	2.18
*dltD*	Cell wall biogenesis	Protein DltD	3.37
*murP*	Cell wall biogenesis	PTS system MurNAc-GlcNAc-specific EIIBC component	2.15
*thiI*	Cofactor biosynthesis	Probable tRNA sulfurtransferase	4.67
*ribBA*	Cofactor biosynthesis	Riboflavin biosynthesis protein RibBA	3.02
*ribD*	Cofactor biosynthesis	Riboflavin biosynthesis protein RibD	2.66
*ribH*	Cofactor biosynthesis	6,7-Dimethyl-8-ribityllumazine synthase (DMRL synthase)	2.66
*qoxB*	Energy metabolism	Probable quinol oxidase subunit 1	3.30
*accB*	Lipid metabolism	Biotin carboxyl carrier protein of acetyl-CoA carboxylase	2.80
*sbi*	*Staphylococcus aureus* infection	Immunoglobulin-binding protein Sbi	4.66
*scn*	*Staphylococcus aureus* infection	Staphylococcal complement inhibitor (SCIN)	4.28
*clfB*	*Staphylococcus aureus* infection	Clumping factor B	3.89
*sdrE*	*Staphylococcus aureus* infection	Serine-aspartate repeat-containing protein E	3.70
*clfA*	*Staphylococcus aureus* infection	Clumping factor A	3.70
*vraT*	Two-component system	Cell wall-active antibiotics response LiaF-like C-terminal domain-containing protein	2.03
Pathway assigned downregulated genes			
*hisC*	Amino acid biosynthesis	Histidinol-phosphate aminotransferase	−6.70
*A0A0H2XHR4*	Amino acid biosynthesis	Homoserine dehydrogenase	−6.30
*dapA*	Amino acid biosynthesis	4-Hydroxy-tetrahydrodipicolinate synthase (HTPA synthase)	−6.24
*dapB*	Amino acid biosynthesis	4-Hydroxy-tetrahydrodipicolinate reductase (HTPA reductase)	−5.91
*serA*	Amino-acid biosynthesis	D-3-phosphoglycerate dehydrogenase	−5.06
*ilvE*	Amino-acid biosynthesis	Branched-chain-amino-acid aminotransferase	−4.42
*asd*	Amino-acid biosynthesis	Aspartate-semialdehyde dehydrogenase (ASA dehydrogenase)	−4.29
*proC*	Amino-acid biosynthesis	Pyrroline-5-carboxylate reductase (P5C reductase)	−3.60
*thrC*	Amino-acid biosynthesis	Threonine synthase	−3.58
*dapH*	Amino-acid biosynthesis	2,3,4,5-tetrahydropyridine-2,6-dicarboxylate N-acetyltransferase	−3.28
*trpD*	Amino acid biosynthesis	Anthranilate phosphoribosyltransferase	−3.02
*lysA*	Amino acid biosynthesis	Diaminopimelate decarboxylase (DAP decarboxylase)	−3.00
*argR*	Amino acid biosynthesis	Arginine repressor	−2.98
*carA*	Amino acid biosynthesis	Carbamoyl phosphate synthase small chain	−2.76
*pheA*	Amino acid biosynthesis	Prephenate dehydratase	−2.60
*carB*	Amino acid biosynthesis	Carbamoyl phosphate synthase large chain	−2.35
*ald1*	Amino acid degradation	Alanine dehydrogenase 1	−4.00
*tdcB*	Amino acid degradation	L-threonine dehydratase catabolic TdcB	−3.36
*nagB*	Amino-sugar metabolism	Glucosamine-6-phosphate deaminase	−2.35
*nanE*	Amino-sugar metabolism	Putative N-acetylmannosamine-6-phosphate 2-epimerase	−2.27
*tpiA*	Carbohydrate biosynthesis	Triosephosphate isomerase (TIM)	−2.71
*pgi*	Carbohydrate biosynthesis	Glucose-6-phosphate isomerase (GPI)	−2.47
*rpiA*	Carbohydrate degradation	Ribose-5-phosphate isomerase A	−3.63
*fda*	Carbohydrate degradation	Fructose-bisphosphate aldolase class 1	−3.15
*pfkA*	Carbohydrate degradation	ATP-dependent 6-phosphofructokinase	−2.79
*pgk*	Carbohydrate degradation	Phosphoglycerate kinase	−2.72
*rpe*	Carbohydrate degradation	Ribulose-phosphate 3-epimerase	−2.69
*gpmI*	Carbohydrate degradation	2,3-bisphosphoglycerate-independent phosphoglycerate mutase	−2.43
*gpmA*	Carbohydrate degradation	2,3-bisphosphoglycerate-dependent phosphoglycerate mutase	−2.29
*deoC*	Carbohydrate degradation	Deoxyribose-phosphate aldolase	−2.24
*acnA*	Carbohydrate metabolism	Aconitate hydratase (Aconitase)	−2.93
*rbsK*	Carbohydrate metabolism	Ribokinase (RK)	−2.72
*galM*	Carbohydrate metabolism	Aldose 1-epimerase	−2.39
*fumC*	Carbohydrate metabolism	Fumarate hydratase class II (Fumarase C)	−2.23
*dltC*	Cell wall biogenesis	D-alanyl carrier protein (DCP)	−6.92
*tarI*	Cell wall biogenesis	Ribitol-5-phosphate cytidylyltransferase	−5.25
*murB*	Cell wall biogenesis	UDP-N-acetylenolpyruvoylglucosamine reductase	−3.18
*tarJ*	Cell wall biogenesis	Ribulose-5-phosphate reductase (Ribulose-5-P reductase)	−2.81
*ddl*	Cell wall biogenesis	D-alanine—D-alanine ligase	−2.23
*moaD*	Cofactor biosynthesis	Molybdopterin synthase sulfur carrier subunit	−4.80
*folB*	Cofactor biosynthesis	7,8-dihydroneopterin aldolase	−4.29
*folA*	Cofactor biosynthesis	Dihydrofolate reductase	−3.47
*pdxT*	Cofactor biosynthesis	Pyridoxal 5′-phosphate synthase subunit PdxT	−3.08
*nadE*	Cofactor biosynthesis	NH(3)-dependent NAD(+) synthetase	−3.00
*panB*	Cofactor biosynthesis	3-methyl-2-oxobutanoate hydroxymethyltransferase	−2.82
*folP*	Cofactor biosynthesis	Dihydropteroate synthase (DHPS)	−2.52
*pdxS*	Cofactor biosynthesis	Pyridoxal 5'-phosphate synthase subunit PdxS	−2.26
*folK*	Cofactor biosynthesis	2-Amino-4-hydroxy-6-hydroxymethyldihydropteridine diphosphokinase	−2.18
*coaE*	Cofactor biosynthesis	Dephospho-CoA kinase	−2.11
*ribF*	Cofactor biosynthesis	Riboflavin biosynthesis protein	−2.05
*coaW coaA*	Cofactor biosynthesis	Type II pantothenate kinase	−2.04
*coaD*	Cofactor biosynthesis	Phosphopantetheine adenylyltransferase	−2.02
*ldh2*	Fermentation	L-lactate dehydrogenase 2 (L-LDH 2)	−4.18
*gtaB galU*	Glycolipid metabolism	UTP—glucose-1-phosphate uridylyltransferase	−2.94
*fabD*	Lipid metabolism	Malonyl CoA-acyl carrier protein transacylase	−2.31
*acpP*	Lipid metabolism	Acyl carrier protein (ACP)	−2.20
*plsX*	Lipid metabolism	Phosphate acyltransferase	−2.16
*aroK*	Metabolic intermediate biosynthesis	Shikimate kinase (SK)	−2.67
*deoB*	Metabolic intermediate biosynthesis	Phosphopentomutase	−2.08
*A0A0H2XH90*	Metabolic intermediate metabolism	3-Hydroxy-3-methylglutaryl coenzyme A reductase	−2.75
*rocF*	Nitrogen metabolism	Arginase	−4.84
*nirB*	Nitrogen metabolism	Nitrite reductase [NAD(P)H], large subunit	−2.23
*folD*	One-carbon metabolism	Bifunctional protein FolD	−4.00
*fhs*	One-carbon metabolism	Formate—tetrahydrofolate ligase	−2.28
*Q2FJ70*	One-carbon metabolism	3-hexulose-6-phosphate synthase (HPS)	−2.12
*budA*	Polyol metabolism	Alpha-acetolactate decarboxylase	−3.44
*budA*	Polyol metabolism	Alpha-acetolactate decarboxylase	−2.32
*hemH cpfC*	Porphyrin-containing compound metabolism	Coproporphyrin III ferrochelatase	−2.44
*hemB*	Porphyrin-containing compound metabolism	Delta-aminolevulinic acid dehydratase	−2.23
*efp*	Protein biosynthesis	Elongation factor P (EF-P)	−2.65
*A0A0H2XFJ1*	Protein modification	Lipoate—protein ligase	−3.49
*lipA*	Protein modification	Lipoyl synthase	−3.44
*purS*	Purine metabolism	Phosphoribosylformylglycinamidine synthase subunit PurS	−6.97
*purN*	Purine metabolism	Phosphoribosylglycinamide formyltransferase	−5.71
*purQ*	Purine metabolism	Phosphoribosylformylglycinamidine synthase subunit PurQ	−5.33
*purC*	Purine metabolism	Phosphoribosylaminoimidazole-succinocarboxamide synthase	−5.04
*purM*	Purine metabolism	Phosphoribosylformylglycinamidine cyclo-ligase	−4.34
*purH*	Purine metabolism	Bifunctional purine biosynthesis protein PurH	−3.95
*purD*	Purine metabolism	Phosphoribosylamine—glycine ligase	−3.75
*xpt*	Purine metabolism	Xanthine phosphoribosyltransferase (XPRTase)	−3.27
*purL*	Purine metabolism	Phosphoribosylformylglycinamidine synthase subunit PurL	−3.24
*purE*	Purine metabolism	N5-carboxyaminoimidazole ribonucleotide mutase	−2.79
*adk*	Purine metabolism	Adenylate kinase (AK)	−2.53
*hpt*	Purine metabolism	Hypoxanthine phosphoribosyltransferase	−2.52
*purF*	Purine metabolism	Amidophosphoribosyltransferase (ATase)	−2.48
*purA*	Purine metabolism	Adenylosuccinate synthetase (AMPSase)	−2.20
*purK*	Purine metabolism	N5-carboxyaminoimidazole ribonucleotide synthase	−2.07
*pyrE*	Pyrimidine metabolism	Orotate phosphoribosyltransferase (OPRT)	−3.70
*pyrC*	Pyrimidine metabolism	Dihydroorotase (DHOase)	−3.61
*pyrF*	Pyrimidine metabolism	Orotidine 5′-phosphate decarboxylase	−3.52
*pyrB*	Pyrimidine metabolism	Aspartate carbamoyltransferase catalytic subunit	−2.09
*menH*	Quinol/quinone metabolism	Putative 2-succinyl-6-hydroxy-2,4-cyclohexadiene-1-carboxylate synthase	−2.20
*menB*	Quinol/quinone metabolism	1,4-Dihydroxy-2-naphthoyl-CoA synthase	−2.11
*serS*	tRNA aminoacylation	Serine—tRNA ligase	−2.79
*queH*	tRNA modification	Epoxyqueuosine reductase QueH	−2.31

### Identification of known persistence pathways

To validate our experimental approach in comparison to other published experimental methods, we screened for genes and pathways that have been previously found to be upregulated or downregulated in SA persisters (9, 11, 23). We only briefly mention these here and provide a more detailed analysis later on. As expected, many “known” persister genes were observed for at least one of the combinations of strain and antibiotic, although few were conserved over all test conditions. In common with other studies, we observed changes in the stringent response, via upregulation of RelA/SpoT (23, 24) (albeit only in TCH1516, but not in strain 880), as well as significant increases in the production of proteins associated with cell wall stress and integrity, including VraSTR (25). Diverse aspects of metabolism, especially related to purine (26) and amino acid production and degradation, were significantly changed (13, 27). Similar to Liu et al., who used cell division and cell wall integrity to FACS-sort persister cells treated with enrofloxacin and vancomycin, we found significant ribosome downregulation, although to a lesser degree, which could be due to the fact they enriched for translationally and divisionally inactive cells, while BONCAT does not require this sorting step (13, 29). In common with Huemer et al. (82), we observed a reduction in virulence-associated proteins during persistence, including a marked reduction in the type VII secretion system, particularly in strain TCH1516. These, and further, retrieval of known persister proteins, shown in Tables 1 to 4; Table S1 to S4, gave us confidence in the ability of BONCAT labeling to retrieve persister proteins in a single set of experiments without any genetic modification or delay between inducing persistence and analysis.

### Strain and drug-specific effects

There was extensive variation in the specific proteins that were upregulated and downregulated between strains and antibiotics, suggesting that persistence is highly strain and antibiotic-specific. Nevertheless, common pathways and some common proteins are evident. Most notably, the VraRTS pathway was upregulated under all conditions. This pathway, which is a regulator of cell wall biosynthesis and stress, causes cell wall thickening and is commonly mutated in strains with reduced sensitivity to vancomycin (83). VraT was upregulated in all samples, as was VraS (more than twofold increases in three of four samples). Related changes to cell wall and peptidoglycan biosynthesis were also observed, especially in both strains exposed to oxacillin, including upregulation of *mgt*, *mecA* (in 880), *pbp2* (in TCH1516), *murP,* and *uppS*. In addition, there was a common downregulation of putative virulence factors, including many proteins associated with the Type VII secretion system (84), especially in TCH1516. Of the 74 downregulated proteins in response to oxacillin, we observed a clear excess of proteins associated with the degradation of amino acids, purines, pyrimidines, and carbohydrates (Tables 1 and 3), all consistent with the idea of general growth arrest, decreased translation and DNA replication, and energy conservation in persisters. Related to this are common changes in ABC transporters. Some ABC transporters were upregulated, but also many—particularly the peptide transporters—were downregulated across three of four conditions, suggesting that nutrient restriction may be an active route toward persistence. Many of the global changes to cell wall biosynthesis, the stringent response, and energy metabolism were also observed in strains exposed to moxifloxacin, indicating the conserved phenotypic features of persisters, even if the identity of modified proteins may vary between strains and antibiotics.

In addition to common changes in persisters, we observed many more proteome changes that were limited to either strain or antibiotic, reflecting differences between genotypes or the physiological responses elicited by different antibiotics. For example, we observed upregulation of numerous putative phage-associated genes in TCH1516 in moxifloxacin persisters, but not during oxacillin exposure, consistent with the DNA-damaging effects of the former antibiotic. Downregulation of ribosomal proteins was more evident in 880 exposed to oxacillin. Overall, there were many more unique changes than shared responses (Fig. 2e), including many changes in uncharacterized proteins (Tables S1 to S4). The identification of known pathways means this list can serve as an excellent resource for new target validation. A final interesting point was the change in the transcription factor landscape. More than 29 transcription factors, or other DNA-binding proteins, were upregulated, while 35 transcription factors/DNA-binding proteins were downregulated in each of the conditions, each of which may pleiotropically influence the production of many other genes, although further validation of these “hits” is required to fully confirm their role in persister biology.

### Temporal changes in the persister protein landscape

**Fig 3:**
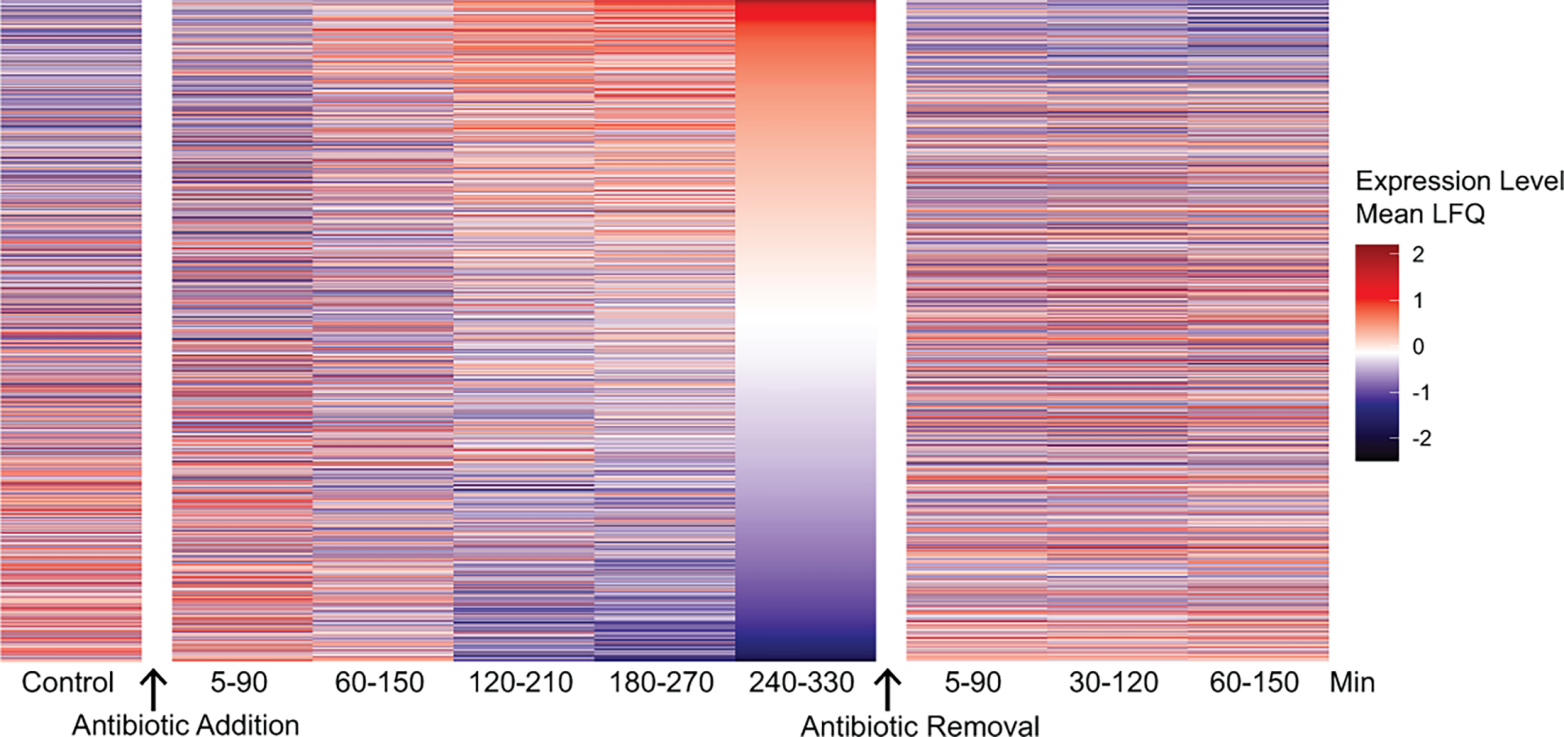
Heatmap showing the changes in protein expression over time. All identified changed proteins of strain TCH1516 at 4 h were also analyzed and plotted at the indicated time. Associated IDs and values are shown in Table S8.

**Fig 4:**
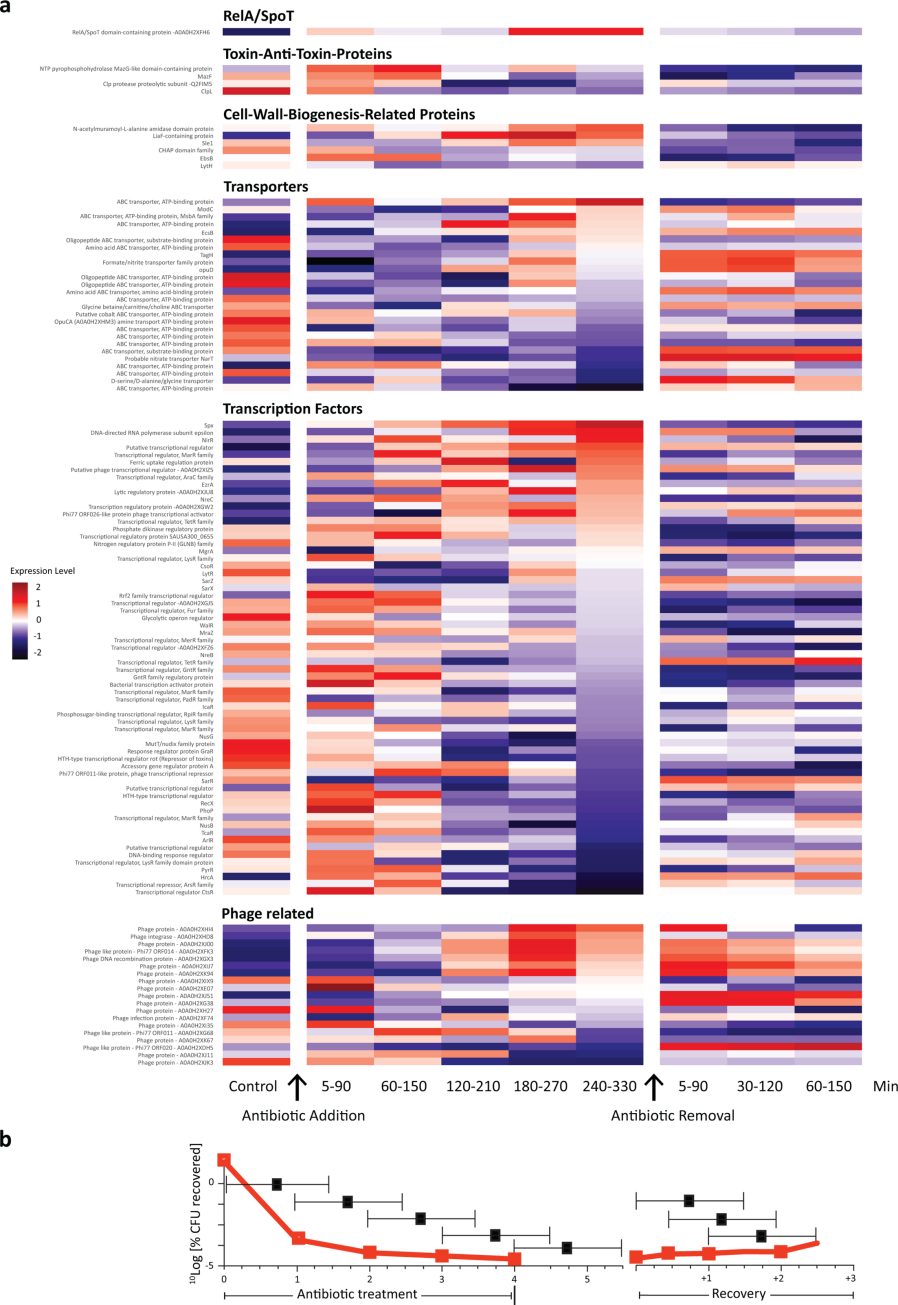
Changes in expression over time. (**a**) Heatmap showing the change in protein expression of key proteins comparing controls, different time ranges after antibiotic challenge (i.e., entry into persister state) and departure from the persister state (after antibiotic removal) of strain TCH1516 treated with moxifloxacin. Only RelA/SpoT, cell wall biogenesis-associated proteins, transporters, and transcription factors are shown. The proteins have been ordered according to relative expression at t = 4 h. A full table of all proteins is shown in Table S9. (**b**) % CFU recovered at the indicated timepoints.

After quantifying proteome changes at a single time point, we next set up a time course to characterize how persister-associated proteins changed dynamically during entry into and exit from the persister state. To do so, we focused on TCH1516 exposed to moxifloxacin because the persister fraction in this combination was low and could therefore test the limits of the BONCAT approach. A first comparison of protein expression at the earliest time point after antibiotic addition (on the steep stage of the time-kill curve) showed the upregulation of 211 proteins compared to the persister state at 4 h for this same strain-antibiotic combination, and the downregulation of 30 (Table S5), highlighting the difference between the early stress response and the persister state. To further characterize the temporal changes in the proteome, we applied the Aha pulse at different times after the addition of the high-dose antibiotic (0–90 min, 60–150 min, 120–210 min, 180–270 min, and 240–330 min), prior to lysis, retrieval, and LFQ-LCMS (Fig. 3). To study protein expression upon exit of the persister phase, we removed the antibiotic-containing medium and replaced it with fresh medium—which was done by centrifugation, washing, and resuspension—and gave the Aha-pulse 5–90 min, 30–120 min, and 60–150 min after removal of the antibiotic. The pulsed samples were again lysed at the end of the Aha-pulse period and analyzed by the ccHc-LFQ-MS protocol as before. Figure 3 shows the expression of *all* changed proteins ordered by the relative expression at 4 hours. The data supporting this figure can be found in supplementary spreadsheet Table S8. Data shown in Fig. 4a are ranked according to the 4 h time point to focus attention on (selected) proteins that have undergone the most significant changes. These results also highlight that different proteins vary in the time required for upregulation or downregulation to become evident.

Aside from global changes in Fig. 3, we selectively plotted the dynamics of some of the key proteins known to be important in persisters or that showed prominent changes in the 4 h samples, specifically RelA/SpoT, toxin-anti-toxin proteins, cell wall biosynthesis proteins, transporters, various transcription factors, and phage-associated proteins (Fig. 4a, and for a full list and the relative expression values, see supplementary spreadsheet Table S9). This time-course analysis showed that changes to persister-associated proteins show distinct temporal dynamics. For example, RelA/SpotT expression was rapidly induced and continued increasing during antibiotic exposure and slowly declined after antibiotic removal. Several, but not all, transcription factors showed similar dynamics, although with less consistent behavior after antibiotic removal. These changes are particularly interesting in light of the highly pleiotropic effects of transcription factors across the *S. aureus* proteome. For example, the stress-induced transcriptional regulator, *spx*, showed rapid increases upon antibiotic exposure. This, in turn, likely downregulated other gene products coordinated by *spx*, like *clp* proteases and the MazEF toxin-anti-toxin system. The role of this TA module in *S. aureus* persistence is controversial. In contrast to other systems where the antitoxin component is down-regulated, we instead observed a short-term increase in the MazF toxin and the associated antitoxin protease ClpL, followed by a sharp decrease in production of both. ClpL may also impact general stress responses during antibiotic exposure (85, 86), along with other changes that affect protein stability and quality control, for example, *ctsR* and *hrcA*. Extracellular protease production may also be influenced by changes in the expression of the *sarR* regulon, which is initially highly expressed, is rapidly downregulated during drug exposure, and then quickly increases during recovery.

We observed changes in several proteins that influence virulence factors, including some indicated above. ArlR is part of a two-component system regulating autolysis, biofilm formation, virulence, and capsular expression and is moreover a treatment target whose inhibition increases susceptibility to oxacillin (87). ArlR, which is highly expressed and then sharply declines upon drug exposure, has been shown to induce the expression of *mgrA* after it is phosphorylated. Here, however, the expression of mgrA rapidly drops in the first 2 h after antibiotic challenge and then recovers, suggesting alternative regulation of mgrA may also be taking place (88).

Other changes in transcriptional regulators suggest that entry into persistence is a response to overall cellular stress. The quaternary amine transport ATP-binding protein OpuCA (A0A0H2XHM3) encodes the ATP-binding cassette osmoprotectant uptake system OpuC (89) and is involved in osmotic stress adaptation. NirR, which is upregulated compared to control, is part of a more complex system that induces nitrite reductase expression under antibiotic stress. By sensing oxygen/nitrate and activating anaerobic respiration, it mitigates nitrosative damage and may support bacterial survival in hostile environments (90). More idiosyncratic changes were observed in other global regulators, including components of the *lyt* regulon that influences cell wall biosynthesis, *lysR*-type regulators that regulate metabolic adaptation during infection (91), but also MarR, which has previously been shown to aid antibiotic resistance in *S. aureus* and that regulates efflux systems that export antibiotics from the cell to the exterior (92, 93). Upregulation of MarR may facilitate antibiotic survival by decreasing the intracellular antibiotic concentration. Its decrease after antibiotic removal is consistent with the idea that bacteria are recovering from stress as they resume growth.

A final category of proteins that show extensive changes during the time course is those associated with prophages. Fluoroquinolones are known to induce prophages in different species (94), consistent with the idea that phages are responsive to cellular stress. Our sampling shows that several weakly expressed genes under control conditions, including a putative prophage integrase, become highly induced during antibiotic exposure, while others show the opposite pattern. Without further analysis of culture supernatants, it is difficult to determine the consequences of these changes for phage production and excision. However, it is tempting to hypothesize that these changes would lead to increased densities of free phage.

The time course and 4 h data reveal that many proteins are unnecessary during persistence, while others are actively associated with the global response to stress. The highly pleiotropic nature of many of the changes, especially to transcription factors, highlights that persistence is activated by numerous changes that modify whole-cell physiology, rather than a single (or few) persister targets. The results also uncover the value of the BONCAT approach to identify key proteins associated with persistence without the need for cell sorting of the persisters from the dead cell debris (95).

## DISCUSSION

To our knowledge, this study is the first example to use BONCAT to retrieve and quantify the persister proteome. Two strains of *S. aureus,* TCH1516 and 880, were challenged with a cell wall-disrupting antibiotic (oxacillin) or a gyrase inhibitor (moxifloxacin). The reason was to identify shared or unique mechanisms that regulate this phenotype under different types of antibiotic-induced stress. First, we confirmed that persisters are translationally active. The approach is highly complementary to the flow cytometry-based approach by Brul and co-workers (29–31) that makes use of two dyes to selectively label persister cells, and that could exclude the still-dividing part of the persister population (leading to CFSE-loss), and the cells that can quench and/or secrete the rather redox/pH-sensitive fluorescein dye on which the method is based.

Next, consistent with other observations, we found that upregulated and downregulated proteins in persisters are highly strain and antibiotic specific (9). While some of the antibiotic-specific changes are understandable based on the known mechanisms of the drug or the cellular response to these stresses, for example, upregulation of proteins associated with cell wall damage (*pbp2*) or β-lactam resistance (*mecA*) in the MRSA strains stressed with oxacillin, the larger fraction of protein changes is unique to either the strain or the drug (96–98). This result is consistent with phenotypic screens that identified significant variation between wild-type strains in the persister fraction during exposure to different antibiotics (99–101) and suggests that single diagnostic targets for persister cells may remain elusive. Finally, our results also suggest the value of a BONCAT approach in identifying proteins and pathways that may be causally involved in regulating this phenotype, serving as a starting point for further validation of the identified hits (11, 102). The depth and breadth of coverage during persistence allowed us to identify changes in expression of hundreds of known and unknown proteins, even when the fraction of persister cells within the larger population was extremely low. One thing that is important to keep in mind at all times with these experiments is that the lower rate of tRNA loading of BONCAT-amino acids can affect cellular metabolism (103). However, the fact that the BONCAT experiments still lead to similar protein expression (Fig. S2) suggests, in these experiments, that this did not include bias. These data therefore provide an exciting starting point to begin to further elucidate the mechanism(s) by which bacteria enter and exit persistence. Importantly, BONCAT had no effect on bacterial growth, ensuring that only antibiotic stress, and not “BONCAT-stress,” was responsible for the observed changes. One restriction of this approach is that fully translationally inactive subsets of the persister population, should they exist, would be missed by this method. Still, the identification of persister-specific protein changes makes clear that at least a part of the persister population is translationally active.

Mapping persister-associated proteins to universal databases such as Uniprot or KEGG highlights current limitations in our understanding of this complex phenotype, given the sizable fraction of changes that occurred in poorly studied or unannotated proteins, stressing the need for validation of the further study of these hits. Despite this, well-characterized proteins were recovered in our assays, including many that overlap with other studies of *S. aureus* persisters. Persisters of both strains under different antibiotic challenges had shared changes in proteins involved in maintaining cell wall integrity, with the joint expression of TCS to support cell-wall damage-related pathways (104, 105). This finding is consistent with results from other groups, where in response to cell wall targeting antibiotics *S. aureus* persisters increased cell wall biosynthesis-related proteins, such as those involved in the production of wall teichoic acids (*ltaS, tagA, dltD, msrR, A0A0H2XHQ0*) and peptidoglycan (*mgt, murP, uppS*) (29). We, and others, also observed upregulation of ABC transporters that may act to increase efflux to remove antibiotics from exposed cells (35, 106). In addition, we found changes in several pathways associated with regulating cellular metabolism and replication, including a decrease in proteins tied to anabolic pathways, in common with Liu et al. (13, 29). Changes to the stringent response via RelA/SpoT driven by ppGpp in response to nutrient limitation (e.g., carbon, amino acids, nitrogen, phosphate) were also seen. This response has been reported to trigger bacterial dormancy through downstream signaling of pathways involving toxin-antitoxin (TA) modules (107, 108), leading to a general shutdown of vital activities in response to stress (109).

The suite of responses we observed, and their alignment with changes in other species quantified using a broad range of techniques, lends credence to the BONCAT approach (110, 111). The method is easily and inexpensively applied and can be broadly used across species that vary in growth conditions or the factors that are reported to be important for persister induction. Our aims here were to validate the BONCAT approach in persister biology, so that new potential lead candidates. The further validation of these hits, particularly those that show high divergence between different strain-antibiotic combinations, offers an exciting opportunity to begin the elucidation of species-strain-specific persister mechanisms. This can, for example, be done using single gene knockout studies to directly evaluate the role of putative persister targets, as well as metabolic and systems modeling to understand whole-cell responses to the changes we have measured, both for known and uncharacterized proteins. In all, this deepened proteomic recovery of the persister population can, in combination with application to other species, hopefully aid in identifying new potential targets to overcome this limitation to effective antibiotic therapy.

## MATERIALS AND METHODS

### Safety statement

All biological experiments with *S. aureus* described in this study were performed under Bio Safety Level 2 conditions. Following fixation, further sample preparation for flow cytometry was performed under normal laboratory conditions.

### Bacterial strains and growth conditions

The bacterial culture strains were B013 (TCH1516) and B016 (880; BR-VRSA). Mid-exponential phase cultures were prepared by diluting overnight cultures 1:50 in Luria−Bertani (LB) broth and incubating these at 37°C at 100 rpm until the optical density (OD_600 nm_) had reached 0.4.

### Chemicals

All antibiotics were purchased from Sigma-Aldrich and used without further purification. Aha was purchased from Click Chemistry Tools. Neutravidin beads (Thermo Scientific Pierce, catalog number 20219)

### Minimum inhibitory concentration

MIC determination was done on a 96-well plate setup in biological triplicate. Mid-exponential phase cultures were prepared as above. Prior to the experiment, they were diluted to a starting inoculum size of 10^6^ CFU/mL. They were transferred to a 96-well plate containing Mueller-Hinton broth and antibiotic solution at desired concentrations to yield a final inoculum concentration of 5 × 10^5^ CFU/mL and incubated for the indicated time. The content of the wells was plated out on LB-Agar plates, which were incubated overnight at 37°C. The MIC was determined as the lowest concentration at which no visible bacterial growth was observed (56, 57).

### Time-kill assay

Mid-exponential phase cultures were prepared as above and were diluted to a starting inoculum of 10^6^ CFU/mL. They were then exposed to the antibiotics at a final concentration of 50× the MIC (62), and incubated at 37°C, 100 rpm. Samples at different times were taken, where necessary diluted in PBS (when too many colonies had formed for counting), performed, then plated on LB agar plates, and CFUs were counted after overnight incubation at 37°C (58).

### BONCAT labeling and lysis

Sample preparation was performed according to the procedure described in references (65, 66). BONCAT labeling of the persisters was done in the following way: bacteria were exposed to antibiotics in LB medium for the indicated time; after this, the bacteria were pelleted by centrifugation for 5 minutes at 3,000 *rcf*. The pellet was resuspended in SelenoMet medium (from Molecular Dimensions) augmented with the antibiotic at the same concentration to ensure that the bacteria remain under stress at all times. The samples were left on ice for 5′, following which they were centrifuged at 14,000 × *g* at 4°C for 10, prior to resuspension in fresh SelenoMet medium augmented with 4 mM Aha (112). The cells were incubated for 1.5 h, after which the cells were harvested by centrifugation and resuspended in lysis buffer (PBS, 4% SDS, with Roche EDTA-Free Protease Inhibitor added as per the manufacturer’s recommendation). The cells were lysed using a Bead Homogenizer (MP FastPrep-24 5G). The Bead Homogenizer was run for 10 cycles of 50 seconds each at 6 m/s, with 3 minute intervals on ice in between cycles. Following homogenization, tubes were centrifuged at 1,500 *rcf* for 2 minutes and supernatant was transferred to clean tubes. These were centrifuged again for 30 minutes at 4°C at 14,000 rpm, sterile-filtered through 0.2 µm membranes. Next, the reduction-alkylation was performed by incubation with DTT (1M) added per 1 mL sample, incubated for 15 minutes at 65°C, 600 rpm. Next, 80 µL of IAA stock (0.5M) was added and incubated for 30 minutes at room temperature in darkness. Next, 350 µL of SDS stock (10%) was added and incubated for 5 minutes at 65°C. Next, samples followed Methanol/Chloroform precipitation (113). BCA assay was performed according to the manufacturer’s protocol to determine the protein concentration.

### Analysis of BONCAT labeling by flow cytometry

*S. aureus* was metabolically labeled as described above and samples with OD_600_ ≈ 0.4 were collected after 30 minutes, 1 h and 2 h to analyze the label incorporation levels at single-bacterium level by flow cytometry. Bacterial samples were pelleted by centrifugation (10 min at 5,000 *rcf*), washed once with PBS, and resuspended in 100 µL 4% PFA for overnight fixation. 100,000 events were measured per condition. Fixed bacteria were washed once with 100 µL cold PBS and centrifuged 10 minutes at 6,000 *rcf,* then permeabilized in 50 µL permeabilization buffer (0.1% Triton-X100 in PBS) for 20 minutes. Permeabilized bacteria were washed once with 100 µL PBS and incubated with 50 µL of click mix (1 mM CuSO4, 10 mM sodium ascorbate, 1 mM THPTA ligand, 10 mM aminoguanidine, 96 mM HEPES, 5 µM AF488-alkyne, and pH 7.4) for 1 h in the dark at RT (table). After incubation, cells were washed once with 100 µL PBS and resuspended in 100 µL washing buffer (1% BSA in PBS) and incubated for 30 minutes in the dark at RT. After incubation, cells were washed once with 100 µL FACS buffer (EDTA 2 mM in PBS) and resuspended in 200 µL FACS buffer, then flow cytometry analysis was performed. Aha-AF488 was detected in the FITC channel. The analysis was performed using the Guava InCyte software, and all subsequent analyses were performed with FlowJo V10.7.2 (FlowJo software). The measured events were gated on size, shape, and fluorescence to accurately select single bacteria.

### Fluorescence SDS-PAGE analysis

20 µg of protein was diluted to a final volume of 10 µL with HEPES buffer (100 mM pH 7.4). 5 µL of click buffer (3 mM CuSO4, 30 mM sodium ascorbate, 3 mM THPTA ligand, 30 mM aminoguanidine, 300 mM HEPES, 15 µM AF488-alkyne, pH 7.4) was added, and the mixture was incubated for 1 h at RT in the dark. Sample buffer without thiol was added, the sample was heated to 95°C for 10 minutes, followed by SDS-PAGE separation. Prior to Coomassie staining, the gels were imaged in a ChemiDoc fluorescent gel scanner with the 700/50, 602/50, or 532/28 nm filter set.

### Bioorthogonal pull-down

Sample preparation was performed according to the procedure described in reference (77). 300 µg of protein was volume adjusted to 2 mg/mL (150 µL). An equal volume of double-concentrate click mix was added (2 mM CuSO4, 20 mM sodium ascorbate, 2 mM THPTA ligand, 20 mM aminoguanidine, 200 mM HEPES, 160 µM Biotin-PEG-Alkyne, and pH 7.4), and the mixture was reacted for 2 h at RT in the dark under gentle rotation.

Excess unreacted biotin-PEG-alkyne tag was removed by precipitating out the proteins with chloroform/methanol. First, 200 µL of 50 mM Hepes (100 mM, pH 7.4) was added, followed by 666 µL methanol. After vigorous vortexing, 166 µL chloroform was added, followed by a further burst of vortexing. 150 µL of water was added to the mixture to cause phase separation. Centrifugation for 10 min at 10,000 *rcf* at room temperature yielded a three-layer system where the top layer was water/methanol, the white film at the interface was the precipitated protein, and the bottom phase was the chloroform/methanol. The top layer was removed carefully, 600 µL methanol was added and mixed gently, followed by centrifugation for 10 min at 10,000 *rcf* and RT. After this, the supernatant was removed and the pellet dried for <2 min in air. The pellet was resuspended in 250 µL urea buffer (8 M urea in 25 mM ammonium bicarbonate, pH 8.0).

Next, 100 µL of Neutravidin beads (Thermo Scientific Pierce, catalog number 20219) were washed three times with 250 µL PBS and resuspended in 2 mL PBS, the proteins were added on top and incubated for 3 hours at room temperature under gentle rotation. After which, the beads were collected by centrifugation (2 minutes at 2,500 *rcf*) and washed 5 times with 2 mL PBS with vigorous shaking, followed by centrifugation to remove any SDS. After the final wash, the supernatant was removed and 250 µL of on-bead digestion buffer (100 mM Tris pH 8.0, 100 mM NaCl, 1 mM CaCl2, and 2% ACN) was added to each of the bead residues, the beads were transferred to low-binding tubes (1.5 mL, Sarstedt). Each sample was treated with 1 µL of trypsin solution (0.5 µg/µL Sequencing Grade Modified Trypsin, Porcine (Promega) in 0.1 mM HCl), and the samples were incubated at 37°C overnight while shaking (950 rpm).

To each sample, formic acid (3 µL) was added, followed by filtering off the beads over biospin columns (Bio-Rad, 7326207) on top of 2 mL Eppendorf tubes using centrifugation (2 min, 300 *rcf*). Note: Now, the 2 mL Eppendorf tubes contain the main sample solution. Next, StageTips were used for subsequent desalting of the samples according to the procedure described in reference (114). StageTips were placed in holders in Eppendorf tubes to collect the flow after each step, which is followed by centrifugation (2 min, 300 *rcf*). The conditioning of the StageTips started with 50 µL MeOH, washing with 50 µL solution B (80% acetonitrile vol/vol, 0.5% vol/vol formic acid in water), and 50 µL solution A (0.5% vol/vol formic acid in water). The sample solution was loaded through the StageTips, followed by a wash with 50 µL solution A. The StageTips were then transferred to low-binding tubes, and the tips were flushed with 100 µL solution B. The collected sample was concentrated in a SpeedVac (45°C, V-AQ, 1–2 h) (Eppendorf Concentrator 5301). The samples were stored at 20°C until LC-MS measurement.

### LC-MS measurement and analysis

Samples were reconstituted in 50 µL LC-MS sample solution (3% vol/vol acetonitrile, 0.1% vol/vol formic acid in Milli-Q) to follow the Nanodrop measurement. Samples were diluted to 100 ng/µL in LC-MS sample solution containing 10 fmol/µL yeast enolase digest (cat. 186002325, Waters). The injection amount was titrated using a pooled quality control sample to prevent overloading the nanoLC system and the automatic gain control (AGC) of the QExactive mass spectrometer. The desalted peptides were separated on an UltiMate 3000 RSLCnano system set in a trap-elute configuration with a nanoEase M/Z Symmetry C18 100 Å, 5 µm, 180 µm × 20 mm trap column (Waters) for peptide loading/retention and nanoEase M/Z HSS C18 T3 100 Å, 1.8 µm, 75 µm × 250 mm analytical column (Waters) for peptide separation both kept at 40°C in a column oven. Samples were injected on the trap column at a flow rate of 15 µL/min for 2 min with 99% mobile phase A (0.1% FA in ULC-MS grade water [Biosolve]), 1% mobile phase B (0.1% FA in ULC-MS grade acetonitrile [Biosolve]) eluent. The 85 min LC method, using mobile phase A and mobile phase B controlled by a flow sensor at 0.3 µL/min with average pressure of 400–500 bar (5,500–7,000 psi), was programmed as gradient with linear increment to 1% B from 0 to 2 min, 5% B at 5 min, 22% B at 55 min, 40% B at 64 min, 90% B at 65–74 min, and 1% B at 75–85 min. The eluent was introduced by electro-spray ionization (ESI) via the nanoESI source (Thermo) using stainless steel Nano-bore emitters (40 mm, OD 1/32″, ES542, Thermo Scientific).

The QExactive HF was operated in positive mode with data-dependent acquisition (no lock mass), default charge of 2 + and external calibration with LTQ Velos ESI-positive ion calibration solution (88323, Pierce, Thermo) every 5 days to >2 ppm. The tune file for the survey scan was set to scan range of 350–1,400 m/z, 120,000 resolution (m/z 200), 1 microscan, automatic gain control (AGC) of 3e6, max injection time of 100 ms, no sheath, aux or sweep gas, spray voltage ranging from 1.7 to 3.0 kV, capillary temp of 250°C, and an S-lens value of 80 V. For the 10 data-dependent MS/MS events, the loop count was set to 10 and the general settings were resolution to 15,000, AGC target 1e5, max IT time 50 ms, isolation window of 1.8 m/z, fixed first mass of 120 m/z, and normalized collision energy (NCE) of 28 eV. For individual peaks, the data-dependent settings were 1.00e3 for the minimum AGC target yielding an intensity threshold of 2.0e4 that needs to be reached prior to triggering an MS/MS event. No apex trigger was used, unassigned, +1, and charges >+ 8 were excluded with peptide match mode preferred, isotope exclusion on, and dynamic exclusion of 10 s. In between experiments, routine wash and control runs were done by injecting 5 µL LC-MS solution containing 5 µL of 10 fmol/µL BSA or enolase digest and 1 µL of 10 fmol/µL angiotensin III (Fluka, Thermo)/oxytocin (Merck) to check the performance of the platform on each component (nano-LC, the mass spectrometer [mass calibration/quality of ion selection and fragmentation] and the search engine).

### MS data analysis

Raw files from LC-MS measurement were analyzed using the MaxQuant software (version 1.6.17.0) with Andromeda search engine (115). The settings applied for the analysis were as follows: fixed modification: carbamidomethylation (cysteine); variable modification: oxidation (methionine), acetylation (N-terminus); proteolytic enzyme: trypsin/P; missed cleavages: 2; main search tolerance: 4.5 ppm; false discovery rates: 0.01. The options “LFQ” and “match between runs” were checked, while “second peptides” was unchecked. Searches were performed against the UniProt database FASTA file for the *S. aureus* USA300 proteome (Uniprot ID: UP000000793, downloaded 05-03-2023). The data were extracted from “peptides.txt” and “proteingroups.txt” files to obtain protein coverage and MaxQuant scores and for Perseus analysis.

The first analysis of the MaxQuant output was performed using Perseus (version 1.6.15.0). The protein group txt file was loaded on MaxQuant, then LFQ intensity entries were selected to the main section. The data matrix loaded was filtered by applying filter rows followed by filter rows based on categorical column and only identified by site option, the resulting matrix was filtered again by applying filter rows followed by filter rows based on categorical column and reverse option, the resulting matrix was filtered again by applying filter rows followed by filter rows based on categorical column and potential contaminant option. Next, the categorization of the different files in groups according to experimental design was performed by annotation rows and the categorical annotation rows option. In this section, all the samples for the same condition were labeled under the same name. The resulting data were transformed into log2 values by choosing basic and transform options. The matrix was filtered by filter rows followed by filter rows based on valid values. To solve the problem of missing values, data imputation was performed with a replacement from a normal distribution with width: 0.3 and a down shift: 1.8. Normalization was performed by subtraction and a change from rows to columns with the subtraction of the most frequent value. With the resultant matrix, volcano plots were performed with the first group as the problem sample and the second group as the control sample. A t-test with an FDR threshold of 0.05 is applied to create the volcano plot. The −log *P* value and difference cut off was set at +2.

The hits above the threshold were obtained from the matrix derived from the volcano plot, and the data were used for comparison against the UniProt protein database for protein annotation and pathway allocation. The selection of proteins with associated pathways was done using Excel. Venn diagrams were generated using the R studio package (7-64), loading the data tables with hits above threshold and the reported pathways previously obtained after Uniprot comparison and Excel processing. The database for the heatmaps was the result of the additional analysis on Perseus from the database derived from the volcano plots, applying a multiple sample test ANOVA, the matrix was filtered based on categorical column according to ANOVA significant values with the mode selection on keeping matching rows, then a normalization based on Z-score based on rows, then a second normalization based on Z-score based on columns, the matrix derived was loaded in Excel to average the technical replicates and biological replicates for the same condition and then loaded in R to obtain the heatmaps.

## SUPPLEMENTAL MATERIAL

Supplemental material msphere.00431-25-s0001.docx Supplemental figures and Tables S1 to S5.

Supplemental tables msphere.00431-25-s0002.xlsx Tables S6 to S14.

ASM does not own the copyrights to Supplemental Material that may be linked to, or accessed through, an article. The authors have granted ASM a non-exclusive, world-wide license to publish the Supplemental Material files. Please contact the corresponding author directly for reuse.

## References

[1] Balaban NQ, Helaine S, Lewis K, Ackermann M, Aldridge B, Andersson DI, Brynildsen MP, Bumann D, Camilli A, Collins JJ, Dehio C, Fortune S, Ghigo J-M, Hardt W-D, Harms A, Heinemann M, Hung DT, Jenal U, Levin BR, Michiels J, Storz G, Tan M-W, Tenson T, Van Melderen L, Zinkernagel A. 2019. Definitions and guidelines for research on antibiotic persistence. Nat Rev Microbiol 17:441–448. doi:10.1038/s41579-019-0196-3 30980069 PMC7136161

[2] Levin-Reisman I, Ronin I, Gefen O, Braniss I, Shoresh N, Balaban NQ. 2017. Antibiotic tolerance facilitates the evolution of resistance. Science 355:826–830. doi:10.1126/science.aaj2191 28183996

[3] Wuyts J, Van Dijck P, Holtappels M. 2018. Fungal persister cells: the basis for recalcitrant infections? PLOS Pathog 14:e1007301. doi:10.1371/journal.ppat.1007301 30335865 PMC6193731

[4] Wakamoto Y, Dhar N, Chait R, Schneider K, Signorino-Gelo F, Leibler S, McKinney JD. 2013. Dynamic persistence of antibiotic-stressed mycobacteria. Science 339:91–95. doi:10.1126/science.1229858 23288538

[5] Shan Y, Brown Gandt A, Rowe SE, Deisinger JP, Conlon BP, Lewis K. 2017. ATP-dependent persister formation in Escherichia coli*.* mBio 8:e02267-16. doi:10.1128/mBio.02267-16 28174313 PMC5296605

[6] Mulcahy LR, Burns JL, Lory S, Lewis K. 2010. Emergence of Pseudomonas aeruginosa strains producing high levels of persister cells in patients with cystic fibrosis. J Bacteriol 192:6191–6199. doi:10.1128/JB.01651-09 20935098 PMC2981199

[7] Helaine S, Cheverton AM, Watson KG, Faure LM, Matthews SA, Holden DW. 2014. Internalization of Salmonella by macrophages induces formation of nonreplicating persisters. Science 343:204–208. doi:10.1126/science.1244705 24408438 PMC6485627

[8] Conlon BP, Rowe SE, Gandt AB, Nuxoll AS, Donegan NP, Zalis EA, Clair G, Adkins JN, Cheung AL, Lewis K. 2016. Persister formation in Staphylococcus aureus is associated with ATP depletion. Nat Microbiol 1:16051. doi:10.1038/nmicrobiol.2016.51 PMC4932909 27572649

[9] Niu H, Gu J, Zhang Y. 2024. Bacterial persisters: molecular mechanisms and therapeutic development. Signal Transduct Target Ther 9:174. doi:10.1038/s41392-024-01866-5 39013893 PMC11252167

[10] Van den Bergh B, Fauvart M, Michiels J. 2017. Formation, physiology, ecology, evolution and clinical importance of bacterial persisters. FEMS Microbiol Rev 41:219–251. doi:10.1093/femsre/fux001 28333307

[11] Sulaiman JE, Lam H. 2019. Application of proteomics in studying bacterial persistence. Expert Rev Proteomics 16:227–239. doi:10.1080/14789450.2019.1575207 30681894

[12] Louwagie E, Verstraete L, Michiels J, Verstraeten N. 2021. Studying bacterial persistence: established methods and current advances. Methods Mol Biol 2357:3–20. doi:10.1007/978-1-0716-1621-5_1 34590248

[13] Liu S, Huang Y, Jensen S, Laman P, Kramer G, Zaat SAJ, Brul S. 2024. Molecular physiological characterization of the dynamics of persister formation in Staphylococcus aureus. Antimicrob Agents Chemother 68:e0085023. doi:10.1128/aac.00850-23 38051079 PMC10777834

[14] Orman MA, Brynildsen MP. 2013. Dormancy is not necessary or sufficient for bacterial persistence. Antimicrob Agents Chemother 57:3230–3239. doi:10.1128/AAC.00243-13 23629720 PMC3697331

[15] Spanka D-T, Konzer A, Edelmann D, Berghoff BA. 2019. High-throughput proteomics identifies proteins with importance to postantibiotic recovery in depolarized persister cells. Front Microbiol 10:378. doi:10.3389/fmicb.2019.00378 30894840 PMC6414554

[16] Manuse S, Shan Y, Canas-Duarte SJ, Bakshi S, Sun W-S, Mori H, Paulsson J, Lewis K. 2021. Bacterial persisters are a stochastically formed subpopulation of low-energy cells. PLOS Biol 19:e3001194. doi:10.1371/journal.pbio.3001194 33872303 PMC8084331

[17] Balaban NQ, Merrin J, Chait R, Kowalik L, Leibler S. 2004. Bacterial persistence as a phenotypic switch. Science 305:1622–1625. doi:10.1126/science.1099390 15308767

[18] Cameron DR, Shan Y, Zalis EA, Isabella V, Lewis K. 2018. A genetic determinant of persister cell formation in bacterial pathogens. J Bacteriol 200:e00303-18. doi:10.1128/JB.00303-18 29941425 PMC6088157

[19] Molina-Quiroz RC, Lazinski DW, Camilli A, Levy SB. 2016. Transposon-sequencing analysis unveils novel genes involved in the generation of persister cells in uropathogenic Escherichia coli*.* Antimicrob Agents Chemother 60:6907–6910. doi:10.1128/AAC.01617-16 27550350 PMC5075105

[20] Tong SYC, Davis JS, Eichenberger E, Holland TL, Fowler VG Jr. 2015. Staphylococcus aureus infections: epidemiology, pathophysiology, clinical manifestations, and management. Clin Microbiol Rev 28:603–661. doi:10.1128/CMR.00134-14 26016486 PMC4451395

[21] Cheung GYC, Bae JS, Otto M. 2021. Pathogenicity and virulence of Staphylococcus aureus. Virulence 12:547–569. doi:10.1080/21505594.2021.1878688 33522395 PMC7872022

[22] Howden BP, Giulieri SG, Wong Fok Lung T, Baines SL, Sharkey LK, Lee JYH, Hachani A, Monk IR, Stinear TP. 2023. Staphylococcus aureus host interactions and adaptation. Nat Rev Microbiol 21:380–395. doi:10.1038/s41579-023-00852-y 36707725 PMC9882747

[23] Pan X, Liu W, Du Q, Zhang H, Han D. 2023. Recent advances in bacterial persistence mechanisms. Int J Mol Sci 24:14311. doi:10.3390/ijms241814311 37762613 PMC10531727

[24] Geiger T, Goerke C, Fritz M, Schäfer T, Ohlsen K, Liebeke M, Lalk M, Wolz C. 2010. Role of the (p)ppGpp synthase RSH, a RelA/SpoT homolog, in stringent response and virulence of Staphylococcus aureus. Infect Immun 78:1873–1883. doi:10.1128/IAI.01439-09 20212088 PMC2863498

[25] Fernandes PB, Reed P, Monteiro JM, Pinho MG. 2022. Revisiting the role of VraTSR in Staphylococcus aureus response to cell wall-targeting antibiotics. J Bacteriol 204:e0016222. doi:10.1128/jb.00162-22 35862765 PMC9380581

[26] Peng Q, Guo L, Dong Y, Bao T, Wang H, Xu T, Zhang Y, Han J. 2022. PurN is involved in antibiotic tolerance and virulence in Staphylococcus aureus. Antibiotics (Basel) 11:12. doi:10.3390/antibiotics11121702 PMC9774800 36551359

[27] Pandey S, Sahukhal GS, Elasri MO. 2021. The msaABCR operon regulates persister formation by modulating energy metabolism in Staphylococcus aureus. Front Microbiol 12:657753. doi:10.3389/fmicb.2021.657753 33936014 PMC8079656

[28] Personnic N, Striednig B, Lezan E, Manske C, Welin A, Schmidt A, Hilbi H. 2019. Quorum sensing modulates the formation of virulent Legionella persisters within infected cells. Nat Commun 10:5216. doi:10.1038/s41467-019-13021-8 31740681 PMC6861284

[29] Liu Shiqi, Laman P, Jensen S, van der Wel NN, Kramer G, Zaat SAJ, Brul S. 2024. Isolation and characterization of persisters of the pathogenic microorganism Staphylococcus aureus. iScience 27:110002. doi:10.1016/j.isci.2024.110002 38868179 PMC11166702

[30] Liu S, Brul S, Zaat SAJ. 2021. Isolation of persister cells of Bacillus subtilis and determination of their susceptibility to antimicrobial peptides. IJMS 22:10059. doi:10.3390/ijms221810059 34576222 PMC8470456

[31] Liu S. 2024. Molecular physiological characterization of the dynamics of persister formation in Staphylococcus aureus. Antimicrob Agents Chemother (Bethesda) 68:e00850–23. doi:10.1128/aac.00850-23 PMC10777834 38051079

[32] Scinto SL, Bilodeau DA, Hincapie R, Lee W, Nguyen SS, Xu M, Am Ende CW, Finn MG, Lang K, Lin Q, Pezacki JP, Prescher JA, Robillard MS, Fox JM. 2021. Bioorthogonal chemistry. Nat Rev Methods Primers 1:30. doi:10.1038/s43586-021-00028-z 34585143 PMC8469592

[33] White BM, Kumar P, Conwell AN, Wu K, Baskin JM. 2022. Lipid expansion microscopy. J Am Chem Soc 144:18212–18217. doi:10.1021/jacs.2c03743 36190998 PMC9727412

[34] Shanbhag K, Sharma K, Kamat SS. 2023. Photoreactive bioorthogonal lipid probes and their applications in mammalian biology. RSC Chem Biol 4:37–46. doi:10.1039/d2cb00174h 36685253 PMC9811504

[35] Banahene N, Kavunja HW, Swarts BM. 2022. Chemical reporters for bacterial glycans: development and applications. Chem Rev 122:3336–3413. doi:10.1021/acs.chemrev.1c00729 34905344 PMC8958928

[36] Fantoni NZ, El-Sagheer AH, Brown T. 2021. A hitchhiker’s guide to click-chemistry with nucleic acids. Chem Rev 121:7122–7154. doi:10.1021/acs.chemrev.0c00928 33443411

[37] Smriga S, Samo T, Malfatti F, Villareal J, Azam F. 2014. Individual cell DNA synthesis within natural marine bacterial assemblages as detected by “click” chemistry. Aquat Microb Ecol 72:269–280. doi:10.3354/ame01698

[38] Bird RE, Lemmel SA, Yu X, Zhou QA. 2021. Bioorthogonal chemistry and its applications. Bioconjug Chem 32:2457–2479. doi:10.1021/acs.bioconjchem.1c00461 34846126

[39] Pelgrom LR, Davis GM, O’Shaughnessy S, Wezenberg EJM, Van Kasteren SI, Finlay DK, Sinclair LV. 2023. QUAS-R: an SLC1A5-mediated glutamine uptake assay with single-cell resolution reveals metabolic heterogeneity with immune populations. Cell Rep 42:112828. doi:10.1016/j.celrep.2023.112828 37478011

[40] Bertheussen K, van de Plassche M, Bakkum T, Gagestein B, Ttofi I, Sarris AJC, Overkleeft HS, van der Stelt M, van Kasteren SI. 2022. Live-cell imaging of sterculic acid-a naturally occurring 1,2-cyclopropene fatty acid-by bioorthogonal reaction with turn-on tetrazine-fluorophore conjugates. Angew Chem Int Ed Engl 61:e202207640. doi:10.1002/anie.202207640 35838324 PMC9546306

[41] Hatzenpichler R, Scheller S, Tavormina PL, Babin BM, Tirrell DA, Orphan VJ. 2014. In situ visualization of newly synthesized proteins in environmental microbes using amino acid tagging and click chemistry. Environ Microbiol 16:2568–2590. doi:10.1111/1462-2920.12436 24571640 PMC4122687

[42] Dieterich DC, Link AJ, Graumann J, Tirrell DA, Schuman EM. 2006. Selective identification of newly synthesized proteins in mammalian cells using bioorthogonal noncanonical amino acid tagging (BONCAT). Proc Natl Acad Sci USA 103:9482–9487. doi:10.1073/pnas.0601637103 16769897 PMC1480433

[43] Kiick KL, Saxon E, Tirrell DA, Bertozzi CR. 2002. Incorporation of azides into recombinant proteins for chemoselective modification by the staudinger ligation. Proc Natl Acad Sci USA 99:19–24. doi:10.1073/pnas.012583299 11752401 PMC117506

[44] van Eldijk MB, van Hest JCM. 2018. Residue-specific incorporation of noncanonical amino acids for protein engineering. Methods Mol Biol 1728:137–145. doi:10.1007/978-1-4939-7574-7_8 29404995

[45] Ngo JT, Tirrell DA. 2011. Noncanonical amino acids in the interrogation of cellular protein synthesis. Acc Chem Res 44:677–685. doi:10.1021/ar200144y 21815659 PMC3178009

[46] Bakkum T, van Leeuwen T, Sarris AJC, van Elsland DM, Poulcharidis D, Overkleeft HS, van Kasteren SI. 2018. Quantification of bioorthogonal stability in immune phagocytes using flow cytometry reveals rapid degradation of strained alkynes. ACS Chem Biol 13:1173–1179. doi:10.1021/acschembio.8b00355 29693370 PMC5962927

[47] Tian H, Sakmar TP, Huber T. 2016. A simple method for enhancing the bioorthogonality of cyclooctyne reagent. Chem Commun (Camb) 52:5451–5454. doi:10.1039/c6cc01321j 27009873 PMC4824645

[48] van Kasteren S, Rozen DE. 2023. Using click chemistry to study microbial ecology and evolution. ISME Commun 3:9. doi:10.1038/s43705-022-00205-5 36721064 PMC9889756

[49] Ignacio BJ, Dijkstra J, Mora N, Slot EFJ, van Weijsten MJ, Storkebaum E, Vermeulen M, Bonger KM. 2023. THRONCAT: metabolic labeling of newly synthesized proteins using a bioorthogonal threonine analog. Nat Commun 14:3367. doi:10.1038/s41467-023-39063-7 37291115 PMC10250548

[50] Schoch CL, Ciufo S, Domrachev M, Hotton CL, Kannan S, Khovanskaya R, Leipe D, Mcveigh R, O’Neill K, Robbertse B, Sharma S, Soussov V, Sullivan JP, Sun L, Turner S, Karsch-Mizrachi I. 2020. NCBI Taxonomy: a comprehensive update on curation, resources and tools. Database (Oxford) 2020:baaa062. doi:10.1093/database/baaa062 32761142 PMC7408187

[51] Highlander SK, Hultén KG, Qin X, Jiang H, Yerrapragada S, Mason EO Jr, Shang Y, Williams TM, Fortunov RM, Liu Y, et al.. 2007. Subtle genetic changes enhance virulence of methicillin resistant and sensitive Staphylococcus aureus*.* BMC Microbiol 7:99. doi:10.1186/1471-2180-7-99 17986343 PMC2222628

[52] Dien Bard J, Hindler JA, Gold HS, Limbago B. 2014. Rationale for eliminating Staphylococcus breakpoints for β-lactam agents other than penicillin, oxacillin or cefoxitin, and ceftaroline. Clin Infect Dis 58:1287–1296. doi:10.1093/cid/ciu043 24457339 PMC5734619

[53] Humma ZE, Patel P. 2024. Moxifloxacin. In StatPearls. StatPearls Publishing LLC.: Treasure Island (FL). 38261682

[54] Poudel S, Tsunemoto H, Meehan M, Szubin R, Olson CA, Lamsa A, Seif Y, Dillon N, Vrbanac A, Sugie J, Dahesh S, Monk JM, Dorrestein PC, Pogliano J, Knight R, Nizet V, Palsson BO, Feist AM. 2019. Characterization of CA-MRSA TCH1516 exposed to nafcillin in bacteriological and physiological media. Sci Data 6:43. doi:10.1038/s41597-019-0051-4 31028276 PMC6486602

[55] Cutrona N, Gillard K, Ulrich R, Seemann M, Miller HB, Blackledge MS. 2019. From antihistamine to anti-infective: loratadine inhibition of regulatory PASTA kinases in staphylococci reduces biofilm formation and potentiates β-lactam antibiotics and vancomycin in resistant strains of Staphylococcus aureus. ACS Infect Dis 5:1397–1410. doi:10.1021/acsinfecdis.9b00096 31132246

[56] Wiegand I, Hilpert K, Hancock REW. 2008. Agar and broth dilution methods to determine the minimal inhibitory concentration (MIC) of antimicrobial substances. Nat Protoc 3:163–175. doi:10.1038/nprot.2007.521 18274517

[57] Waites KB. 2011. CLSI standards: guidelines for health care excellence. In Methods for antimicrobial susceptibility testing for human mycoplasmas. Clinical and Laboratory Standards Institute: Wayne (PA). 31339681

[58] Balouiri M, Sadiki M, Ibnsouda SK. 2016. Methods for in vitro evaluating antimicrobial activity: a review. J Pharm Anal 6:71–79. doi:10.1016/j.jpha.2015.11.005 29403965 PMC5762448

[59] Parsons JB, Westgeest AC, Conlon BP, Fowler VG Jr. 2023. Persistent methicillin-resistant Staphylococcus aureus bacteremia: host, pathogen, and treatment. Antibiotics (Basel) 12:455. doi:10.3390/antibiotics12030455 36978320 PMC10044482

[60] Huemer M, Mairpady Shambat S, Brugger SD, Zinkernagel AS. 2020. Antibiotic resistance and persistence-Implications for human health and treatment perspectives. EMBO Rep 21:e51034. doi:10.15252/embr.202051034 33400359 PMC7726816

[61] Nguyen TK, Peyrusson F, Dodémont M, Pham NH, Nguyen HA, Tulkens PM, Van Bambeke F. 2020. The persister character of clinical isolates of Staphylococcus aureus contributes to faster evolution to resistance and higher survival in THP-1 monocytes: a study with moxifloxacin. Front Microbiol 11:587364. doi:10.3389/fmicb.2020.587364 33329458 PMC7719683

[62] Peyrusson F, Varet H, Nguyen TK, Legendre R, Sismeiro O, Coppée J-Y, Wolz C, Tenson T, Van Bambeke F. 2020. Intracellular Staphylococcus aureus persisters upon antibiotic exposure. Nat Commun 11:2200. doi:10.1038/s41467-020-15966-7 32366839 PMC7198484

[63] Brauner A, Fridman O, Gefen O, Balaban NQ. 2016. Distinguishing between resistance, tolerance and persistence to antibiotic treatment. Nat Rev Microbiol 14:320–330. doi:10.1038/nrmicro.2016.34 27080241

[64] van Elsland DM, Bos E, de Boer W, Overkleeft HS, Koster AJ, van Kasteren SI. 2016. Detection of bioorthogonal groups by correlative light and electron microscopy allows imaging of degraded bacteria in phagocytes. Chem Sci 7:752–758. doi:10.1039/c5sc02905h 28791116 PMC5529995

[65] van Elsland DM, Pujals S, Bakkum T, Bos E, Oikonomeas-Koppasis N, Berlin I, Neefjes J, Meijer AH, Koster AJ, Albertazzi L, van Kasteren SI. 2018. Ultrastructural Imaging of Salmonella-host interactions using super-resolution correlative light-electron microscopy of bioorthogonal pathogens. Chembiochem 19:1766–1770. doi:10.1002/cbic.201800230 29869826 PMC6120560

[66] Bakkum T, Heemskerk MT, Bos E, Groenewold M, Oikonomeas-Koppasis N, Walburg KV, van Veen S, van der Lienden MJC, van Leeuwen T, Haks MC, Ottenhoff THM, Koster AJ, van Kasteren SI. 2020. Bioorthogonal correlative light-electron microscopy of Mycobacterium tuberculosis in macrophages reveals the effect of antituberculosis drugs on subcellular bacterial distribution. ACS Cent Sci 6:1997–2007. doi:10.1021/acscentsci.0c00539 33274277 PMC7706097

[67] Meldal M, Tornøe CW. 2008. Cu-catalyzed azide-alkyne cycloaddition. Chem Rev 108:2952–3015. doi:10.1021/cr0783479 18698735

[68] Lin W, Li R, Cao S, Li H, Yang K, Yang Z, Su J, Zhu Y-G, Cui L. 2024. High-throughput single-cell metabolic labeling, sorting, and sequencing of active antibiotic-resistant bacteria in the environment. Environ Sci Technol 58:17838–17849. doi:10.1021/acs.est.4c02906 39333059

[69] Lindivat M, Larsen A, Hess-Erga OK, Bratbak G, Hoell IA. 2020. Bioorthogonal non-canonical amino acid tagging combined with flow cytometry for determination of activity in aquatic microorganisms. Front Microbiol 11:1929. doi:10.3389/fmicb.2020.01929 33013733 PMC7461810

[70] Link AJ, Vink MKS, Agard NJ, Prescher JA, Bertozzi CR, Tirrell DA. 2006. Discovery of aminoacyl-tRNA synthetase activity through cell-surface display of noncanonical amino acids. Proc Natl Acad Sci USA 103:10180–10185. doi:10.1073/pnas.0601167103 16801548 PMC1502431

[71] van Hest JCM, Tirrell DA. 2001. Protein-based materials, toward a new level of structural control. Chem Commun 2001:1897–1904. doi:10.1039/b105185g 12240211

[72] Reichart NJ, Jay ZJ, Krukenberg V, Parker AE, Spietz RL, Hatzenpichler R. 2020. Activity-based cell sorting reveals responses of uncultured archaea and bacteria to substrate amendment. ISME J 14:2851–2861. doi:10.1038/s41396-020-00749-1 32887944 PMC7784905

[73] Schiapparelli LM, McClatchy DB, Liu H-H, Sharma P, Yates JR 3rd, Cline HT. 2014. Direct detection of biotinylated proteins by mass spectrometry. J Proteome Res 13:3966–3978. doi:10.1021/pr5002862 25117199 PMC4156236

[74] Kleinpenning F, Steigenberger B, Wu W, Heck AJR. 2020. Fishing for newly synthesized proteins with phosphonate-handles. Nat Commun 11:3244. doi:10.1038/s41467-020-17010-0 32591520 PMC7320153

[75] Hibbert JE, Jorgenson KW, Zhu WG, Steinert ND, Hornberger TA. 2023. Protocol for quantifying the in vivo rate of protein degradation in mice using a pulse-chase technique. STAR Protoc 4:102574. doi:10.1016/j.xpro.2023.102574 37729055 PMC10517276

[76] van der Wouden PE, Zwinderman MRH, Chen D, Borghesan M, Dekker FJ. 2023. Protocol of the double-click seq method to evaluate the deposition bias of chromatin proteins. Curr Protoc 3:e805. doi:10.1002/cpz1.805 37338240

[77] van Rooden EJ, Florea BI, Deng H, Baggelaar MP, van Esbroeck ACM, Zhou J, Overkleeft HS, van der Stelt M. 2018. Mapping in vivo target interaction profiles of covalent inhibitors using chemical proteomics with label-free quantification. Nat Protoc 13:752–767. doi:10.1038/nprot.2017.159 29565900

[78] Prax M, Bertram R. 2014. Metabolic aspects of bacterial persisters. Front Cell Infect Microbiol 4:148. doi:10.3389/fcimb.2014.00148 25374846 PMC4205924

[79] Orman MA, Brynildsen MP. 2013. Establishment of a method to rapidly assay bacterial persister metabolism. Antimicrob Agents Chemother 57:4398–4409. doi:10.1128/AAC.00372-13 23817376 PMC3754326

[80] Radzikowski JL, Vedelaar S, Siegel D, Ortega ÁD, Schmidt A, Heinemann M. 2016. Bacterial persistence is an active σS stress response to metabolic flux limitation. Mol Syst Biol 12:882. doi:10.15252/msb.20166998 27655400 PMC5043093

[81] Radzikowski JL, Schramke H, Heinemann M. 2017. Bacterial persistence from a system-level perspective. Curr Opin Biotechnol 46:98–105. doi:10.1016/j.copbio.2017.02.012 28292710

[82] Huemer M, Mairpady Shambat S, Bergada-Pijuan J, Söderholm S, Boumasmoud M, Vulin C, Gómez-Mejia A, Antelo Varela M, Tripathi V, Götschi S, Marques Maggio E, Hasse B, Brugger SD, Bumann D, Schuepbach RA, Zinkernagel AS. 2021. Molecular reprogramming and phenotype switching in Staphylococcus aureus lead to high antibiotic persistence and affect therapy success. Proc Natl Acad Sci USA 118:e2014920118. doi:10.1073/pnas.2014920118 33574060 PMC7896289

[83] Dai Y, Gao C, Chen L, Chang W, Yu W, Ma X, Li J. 2019. Heterogeneous vancomycin-intermediate Staphylococcus aureus uses the VraSR regulatory system to modulate autophagy for increased intracellular survival in macrophage-like cell line RAW264.7. Front Microbiol 10:1222. doi:10.3389/fmicb.2019.01222 31214151 PMC6554704

[84] Shang Y, Wang X, Chen Z, Lyu Z, Lin Z, Zheng J, Wu Y, Deng Q, Yu Z, Zhang Y, Qu D. 2020. Staphylococcus aureus PhoU homologs regulate persister formation and virulence. Front Microbiol 11:11. doi:10.3389/fmicb.2020.00865 PMC7326077 32670206

[85] Frees D, Chastanet A, Qazi S, Sørensen K, Hill P, Msadek T, Ingmer H. 2004. Clp ATPases are required for stress tolerance, intracellular replication and biofilm formation in Staphylococcus aureus. Mol Microbiol 54:1445–1462. doi:10.1111/j.1365-2958.2004.04368.x 15554981

[86] Mashruwala AA, Eilers BJ, Fuchs AL, Norambuena J, Earle CA, van de Guchte A, Tripet BP, Copié V, Boyd JM. 2019. The ClpCP complex modulates respiratory metabolism in Staphylococcus aureus and is regulated in a SrrAB-dependent manner. J Bacteriol 201:15. doi:10.1128/JB.00188-19 PMC6620400 31109995

[87] Bai J, Zhu X, Zhao K, Yan Y, Xu T, Wang J, Zheng J, Huang W, Shi L, Shang Y, Lv Z, Wang X, Wu Y, Qu D. 2019. The role of ArlRS in regulating oxacillin susceptibility in methicillin-resistant Staphylococcus aureus indicates it is a potential target for antimicrobial resistance breakers. Emerg Microbes Infect 8:503–515. doi:10.1080/22221751.2019.1595984 30924407 PMC6455253

[88] Crosby HA, Tiwari N, Kwiecinski JM, Xu Z, Dykstra A, Jenul C, Fuentes EJ, Horswill AR. 2020. The Staphylococcus aureus ArlRS two-component system regulates virulence factor expression through MgrA. Mol Microbiol 113:103–122. doi:10.1111/mmi.14404 31618469 PMC7175635

[89] Schuster CF, Bellows LE, Tosi T, Campeotto I, Corrigan RM, Freemont P, Gründling A. 2016. The second messenger c-di-AMP inhibits the osmolyte uptake system OpuC in Staphylococcus aureus. Sci Signal 9:ra81. doi:10.1126/scisignal.aaf7279 27531650 PMC5248971

[90] Schlag S, Fuchs S, Nerz C, Gaupp R, Engelmann S, Liebeke M, Lalk M, Hecker M, Götz F. 2008. Characterization of the oxygen-responsive NreABC regulon of Staphylococcus aureus. J Bacteriol 190:7847–7858. doi:10.1128/JB.00905-08 18820014 PMC2583599

[91] Groma M, Horst SA, Das S, Huettel B, Klepsch M, Rudel T, Medina E, Fraunholz M. 2020. Identification of a Novel LysR-Type transcriptional regulator in Staphylococcus aureus that is crucial for secondary tissue colonization during metastatic bloodstream infection. mBio 11:e01646-20. doi:10.1128/mBio.01646-20 32843554 PMC7448277

[92] Beggs GA, Brennan RG, Arshad M. 2020. MarR family proteins are important regulators of clinically relevant antibiotic resistance. Protein Sci 29:647–653. doi:10.1002/pro.3769 31682303 PMC7020996

[93] Fritsch VN, Loi VV, Busche T, Sommer A, Tedin K, Nürnberg DJ, Kalinowski J, Bernhardt J, Fulde M, Antelmann H. 2019. The MarR-type repressor MhqR confers quinone and antimicrobial resistance in Staphylococcus aureus. Antioxid Redox Signal 31:1235–1252. doi:10.1089/ars.2019.7750 31310152 PMC6798810

[94] López E, Domenech A, Ferrándiz M-J, Frias MJ, Ardanuy C, Ramirez M, García E, Liñares J, de la Campa AG. 2014. Induction of prophages by fluoroquinolones in Streptococcus pneumoniae: implications for emergence of resistance in genetically-related clones. PLOS One 9:e94358. doi:10.1371/journal.pone.0094358 24718595 PMC3981806

[95] Panasenko OO, Bezrukov F, Komarynets O, Renzoni A. 2020. YjbH solubility controls Spx in Staphylococcus aureus: implication for MazEF toxin-antitoxin system regulation. Front Microbiol 11:113. doi:10.3389/fmicb.2020.00113 32117138 PMC7016130

[96] Kuroda M, Kuroda H, Oshima T, Takeuchi F, Mori H, Hiramatsu K. 2003. Two-component system VraSR positively modulates the regulation of cell-wall biosynthesis pathway in Staphylococcus aureus*.* Mol Microbiol 49:807–821. doi:10.1046/j.1365-2958.2003.03599.x 12864861

[97] Tomasz A. 2005. Role of penicillin-binding protein 2 (PBP2) in the antibiotic susceptibility and cell wall cross-linking of Staphylococcus aureus : evidence for the cooperative functioning of PBP2, PBP4, and PBP2A. J Bacteriol 187:1815–1824. doi:10.1128/JB.187.5.1815-1824.2005 15716453 PMC1064008

[98] Brown S, Santa Maria JP Jr, Walker S. 2013. Wall teichoic acids of gram-positive bacteria. Annu Rev Microbiol 67:313–336. doi:10.1146/annurev-micro-092412-155620 24024634 PMC3883102

[99] Stewart B, Rozen DE. 2012. Genetic variation for antibiotic persistence in Escherichia coli*.* Evolution 66:933–939. doi:10.1111/j.1558-5646.2011.01467.x 22380453

[100] Barth VC, Rodrigues BÁ, Bonatto GD, Gallo SW, Pagnussatti VE, Ferreira CAS, de Oliveira SD. 2014. Heterogeneous persister cells formation in Acinetobacter baumannii. PLOS ONE 8:e84361. doi:10.1371/journal.pone.0084361 PMC3877289 24391945

[101] Geerts N, De Vooght L, Passaris I, Delputte P, Van den Bergh B, Cos P. 2022. Antibiotic tolerance indicative of persistence is pervasive among clinical Streptococcus pneumoniae isolates and shows strong condition dependence. Microbiol Spectr 10:e02701–22. doi:10.1128/spectrum.02701-22 36374111 PMC9769776

[102] Zhang D, Hu Y, Zhu Q, Huang J, Chen Y. 2020. Proteomic interrogation of antibiotic resistance and persistence in Escherichia coli - progress and potential for medical research. Expert Rev Proteomics 17:393–409. doi:10.1080/14789450.2020.1784731 32567419

[103] Steward KF, Eilers B, Tripet B, Fuchs A, Dorle M, Rawle R, Soriano B, Balasubramanian N, Copié V, Bothner B, Hatzenpichler R. 2020. Metabolic implications of using BioOrthogonal non-canonical amino acid tagging (BONCAT) for tracking protein synthesis. Front Microbiol 11. doi:10.3389/fmicb.2020.00197 PMC7031258 32117186

[104] Wang M, Buist G, van Dijl JM. 2022. Staphylococcus aureus cell wall maintenance - the multifaceted roles of peptidoglycan hydrolases in bacterial growth, fitness, and virulence. FEMS Microbiol Rev 46:fuac025. doi:10.1093/femsre/fuac025 35675307 PMC9616470

[105] Dengler V, Meier PS, Heusser R, Berger-Bächi B, McCallum N. 2011. Induction kinetics of the Staphylococcus aureus cell wall stress stimulon in response to different cell wall active antibiotics. BMC Microbiol 11:16. doi:10.1186/1471-2180-11-16 21251258 PMC3032642

[106] Akhtar AA, Turner DP. 2022. The role of bacterial ATP-binding cassette (ABC) transporters in pathogenesis and virulence: therapeutic and vaccine potential. Microb Pathog 171:105734. doi:10.1016/j.micpath.2022.105734 36007845

[107] Wang B, Grant RA, Laub MT. 2020. ppGpp coordinates nucleotide and amino-acid synthesis in E. coli during starvation. Mol Cell 80:29–42. doi:10.1016/j.molcel.2020.08.005 32857952 PMC8362273

[108] Dörr T, Vulić M, Lewis K. 2010. Ciprofloxacin causes persister formation by inducing the TisB toxin in Escherichia coli. PLoS Biol 8:e1000317. doi:10.1371/journal.pbio.1000317 20186264 PMC2826370

[109] Hauryliuk V, Atkinson GC, Murakami KS, Tenson T, Gerdes K. 2015. Recent functional insights into the role of (p)ppGpp in bacterial physiology. Nat Rev Microbiol 13:298–309. doi:10.1038/nrmicro3448 25853779 PMC4659695

[110] Pacios O, Blasco L, Bleriot I, Fernandez-Garcia L, Ambroa A, López M, Bou G, Cantón R, Garcia-Contreras R, Wood TK, Tomás M. 2020. (P)ppGpp and its role in bacterial persistence: new challenges. Antimicrob Agents Chemother 64:10. doi:10.1128/AAC.01283-20 PMC7508602 32718971

[111] Lewis K. 2010. Persister cells. Annu Rev Microbiol 64:357–372. doi:10.1146/annurev.micro.112408.134306 20528688

[112] Keren I, Shah D, Spoering A, Kaldalu N, Lewis K. 2004. Specialized persister cells and the mechanism of multidrug tolerance in Escherichia coli. J Bacteriol 186:8172–8180. doi:10.1128/JB.186.24.8172-8180.2004 15576765 PMC532439

[113] Wessel D, Flügge UI. 1984. A method for the quantitative recovery of protein in dilute solution in the presence of detergents and lipids. Anal Biochem 138:141–143. doi:10.1016/0003-2697(84)90782-6 6731838

[114] Rappsilber J, Mann M, Ishihama Y. 2007. Protocol for micro-purification, enrichment, pre-fractionation and storage of peptides for proteomics using StageTips. Nat Protoc 2:1896–1906. doi:10.1038/nprot.2007.261 17703201

[115] Cox J, Neuhauser N, Michalski A, Scheltema RA, Olsen JV, Mann M. 2011. Andromeda: a peptide search engine integrated into the MaxQuant environment. J Proteome Res 10:1794–1805. doi:10.1021/pr101065j 21254760

