# Supplementary material for: Using BONCAT to dissect the proteome of S. aureus persisters
Source: mSphere. 2025 Sep 8;10(9):e00431-25. doi: 10.1128/msphere.00431-25 (PMC12482189; doi:10.1128/msphere.00431-25)
Supplement: Supplemental material — Supplemental figures and Tables S1 to S5. [file msphere.00431-25-s0001.docx]

**Supporting Information for: Using A BONCAT Approach To Dissect The Proteome Of *S. aureus* Persisters**

Eva D. C. George Matlalcuatzi ^1^, Thomas Bakkum^1^, Pooja S. Thomas^1^, Stephan Hacker^1^, Bogdan I. Florea^1^, Bastienne Vriesendorp^2^, Daniel E. Rozen^2*^, Sander I. van Kasteren^1*^

Author address: 1) Leiden Institute of Chemistry and The Institute of Chemical Immunology, Leiden University, Einsteinweg 55, Leiden, The Netherlands. 2) Institute of Biology Leiden, Leiden University, Sylviusweg 72, Leiden, The Netherlands

To whom correspondence should be addressed: DER: [d.e.rozen@biology.leidenuniv.nl](mailto:d.e.rozen@biology.leidenuniv.nl); SIvK: [s.i.van.kasteren@chem.leidenuniv.nl](mailto:s.i.van.kasteren@chem.leidenuniv.nl)

**Supplemental Figures**


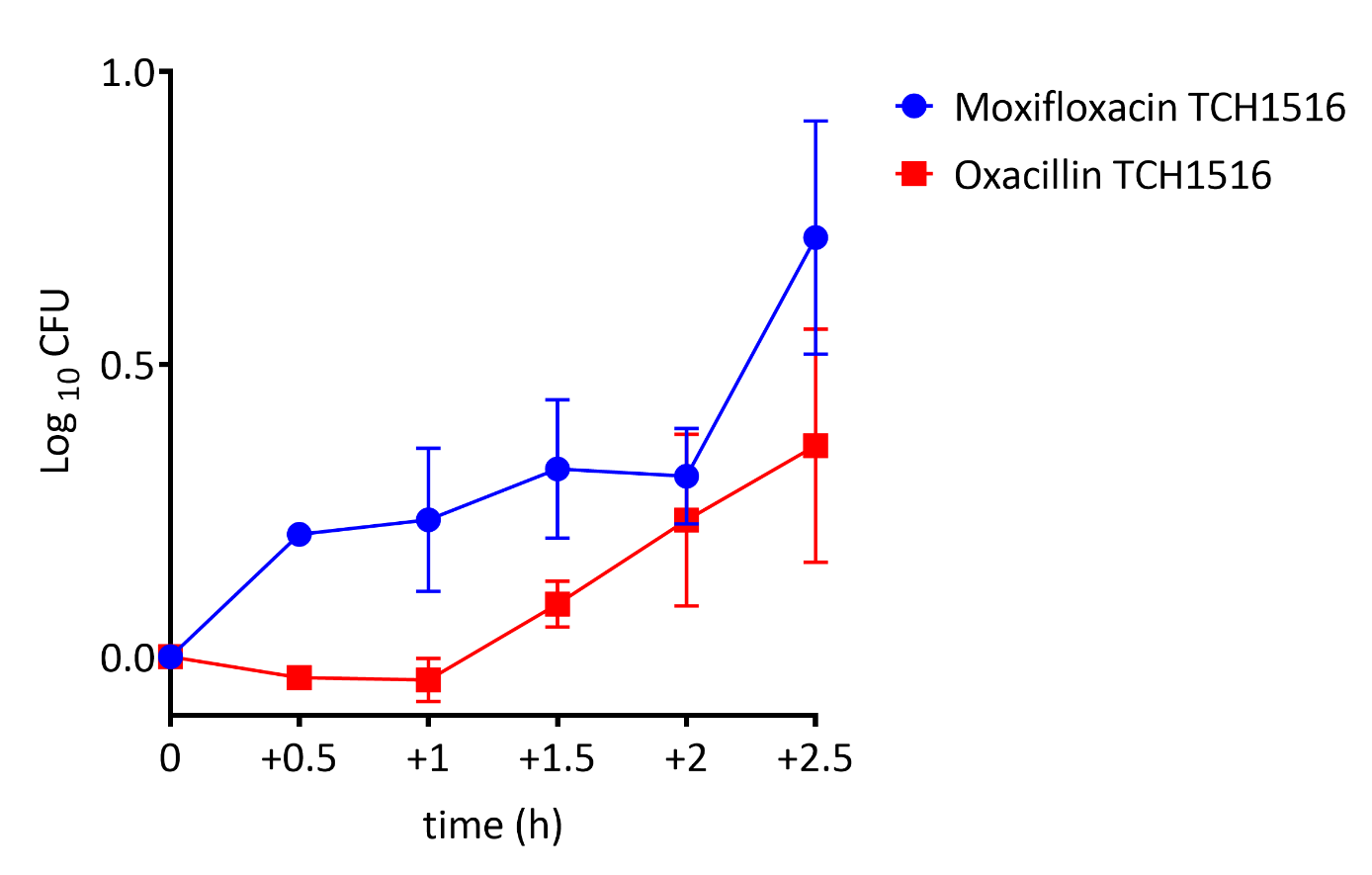


**Figure S1. Persister population is able to regrow after antibiotic treatment.** To demonstrate the ability of the persister population to regrowth following antibiotic removal, S. aureus was exposed to 50x the MIC of Moxifloxacin (blue) and Oxacillin (red) for 4 hours. After this treatment period, antibiotic was removed through bacterial pelleting and washing. The subsequent recovery phase was monitored for an additional 2.5 hours. The results indicate that bacterial persisters that emerged after 4 hours of antibiotic exposure are capable of resume growth once the antibiotic stress is removed. All experiments were performed with 4 biological replicates (n=4). Y-axis shows % of CFU with initial CFU set as 1 (in log scale).


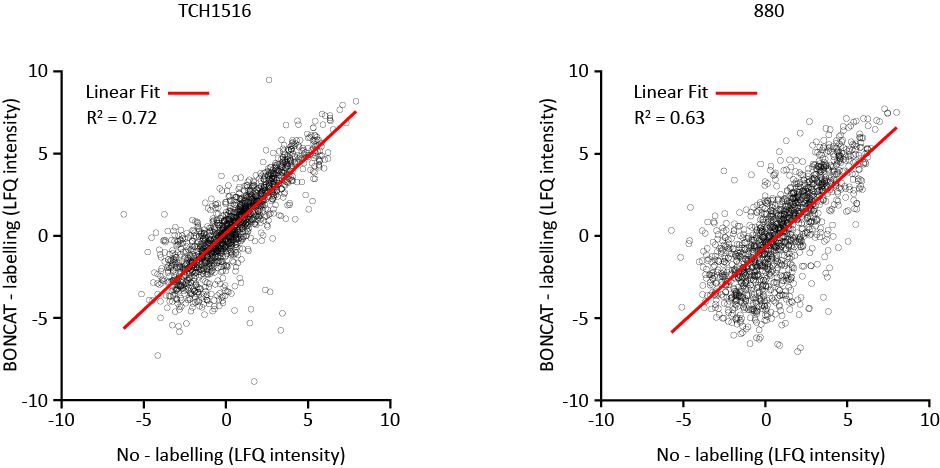


**a**

**b**

**Figure S2. Evaluation of BONCAT-MS on MRSA strains.** Correlation between LFQ intensities for BONCAT retrieved proteins compared to the recovery of non labelled proteins for strain TCH1516 (panel a) and 880 (panel b). The comparison shows that both methods are effective at obtaining MRSA proteome. The comparison shows a linear trend with a R^2^=0.7 and R^2^=0.6. All experiments were performed with 4 biological replicates (n=4).

**
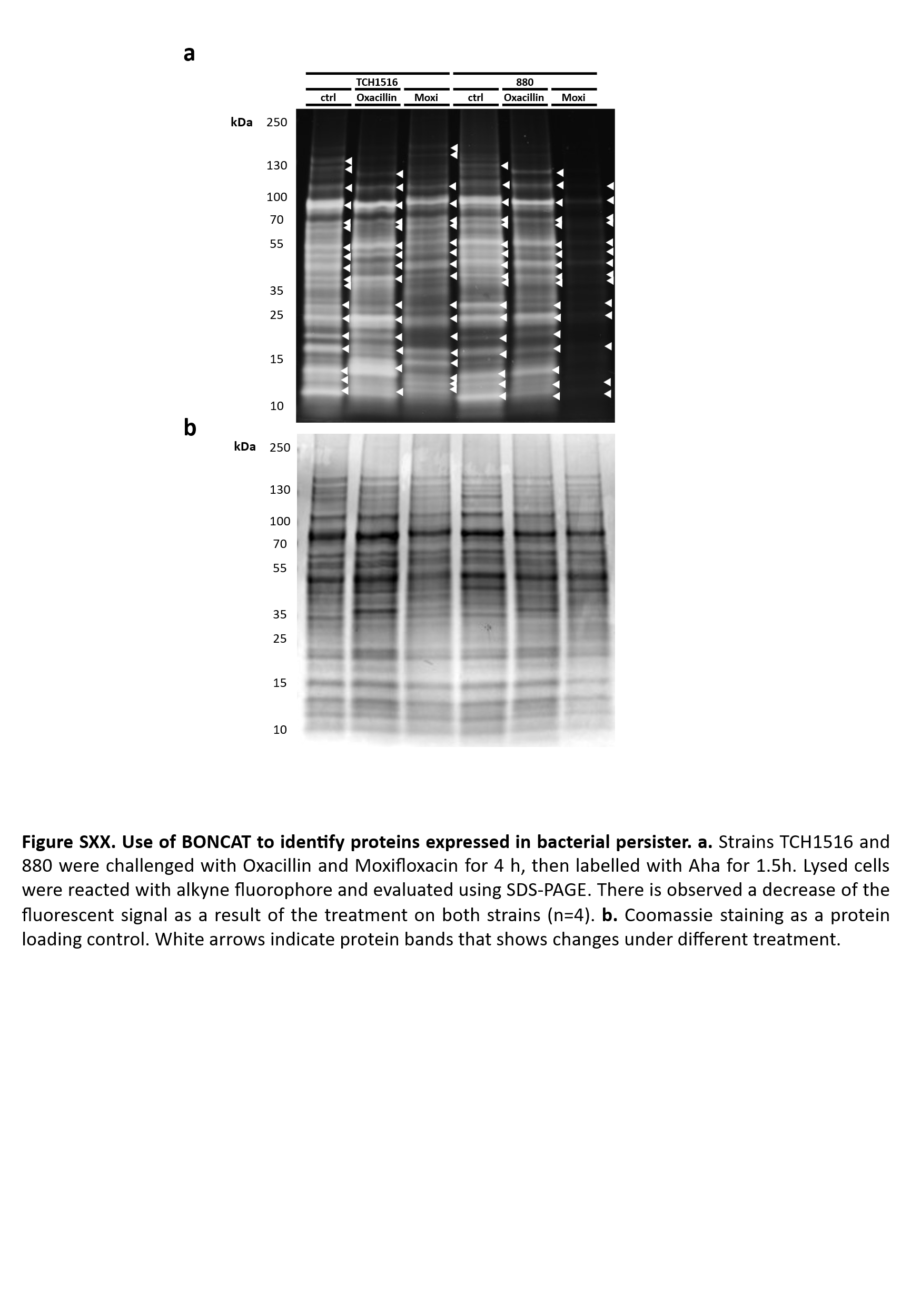
**

**Figure S3. Use of BONCAT to identify proteins expressed in bacterial persisters. a.** Strains TCH1516 and 880 were challenged with Oxacillin and Moxifloxacin for 4 h, then labelled with Aha for 1.5h. Lysed cells were reacted with alkyne fluorophore and evaluated using SDS-PAGE. There is observed a decrease of the fluorescent signal as a result of the treatment on both strains. **b.** Coomassie staining as a protein loading control. White arrows indicate protein bands that shows changes under different treatment. All experiments were performed with 4 biological replicates (n=4).


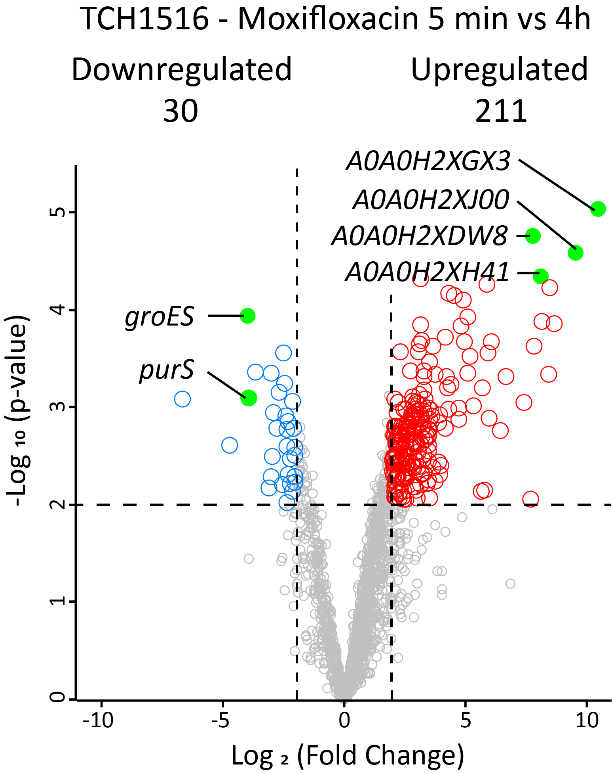


TCH1516 under moxifloxacin treatment

Comparison at early antibiotic treatment to persister state

**figure S4. Protein expression for TCH1516 at early moxifloxacin treatment compared to persister state.** Strain TCH1516 was selected to track the protein expression over time once antibiotic challenge is applied. To show persister specific proteins, a comparison at early stage of antibiotic treatment comprising 5-90 min compared to persister state at 240-330 min. The comparison revealed 211 upregulated and 30 downregulated proteins. Proteins highly significant are showed in green, for the upregulated proteins A0A0H2XDW8 and A0A0H2XH41 are pathogenicity island proteins related to bacterial virulence.[1, 2] A0A0H2XGX3 is a phage-related DNA recombination protein suspected to facilitate DNA recombination associated with bacteriophage activity in bacterial genome.[3] and A0A0H2XJ00 Is a conserved hypothetical phage protein.[4] For the downregulated proteins Q2FF94 (GroES) Plays an essential role in assisting protein folding and A0A0H2XGZ9 (PurS) is involved in the purines biosynthetic pathway.[5, 6] All the DEPs detected from this comparison are listed in table S9.

| **Table S1. Upregulated genes with no assigned pathway in TCH1516 under Oxacillin treatment** | | |
| --- | --- | --- |
| **Gene name** | **Protein name** | **log2 Fold Change (FC)** |
| A0A0H2XF08 | Uncharacterized protein | 8,21 |
| A0A0H2XGA4 | DUF4930 family protein | 8,00 |
| A0A0H2XIB2 | Uncharacterized protein | 5,41 |
| grpE | Protein GrpE (HSP-70 cofactor) | 5,06 |
| hrcA | Heat-inducible transcription repressor HrcA | 4,56 |
| msrA | Peptide methionine sulfoxide reductase MsrA | 4,32 |
| ftsL | Cell division protein FtsL | 4,07 |
| prsA | Foldase protein PrsA | 4,04 |
| fmt | Fmt protein | 3,96 |
| A0A0H2XHX8 | Putative membrane protein | 3,93 |
| A0A0H2XIC3 | Aromatic acid exporter family protein | 3,81 |
| A0A0H2XJS2 | Mechanosensitive ion channel family protein | 3,75 |
| msrB | Peptide methionine sulfoxide reductase MsrB | 3,69 |
| A0A0H2XGS4 | lysostaphin | 3,63 |
| A0A0H2XH05 | Putative long chain fatty acid-CoA ligase VraA (Acyl-CoA synthetase) | 3,61 |
| A0A0H2XIM3 | Uncharacterized protein | 3,51 |
| A0A0H2XH84 | Putative XpaC protein | 3,43 |
| Q2FFQ0 | UPF0342 protein | 3,43 |
| tcaA | Membrane-associated protein TcaA | 3,40 |
| mtlA | Mannitol-specific phosphotransferase enzyme IIA component (EIIA) | 3,36 |
| Q2FGA9 | UPF0297 protein SAUSA300_1574 | 3,25 |
| gatC | Aspartyl/glutamyl-tRNA(Asn/Gln) amidotransferase subunit C (Asp/Glu-ADT subunit C) | 3,24 |
| acyP | Acylphosphatase (Acylphosphate phosphohydrolase) | 3,21 |
| A0A0H2XG67 | Metal-dependent hydrolase | 3,19 |
| A0A0H2XGV4 | Serine protease HtrA-like | 3,10 |
| A0A0H2XIG8 | Staphylococcal protein | 3,09 |
| A0A0H2XDZ0 | NIF system FeS cluster assembly NifU C-terminal domain-containing protein | 3,04 |
| dinG | 3'-5' exonuclease DinG | 3,01 |
| A0A0H2XFB1 | Uncharacterized protein | 2,84 |
| trxA | Thioredoxin (Trx) | 2,79 |
| A0A0H2XJ93 | Alpha/beta hydrolase | 2,79 |
| A0A0H2XH00 | Fibrinogen-binding protein | 2,76 |
| A0A0H2XK13 | Isochorismatase-like domain-containing protein | 2,74 |
| A0A0H2XF59 | Putative membrane protein | 2,70 |
| A0A0H2XJ27 | Ferredoxin | 2,57 |
| A0A0H2XF96 | Cytosolic protein | 2,55 |
| A0A0H2XJ44 | Uncharacterized protein | 2,34 |
| crr | PTS system glucose-specific EIIA component (EIIA-Glc) (EIII-Glc) (Glucose-specific phosphotransferase enzyme IIA component) | 2,32 |
| A0A0H2XGW2 | Transcription regulatory protein | 2,31 |
| mnhA1 | Na(+)/H(+) antiporter subunit A1 (Mnh complex subunit A1) | 2,30 |
| A0A0H2XE76 | Glyoxalase family protein | 2,30 |
| A0A0H2XHI4 | Phage protein | 2,27 |
| A0A0H2XJD2 | Zinc metalloprotease | 2,27 |
| A0A0H2XG72 | D-serine/D-alanine/glycine transporter | 2,22 |
| A0A0H2XFM9 | DUF2197 domain-containing protein | 2,20 |
| A0A0H2XEI1 | CAP domain-containing protein | 2,19 |
| A0A0H2XJ35 | Staphylococcal protein | 2,17 |
| A0A0H2XHE3 | Transcriptional regulator, MerR family | 2,13 |
| A0A0H2XH45 | Putative lipoprotein | 2,11 |
| gltT | Proton/sodium-glutamate symport protein | 2,09 |
| A0A0H2XGW7 | Putative lipoprotein | 2,08 |
| Q2FH17 | UPF0346 protein SAUSA300_1314 | 2,06 |
| A0A0H2XFZ7 | Putative ABC transporter protein EcsB | 2,04 |
| pbp4 | Penicillin-binding protein 4 | 2,04 |
| tagX | Putative glycosyltransferase TagX (Teichoic acid biosynthesis protein X) | 2,03 |
| drp35 | Lactonase drp35 | 2,00 |
|  |  |  |
|  |  |  |
| **Downregulated genes with no assigned pathway in TCH1516 under Oxacillin treatment** | | |
| **Gene Name** | **Protein name** | **log2 Fold Change (FC)** |
| A0A0H2XKC1 | 5'-nucleotidase, lipoprotein e(P4) family | -9,84 |
| A0A0H2XHW2 | Antigen, 67 kDa | -8,92 |
| A0A0H2XH30 | Peptidase, M20/M25/M40 family | -6,93 |
| nrdD | Anaerobic ribonucleotide reductase, large subunit | -6,78 |
| A0A0H2XFY7 | Uncharacterized protein | -5,60 |
| isaA | Probable transglycosylase IsaA (Immunodominant staphylococcal antigen A) | -5,48 |
| A0A0H2XHH4 | Putative lipoprotein | -5,08 |
| essD (esaD) | Type VII secretion system protein EssD (Ess-associated gene D) (Nuclease toxin EssD) | -4,88 |
| sle1 (aaa) | N-acetylmuramoyl-L-alanine amidase sle1 | -4,83 |
| A0A0H2XEH7 | Staphopain A | -4,61 |
| A0A0H2XI72 | triacylglycerol lipase | -4,51 |
| A0A0H2XKN2 | Uncharacterized protein | -4,42 |
| esaA | Type VII secretion system accessory factor EsaA | -4,30 |
| A0A0H2XHA3 | N-acetylmuramoyl-L-alanine amidase | -4,29 |
| A0A0H2XI41 | Inosine-uridine preferring nucleoside hydrolase | -4,27 |
| cvfC | Conserved virulence factor C | -4,25 |
| A0A0H2XFU4 | TIGR01741 family protein | -4,21 |
| nirD | Nitrite reductase [NAD(P)H], small subunit | -4,16 |
| A0A0H2XF17 | Putative lipoprotein | -4,05 |
| A0A0H2XI18 | Acyl-CoA dehydrogenase | -4,04 |
| A0A0H2XJH7 | Immunoglobulin G-binding protein A (Staphylococcal protein A) | -3,97 |
| essB | Type VII secretion system protein EssB | -3,86 |
| A0A0H2XJW1 | Threonylcarbamoyl-AMP synthase (TC-AMP synthase) (L-threonylcarbamoyladenylate synthase) | -3,86 |
| scdA | Iron-sulfur cluster repair protein ScdA | -3,79 |
| A0A0H2XHM9 | Cytosolic protein | -3,64 |
| A0A0H2XH53 | Uncharacterized protein | -3,55 |
| rot | HTH-type transcriptional regulator rot (Repressor of toxins) | -3,53 |
| mscL | Large-conductance mechanosensitive channel | -3,50 |
| clpL | ATP-dependent Clp protease ATP-binding subunit ClpL | -3,50 |
| pflA | Pyruvate formate-lyase-activating enzyme (PFL-activating enzyme) | -3,40 |
| A0A0H2XFI6 | Type VII secretion system protein EsaE | -3,33 |
| A0A0H2XKB6 | Cytosolic protein | -3,32 |
| gap | Glyceraldehyde-3-phosphate dehydrogenase | -3,31 |
| gcvH | Glycine cleavage system H protein (Octanoyl/lipoyl carrier protein) | -3,28 |
| A0A0H2XI08 | Alcohol dehydrogenase, zinc-containing | -3,27 |
| Q2FHY3 | UPF0223 protein SAUSA300_0997 | -3,26 |
| nreB | Oxygen sensor histidine kinase NreB (Nitrogen regulation protein B) | -3,26 |
| A0A0H2XH89 | DNA-binding protein | -3,20 |
| esxA | Type VII secretion system extracellular protein A (Ess extracellular protein A) | -3,18 |
| ilvN | acetolactate synthase | -3,17 |
| A0A0H2XI37 | Esterase-like protein | -3,14 |
| A0A0H2XET3 | Lipoprotein | -3,14 |
| A0A0H2XHF8 | DUF961 domain-containing protein | -3,07 |
| A0A0H2XDI1 | Zn-binding lipoprotein adcA-like protein | -3,07 |
| A0A0H2XFW5 | Fructosamine kinase family protein | -3,07 |
| A0A0H2XE61 | ABC transporter, ATP-binding protein | -3,06 |
| A0A0H2XH59 | Uncharacterized protein | -3,05 |
| sdrD | Serine-aspartate repeat-containing protein D | -2,99 |
| fnbB | Fibronectin-binding protein B | -2,98 |
| thrS | Threonine--tRNA ligase (Threonyl-tRNA synthetase) (ThrRS) | -2,98 |
| A0A0H2XGS6 | TIGR01741 family protein | -2,97 |
| Q2FFA3 | Uncharacterized leukocidin-like protein 1 | -2,95 |
| A0A0H2XK08 | Oxidoreductase, short chain dehydrogenase/reductase family | -2,92 |
| A0A0H2XHT0 | ABC transporter, ATP-binding protein | -2,91 |
| A0A0H2XI02 | 5'-nucleotidase family protein | -2,89 |
| A0A0H2XDW9 | DinB-like domain-containing protein | -2,84 |
| A0A0H2XKK6 | nitric oxide dioxygenase | -2,73 |
| A0A0H2XIF5 | Uncharacterized protein | -2,72 |
| oppD | Oligopeptide ABC transporter, ATP-binding protein | -2,71 |
| A0A0H2XFW4 | Peptidase, U32 family | -2,70 |
| A0A0H2XE79 | Uncharacterized protein | -2,69 |
| treC | Alpha,alpha-phosphotrehalase | -2,68 |
| Q2FDN2 | Uncharacterized protein SAUSA300_2562 | -2,68 |
| A0A0H2XHA6 | DUF4176 domain-containing protein | -2,64 |
| A0A0H2XIC1 | Amino acid ABC transporter, ATP-binding protein | -2,63 |
| A0A0H2XGJ8 | Uncharacterized protein | -2,63 |
| A0A0H2XJF7 | Putative membrane protein | -2,62 |
| A0A0H2XHF6 | Secretory antigen SsaA | -2,59 |
| adh | Alcohol dehydrogenase (ADH) | -2,59 |
| A0A0H2XDX3 | Uncharacterized protein | -2,58 |
| lytR | Transcriptional regulatory protein LytR (Sensory transduction protein LytR) | -2,58 |
| esxB | Type VII secretion system extracellular protein B (Ess extracellular protein B) | -2,58 |
| spoVG | Putative septation protein SpoVG | -2,55 |
| A0A0H2XIB6 | ABC transporter, ATP-binding protein | -2,55 |
| A0A0H2XKD6 | TIGR01741 family protein | -2,52 |
| atl | Bifunctional autolysin | -2,51 |
| Q2FJJ6 | Uncharacterized lipoprotein SAUSA300_0419 | -2,49 |
| A0A0H2XG24 | Uncharacterized protein | -2,48 |
| A0A0H2XIY3 | HAD-superfamily hydrolase, subfamily IA, variant 1 | -2,46 |
| nuc | Thermonuclease (Micrococcal nuclease) (Staphylococcal nuclease) | -2,44 |
| A0A0H2XH82 | Putative lipoprotein | -2,43 |
| cspA | Cold shock protein CspA | -2,43 |
| A0A0H2XJN3 | Glycolytic operon regulator | -2,41 |
| ssaA | Secretory antigen SsaA | -2,39 |
| sodM | Superoxide dismutase [Mn/Fe] 2 | -2,38 |
| esxD | Type VII secretion system extracellular protein D (Ess extracellular protein D) | -2,36 |
| nreC | Oxygen regulatory protein NreC (Nitrogen regulation protein C) | -2,33 |
| A0A0H2XJE5 | NAD(P)-binding domain-containing protein | -2,31 |
| narJ | Respiratory nitrate reductase, delta subunit | -2,30 |
| A0A0H2XI33 | 5' nucleotidase family protein | -2,29 |
| Q2FF14 | UPF0340 protein SAUSA300_2068 | -2,28 |
| nrdG | Anaerobic ribonucleoside-triphosphate reductase-activating protein | -2,20 |
| Q2FDS6 | Uncharacterized hydrolase SAUSA300_2518 | -2,18 |
| A0A0H2XHJ8 | Peptidase, M16 family | -2,16 |
| A0A0H2XFV1 | HAD-superfamily hydrolase, subfamily IA, variant 1 | -2,15 |
| A0A0H2XFN1 | Persulfide-sensing transcriptional repressor CstR | -2,14 |
| A0A0H2XJP7 | Hydrolase, haloacid dehalogenase-like family | -2,14 |
| Q2FIJ2 | Organic hydroperoxide resistance protein-like | -2,13 |
| A0A0H2XFT0 | HTH-type transcriptional regulator | -2,12 |
| esxC (esaC) | Type VII secretion system extracellular protein C (Ess extracellular protein C) | -2,12 |
| A0A0H2XI29 | Acetyltransferase | -2,11 |
| A0A0H2XJ90 | D-lactate dehydrogenase (D-specific 2-hydroxyacid dehydrogenase) | -2,10 |
| A0A0H2XIF0 | MutT/nudix family protein | -2,08 |
| A0A0H2XEV3 | Type VII secretion system protein EssC | -2,08 |
| A0A0H2XIJ2 | Uncharacterized protein | -2,08 |
| A0A0H2XH40 | Uncharacterized protein | -2,03 |
| A0A0H2XFC0 | ABC transporter, ATP-binding protein, MsbA family | -2,03 |
| sceD | Probable transglycosylase SceD | -2,00 |

| **Table S2. Upregulated genes with no assigned pathway in TCH1516 under Moxifloxacin treatment** | | |
| --- | --- | --- |
| **Gene name** | **Protein name** | **log2 Fold Change (FC)** |
| A0A0H2XFK3 | Phi77 ORF014-like protein, phage anti-repressor protein | 8,77 |
| A0A0H2XI78 | PhiPVL ORF41-like protein | 8,76 |
| A0A0H2XGX3 | Putative phage-related DNA recombination protein | 8,59 |
| A0A0H2XJ00 | Conserved hypothetical phage protein | 7,77 |
| A0A0H2XIZ5 | Putative phage transcriptional regulator | 7,52 |
| A0A0H2XGG0 | PhiPVL ORF39-like protein | 7,34 |
| coa | Staphylocoagulase | 6,94 |
| A0A0H2XDW8 | Pathogenicity island protein | 6,81 |
| A0A0H2XF08 | Uncharacterized protein | 6,56 |
| A0A0H2XGA4 | DUF4930 family protein | 6,51 |
| Q2FIG2 | UPF0337 protein SAUSA300_0816 | 6,40 |
| A0A0H2XIJ7 | Conserved hypothetical phage protein | 5,88 |
| A0A0H2XH41 | Pathogenicity island protein | 5,07 |
| A0A0H2XFG8 | PhiPVL ORF050-like protein | 4,78 |
| Q2FHW8 | UPF0358 protein SAUSA300_1012 | 4,66 |
| A0A0H2XJY5 | Single-stranded DNA-binding protein (SSB) | 4,51 |
| A0A0H2XFB1 | Uncharacterized protein | 4,50 |
| Q2FGA1 | UPF0337 protein SAUSA300_1582 | 4,36 |
| A0A0H2XJ35 | Staphylococcal protein | 4,28 |
| nrdI | Protein NrdI | 4,26 |
| A0A0H2XJ47 | Veg protein | 4,23 |
| A0A0H2XKL2 | YolD-like family protein | 4,21 |
| A0A0H2XIZ8 | Putative DNA primase | 3,88 |
| A0A0H2XGY6 | Pathogenicity island protein | 3,85 |
| rpmI | Large ribosomal subunit protein bL35 (50S ribosomal protein L35) | 3,83 |
| A0A0H2XK54 | Pathogenicity island protein | 3,59 |
| fdhD | Sulfur carrier protein FdhD | 3,54 |
| trhO | tRNA uridine(34) hydroxylase (tRNA hydroxylation protein O) | 3,47 |
| A0A0H2XG02 | Uncharacterized protein | 3,45 |
| Q2FJ50 | UPF0741 protein SAUSA300_0575 | 3,26 |
| ssaA | Secretory antigen SsaA | 3,16 |
| A0A0H2XHX8 | Putative membrane protein | 3,13 |
| Q2FFM9 | Putative multidrug export ATP-binding/permease protein SAUSA300_1847 | 3,00 |
| A0A0H2XDZ0 | NIF system FeS cluster assembly NifU C-terminal domain-containing protein | 2,94 |
| A0A0H2XIH0 | Putative membrane protein | 2,94 |
| A0A0H2XKH6 | Universal stress protein family | 2,93 |
| A0A0H2XIP5 | Uncharacterized protein | 2,87 |
| A0A0H2XKN2 | Uncharacterized protein | 2,86 |
| A0A0H2XJ51 | Phage capsid protein | 2,78 |
| A0A0H2XJ27 | Ferredoxin | 2,75 |
| A0A0H2XI31 | Transcriptional regulator, TetR family | 2,75 |
| A0A0H2XJ44 | Uncharacterized protein | 2,74 |
| A0A0H2XED4 | Chitinase-related protein | 2,67 |
| A0A0H2XEB8 | Pathogenicity island protein | 2,67 |
| rpsR | Small ribosomal subunit protein bS18 (30S ribosomal protein S18) | 2,65 |
| ispE | Putative 4-diphosphocytidyl-2-C-methyl-D-erythritol kinase (CMK) | 2,58 |
| A0A0H2XEP1 | Uncharacterized protein | 2,54 |
| A0A0H2XK94 | Conserved hypothetical phage protein | 2,50 |
| A0A0H2XJA4 | Cytochrome D ubiquinol oxidase, subunit II | 2,49 |
| A0A0H2XHF7 | PhiPV083 ORF027-like protein | 2,46 |
| A0A0H2XK13 | Isochorismatase-like domain-containing protein | 2,45 |
| gatC | Aspartyl/glutamyl-tRNA(Asn/Gln) amidotransferase subunit C (Asp/Glu-ADT subunit C) | 2,44 |
| A0A0H2XDY4 | Uncharacterized protein | 2,43 |
| A0A0H2XFD4 | DUF4889 domain-containing protein | 2,42 |
| A0A0H2XGV0 | Putative membrane protein | 2,42 |
| sdaAA | L-serine dehydratase | 2,40 |
| Q2FFP9 | UPF0754 membrane protein SAUSA300_1796 | 2,38 |
| A0A0H2XFW3 | Aminoglycoside phosphotransferase domain-containing protein | 2,37 |
| pbp4 | Penicillin-binding protein 4 | 2,34 |
| cls | Cardiolipin synthase (CL synthase) | 2,34 |
| A0A0H2XGY2 | Abi family protein | 2,33 |
| spxA (spx) | Global transcriptional regulator Spx | 2,33 |
| A0A0H2XES5 | Transcriptional regulator, MarR family | 2,29 |
| rsmI | Ribosomal RNA small subunit methyltransferase I | 2,29 |
| rplB | Large ribosomal subunit protein uL2 (50S ribosomal protein L2) | 2,25 |
| A0A0H2XFZ7 | Putative ABC transporter protein EcsB | 2,24 |
| A0A0H2XHN7 | CBS domain protein | 2,23 |
| A0A0H2XJU8 | Lytic regulatory protein | 2,23 |
| fmt | Fmt protein | 2,21 |
| A0A0H2XIC8 | Staphylococcal protein | 2,20 |
| A0A0H2XIC9 | Putative teichoic acid biosynthesis protein | 2,19 |
| dps | General stress protein 20U | 2,19 |
| A0A0H2XGZ7 | Aminotransferase, class V | 2,19 |
| A0A0H2XH49 | Phi77 ORF026-like protein, putative phage transcriptional activator | 2,19 |
| dinG | 3'-5' exonuclease DinG | 2,15 |
| recQ | DNA helicase | 2,15 |
| pyc | Pyruvate carboxylase | 2,14 |
| A0A0H2XGN9 | Exonuclease | 2,12 |
| A0A0H2XGZ3 | Formate/nitrite transporter family protein | 2,08 |
| A0A0H2XJA0 | Luciferase-like domain-containing protein | 2,07 |
| saeS | Histidine protein kinase SaeS (Sensor protein SaeS) (Staphylococcus exoprotein expression protein S) | 2,07 |
| A0A0H2XHP7 | Staphylococcal complement inhibitor | 2,06 |
| rpoY | DNA-directed RNA polymerase subunit epsilon (RNAP epsilon subunit) (RNA polymerase epsilon subunit) (Transcriptase subunit epsilon) | 2,05 |
| nirR | Nitrite reductase transcriptional regulator NirR | 2,05 |
| rplD | Large ribosomal subunit protein uL4 (50S ribosomal protein L4) | 2,04 |
| A0A0H2XG71 | Metallo-beta-lactamase family protein | 2,04 |
| A0A0H2XHI4 | Phage protein | 2,03 |
| A0A0H2XGJ9 | Putative transcriptional regulator | 2,02 |
| secF (secD) | Multifunctional fusion protein [Includes: Protein translocase subunit SecD; Protein-export membrane protein SecF] | 2,01 |
| ear | Ear protein | 2,01 |
| **Downregulated genes with no assigned pathway in TCH1516 under Moxifloxacin treatment** | | |
| **Gene name** | **Protein name** | **log2 Fold Change (FC)** |
| A0A0H2XFV1 | HAD-superfamily hydrolase, subfamily IA, variant 1 | -6,27 |
| Q2FFA3 | Uncharacterized leukocidin-like protein 1 | -6,04 |
| oppA | Oligopeptide ABC transporter, substrate-binding protein | -5,40 |
| A0A0H2XFY7 | Uncharacterized protein | -5,39 |
| nrdD | Anaerobic ribonucleotide reductase, large subunit (EC 1.17.4.2) | -5,33 |
| Q2FFA2 | Uncharacterized leukocidin-like protein 2 | -5,31 |
| essD (esaD) | Type VII secretion system protein EssD (Ess-associated gene D) (Nuclease toxin EssD) | -5,13 |
| A0A0H2XKC1 | 5'-nucleotidase, lipoprotein e(P4) family | -4,87 |
| A0A0H2XFU8 | Putative homoserine O-acetyltransferase | -4,72 |
| oppF | Oligopeptide ABC transporter, ATP-binding protein | -4,69 |
| A0A0H2XE76 | Glyoxalase family protein (EC 4.4.1.5) | -4,67 |
| A0A0H2XFI6 | Type VII secretion system protein EsaE | -4,65 |
| oppD | Oligopeptide ABC transporter, ATP-binding protein | -4,59 |
| A0A0H2XEA1 | Lipoprotein | -4,57 |
| esaA | Type VII secretion system accessory factor EsaA | -4,55 |
| Q2FIJ2 | Organic hydroperoxide resistance protein-like | -4,44 |
| A0A0H2XJX6 | Aminotransferase, class V | -4,42 |
| A0A0H2XKB6 | Cytosolic protein | -4,34 |
| A0A0H2XI02 | 5'-nucleotidase family protein | -4,30 |
| A0A0H2XHM9 | Cytosolic protein | -4,24 |
| A0A0H2XEV3 | Type VII secretion system protein EssC | -4,18 |
| mnhD1 | Na(+)/H(+) antiporter subunit D1 (Mnh complex subunit D1) | -4,11 |
| clpL | ATP-dependent Clp protease ATP-binding subunit ClpL | -4,10 |
| A0A0H2XJK3 | Conserved hypothetical phage protein | -4,04 |
| esxA | Type VII secretion system extracellular protein A (Ess extracellular protein A) | -3,99 |
| A0A0H2XH30 | Peptidase, M20/M25/M40 family | -3,98 |
| A0A0H2XFY9 | Aminotransferase (EC 2.6.1.-) | -3,92 |
| metN2 | Methionine import ATP-binding protein MetN 2 | -3,85 |
| A0A0H2XHP5 | DNA-binding response regulator | -3,83 |
| A0A0H2XFY2 | ABC transporter, ATP-binding protein | -3,81 |
| A0A0H2XIP0 | phosphoenolpyruvate--glycerone phosphotransferase | -3,80 |
| cspA | Cold shock protein CspA | -3,68 |
| A0A0H2XI72 | triacylglycerol lipase | -3,53 |
| isaB | Immunodominant staphylococcal antigen B | -3,51 |
| A0A0H2XDX3 | Uncharacterized protein | -3,45 |
| A0A0H2XH76 | Uncharacterized protein | -3,45 |
| A0A0H2XEH7 | Staphopain A | -3,42 |
| A0A0H2XHF6 | Secretory antigen SsaA | -3,41 |
| A0A0H2XH89 | DNA-binding protein | -3,39 |
| Q2FH40 | Uncharacterized hydrolase SAUSA300_1291 | -3,37 |
| rpsA | Small ribosomal subunit protein bS1 (30S ribosomal protein S1) | -3,36 |
| sdrD | Serine-aspartate repeat-containing protein D | -3,35 |
| ilvN | acetolactate synthase | -3,28 |
| A0A0H2XG13 | Acetyltransferase, GNAT family | -3,27 |
| A0A0H2XHW2 | Antigen, 67 kDa | -3,25 |
| A0A0H2XFB9 | DNA-directed RNA polymerase subunit delta | -3,24 |
| A0A0H2XHH4 | Putative lipoprotein | -3,14 |
| esxD | Type VII secretion system extracellular protein D (Ess extracellular protein D) | -3,09 |
| groES (groS) | Co-chaperonin GroES (10 kDa chaperonin) (Chaperonin-10) (Cpn10) | -3,06 |
| hysA | Hyaluronate lyase | -3,05 |
| atl | Bifunctional autolysin | -3,02 |
| groEL (groL) | Chaperonin GroEL (60 kDa chaperonin) (Chaperonin-60) (Cpn60) | -3,02 |
| Q2FHK2 | UPF0122 protein SAUSA300_1129 | -2,99 |
| secG | Protein-export membrane protein SecG | -2,99 |
| A0A0H2XI08 | Alcohol dehydrogenase, zinc-containing | -2,98 |
| opuCa | Glycine betaine/carnitine/choline ABC transporter ATP-binding protein | -2,91 |
| A0A0H2XGT6 | Carboxymuconolactone decarboxylase-like domain-containing protein | -2,87 |
| A0A0H2XHY3 | Glycerate kinase | -2,85 |
| hpf | Ribosome hibernation promotion factor (HPF) (Ribosome hibernation-promoting factor) | -2,85 |
| A0A0H2XG24 | Uncharacterized protein | -2,85 |
| agrA | Accessory gene regulator protein A | -2,84 |
| A0A0H2XH25 | TIGR01741 family protein | -2,77 |
| A0A0H2XFR9 | YwpF protein | -2,75 |
| A0A0H2XJ90 | D-lactate dehydrogenase | -2,72 |
| Q2FJJ6 | Uncharacterized lipoprotein SAUSA300_0419 | -2,71 |
| A0A0H2XJX2 | DNA-binding protein | -2,71 |
| A0A0H2XH81 | Heat shock protein | -2,66 |
| graR | Response regulator protein GraR (Glycopeptide resistance-associated protein R) | -2,66 |
| A0A0H2XHE8 | MutT/nudix family protein | -2,64 |
| nrdG | Anaerobic ribonucleoside-triphosphate reductase-activating protein | -2,63 |
| A0A0H2XHA3 | N-acetylmuramoyl-L-alanine amidase | -2,60 |
| A0A0H2XFP7 | Transcriptional regulator, MarR family | -2,55 |
| A0A0H2XGJ1 | PhiSLT ORF104a-like protein, repressor | -2,55 |
| A0A0H2XIT3 | Diacetyl reductase [(S)-acetoin forming] | -2,54 |
| A0A0H2XIY3 | HAD-superfamily hydrolase, subfamily IA, variant 1 | -2,53 |
| opuCc | Glycine betaine/carnitine/choline ABC transporter | -2,52 |
| nagD | Acid sugar phosphatase | -2,52 |
| ptpA | Low molecular weight protein-tyrosine-phosphatase PtpA (Phosphotyrosine phosphatase A) (PTPase A) | -2,51 |
| Q2FE21 | Uncharacterized oxidoreductase SAUSA300_2422 | -2,48 |
| A0A0H2XFM9 | DUF2197 domain-containing protein | -2,47 |
| A0A0H2XG03 | Glycine cleavage H-protein | -2,45 |
| essB | Type VII secretion system protein EssB | -2,44 |
| A0A0H2XEU1 | Acetyltransferase, GNAT family | -2,44 |
| A0A0H2XJ60 | Putative arsenate reductase | -2,39 |
| A0A0H2XFT9 | Lipoprotein | -2,38 |
| A0A0H2XFJ9 | Peptidase, M20/M25/M40 family | -2,35 |
| ctsR | Transcriptional regulator CtsR | -2,33 |
| A0A0H2XFK7 | Transcriptional regulator, MarR family | -2,33 |
| A0A0H2XI29 | Acetyltransferase | -2,33 |
| msrA | Peptide methionine sulfoxide reductase MsrA | -2,32 |
| A0A0H2XH27 | Phage protein | -2,30 |
| A0A0H2XET3 | Lipoprotein | -2,28 |
| A0A0H2XG94 | Transcriptional regulator, PadR family | -2,27 |
| A0A0H2XF36 | Uncharacterized protein | -2,26 |
| A0A0H2XFJ6 | Aldo/keto reductase family protein (EC 1.1.1.218) | -2,26 |
| mraZ | Transcriptional regulator MraZ | -2,25 |
| gcvH | Glycine cleavage system H protein (Octanoyl/lipoyl carrier protein) | -2,25 |
| A0A0H2XHH5 | Putative lipoprotein | -2,24 |
| arlR | Response regulator ArlR | -2,21 |
| A0A0H2XKB1 | DUF1641 domain-containing protein | -2,17 |
| gcvT | Aminomethyltransferase (Glycine cleavage system T protein) | -2,16 |
| A0A0H2XHS7 | thioredoxin-dependent peroxiredoxin (Thioredoxin peroxidase) | -2,16 |
| A0A0H2XHL1 | Uncharacterized protein | -2,16 |
| pxpA | 5-oxoprolinase subunit A (5-OPase subunit A) (5-oxoprolinase (ATP-hydrolyzing) subunit A) | -2,15 |
| A0A0H2XFB7 | Dihydroxyacetone kinase, DhaL subunit (EC 2.7.1.-) | -2,11 |
| mcsB | Protein-arginine kinase | -2,11 |
| atpD | ATP synthase subunit beta (ATP synthase F1 sector subunit beta) (F-ATPase subunit beta) | -2,10 |
| A0A0H2XI48 | ABC transporter, substrate-binding protein | -2,10 |
| ndk | Nucleoside diphosphate kinase (NDK) (NDP kinase) (Nucleoside-2-P kinase) | -2,09 |
| A0A0H2XH88 | ABC transporter, ATP-binding protein | -2,07 |
| narT (narK) | Probable nitrate transporter NarT | -2,05 |
| rot | HTH-type transcriptional regulator rot (Repressor of toxins) | -2,04 |
| Q2FJG3 | Nucleoid-associated protein SAUSA300_0453 | -2,04 |
| hchA | Protein/nucleic acid deglycase HchA (Maillard deglycase) | -2,03 |
| Q2FIF3 | Probable nitronate monooxygenase (NMO) (Propionate 3-nitronate monooxygenase) (P3N monooxygenase) | -2,02 |
| A0A0H2XGJ6 | Phosphosugar-binding transcriptional regulator, RpiR family | -2,02 |
| A0A0H2XIC1 | Amino acid ABC transporter, ATP-binding protein | -2,02 |
| A0A0H2XJP4 | Putative thioredoxin | -2,02 |
| mtlD | Mannitol-1-phosphate 5-dehydrogenase | -2,01 |
| A0A0H2XGT0 | SIS domain protein | -2,00 |

| **Table S3. Upregulated genes with no assigned pathway in 880 under Oxacillin treatment** | | |
| --- | --- | --- |
| **Gene Name** | **Protein name** | **log2 Fold Change (FC)** |
| A0A0H2XIB2 | Uncharacterized protein | 5,40 |
| A0A0H2XFZ6 | Transcriptional regulator | 4,32 |
| A0A0H2XF08 | Uncharacterized protein | 4,25 |
| A0A0H2XI01 | N-acetylmuramoyl-L-alanine amidase domain protein | 3,73 |
| topA | DNA topoisomerase 1 | 3,22 |
| A0A0H2XJG9 | Maebl | 3,06 |
| drp35 | Lactonase drp35 | 3,05 |
| A0A0H2XIG8 | Staphylococcal protein | 2,97 |
| A0A0H2XH00 | Fibrinogen-binding protein | 2,87 |
| A0A0H2XEB3 | Cytosolic protein | 2,76 |
| A0A0H2XE91 | DUF3139 domain-containing protein | 2,65 |
| A0A0H2XJ44 | Uncharacterized protein | 2,64 |
| A0A0H2XJU8 | Lytic regulatory protein | 2,40 |
| prsA | Foldase protein PrsA | 2,37 |
| Q2FHW8 | UPF0358 protein SAUSA300_1012 | 2,33 |
| Q2FJ50 | UPF0741 protein SAUSA300_0575 | 2,27 |
| ftsL | Cell division protein FtsL | 2,18 |
| A0A0H2XIM3 | Uncharacterized protein | 2,13 |
| **Downregulated genes with no assigned pathway in 880 under Oxacillin treatment** | | |
| **Gene Name** | **Protein name** | **log2 Fold Change (FC)** |
| scdA | Iron-sulfur cluster repair protein ScdA | -9,65 |
| A0A0H2XHM2 | ABC transporter, ATP-binding protein | -7,36 |
| A0A0H2XFB2 | Uncharacterized protein | -7,03 |
| A0A0H2XGU3 | Uncharacterized protein | -6,92 |
| A0A0H2XJ11 | Phage protein | -6,78 |
| ptpA | Low molecular weight protein-tyrosine-phosphatase PtpA (Phosphotyrosine phosphatase A) (PTPase A) | -6,74 |
| sarR | HTH-type transcriptional regulator SarR (Staphylococcal accessory regulator R) | -6,69 |
| A0A0H2XIY3 | HAD-superfamily hydrolase, subfamily IA, variant 1 | -6,58 |
| A0A0H2XI35 | Phage protein | -6,42 |
| Q2FJJ6 | Uncharacterized lipoprotein SAUSA300_0419 | -6,26 |
| A0A0H2XHC6 | D-histidine (S)-2-aminobutanoyltransferase CntL | -6,12 |
| rot | HTH-type transcriptional regulator rot (Repressor of toxins) | -6,09 |
| A0A0H2XKC1 | 5'-nucleotidase, lipoprotein e(P4) family | -6,07 |
| A0A0H2XJ77 | SAP domain protein | -6,00 |
| A0A0H2XH30 | Peptidase, M20/M25/M40 family | -5,99 |
| A0A0H2XIB6 | ABC transporter, ATP-binding protein | -5,78 |
| A0A0H2XHE7 | Rhodanese-like domain protein | -5,68 |
| A0A0H2XDX3 | Uncharacterized protein | -5,65 |
| A0A0H2XFE3 | Thioesterase domain-containing protein | -5,64 |
| nirD | Nitrite reductase [NAD(P)H], small subunit | -5,60 |
| oppD | Oligopeptide ABC transporter, ATP-binding protein | -5,60 |
| A0A0H2XFR6 | GntR family regulatory protein | -5,48 |
| arsR | Transcriptional repressor, ArsR family | -5,29 |
| A0A0H2XFM9 | DUF2197 domain-containing protein | -5,26 |
| pflA | Pyruvate formate-lyase-activating enzyme (PFL-activating enzyme) | -5,16 |
| A0A0H2XI94 | Glyoxalase family protein | -5,03 |
| A0A0H2XHF8 | DUF961 domain-containing protein | -5,00 |
| recR | Recombination protein RecR | -4,99 |
| nrdD | Anaerobic ribonucleotide reductase, large subunit | -4,89 |
| A0A0H2XHM9 | Cytosolic protein | -4,88 |
| rpmI | Large ribosomal subunit protein bL35 (50S ribosomal protein L35) | -4,87 |
| cdd | Cytidine deaminase (Cytidine aminohydrolase) | -4,85 |
| azoR | FMN-dependent NADH:quinone oxidoreductase | -4,79 |
| Q2FI72 | UPF0738 protein SAUSA300_0906 | -4,79 |
| A0A0H2XHZ2 | TfoX N-terminal domain-containing protein | -4,75 |
| A0A0H2XFW5 | Fructosamine kinase family protein | -4,63 |
| adh | Alcohol dehydrogenase (ADH) | -4,58 |
| gap | Glyceraldehyde-3-phosphate dehydrogenase | -4,57 |
| cobB | NAD-dependent protein deacetylase | -4,56 |
| luxS | S-ribosylhomocysteine lyase (AI-2 synthesis protein) (Autoinducer-2 production protein LuxS) | -4,52 |
| A0A0H2XI29 | Acetyltransferase | -4,52 |
| A0A0H2XGJ8 | Uncharacterized protein | -4,48 |
| A0A0H2XIF0 | MutT/nudix family protein | -4,45 |
| Q2FHK2 | UPF0122 protein SAUSA300_1129 | -4,44 |
| Q2FGB1 | UPF0473 protein SAUSA300_1572 | -4,42 |
| cvfC | Conserved virulence factor C | -4,36 |
| rpmG2 | Large ribosomal subunit protein bL33B (50S ribosomal protein L33 2) | -4,34 |
| A0A0H2XEP1 | Uncharacterized protein | -4,33 |
| graR | Response regulator protein GraR (Glycopeptide resistance-associated protein R) | -4,31 |
| A0A0H2XH89 | DNA-binding protein | -4,31 |
| A0A0H2XFW8 | Methylated-DNA--protein-cysteine methyltransferase (6-O-methylguanine-DNA methyltransferase) (MGMT) (O-6-methylguanine-DNA-alkyltransferase) | -4,29 |
| A0A0H2XFS5 | Acetyltransferase, GNAT family | -4,24 |
| A0A0H2XFZ5 | Putative membrane protein | -4,23 |
| A0A0H2XHL7 | Cytosolic protein | -4,19 |
| A0A0H2XK95 | Putative endoribonuclease L-PSP | -4,13 |
| holB | DNA polymerase III subunit delta' | -4,13 |
| estA | S-formylglutathione hydrolase | -4,12 |
| A0A0H2XEU1 | Acetyltransferase, GNAT family | -4,10 |
| A0A0H2XE84 | NTP pyrophosphohydrolase MazG-like domain-containing protein | -4,06 |
| A0A0H2XFP7 | Transcriptional regulator, MarR family | -4,05 |
| A0A0H2XKK6 | nitric oxide dioxygenase | -4,03 |
| ndk | Nucleoside diphosphate kinase (NDK) (NDP kinase) (Nucleoside-2-P kinase) | -4,02 |
| A0A0H2XH27 | Phage protein | -4,00 |
| A0A0H2XF46 | S1 motif domain-containing protein | -3,99 |
| A0A0H2XJX3 | Acetyltransferase, GNAT family family | -3,96 |
| A0A0H2XDN5 | Copper-sensing transcriptional repressor CsoR | -3,96 |
| ybaK | Cys-tRNA(Pro)/Cys-tRNA(Cys) deacylase | -3,95 |
| sodM | Superoxide dismutase [Mn/Fe] 2 | -3,94 |
| rpmC | Large ribosomal subunit protein uL29 | -3,94 |
| A0A0H2XG96 | Uncharacterized protein | -3,93 |
| sraP | Serine-rich adhesin for platelets (Adhesin SraP) (Staphylococcus aureus surface protein A) | -3,92 |
| mazF | Endoribonuclease MazF (Toxin MazF) (mRNA interferase MazF) | -3,88 |
| Q2FGL9 | Bacilliredoxin SAUSA300_1463 | -3,86 |
| rnmV | Ribonuclease M5 (RNase M5) (Ribosomal RNA terminal maturase M5) | -3,84 |
| A0A0H2XEP7 | Acetyltransferase family protein | -3,82 |
| narJ | Respiratory nitrate reductase, delta subunit | -3,80 |
| A0A0H2XG46 | Putative oxidoreductase | -3,78 |
| A0A0H2XII8 | Cold shock protein CspA | -3,76 |
| vga | ABC transporter, ATP-binding protein | -3,75 |
| nreB | Oxygen sensor histidine kinase NreB (Nitrogen regulation protein B) | -3,74 |
| A0A0H2XGX1 | RNA methyltransferase, TrmH family | -3,72 |
| A0A0H2XH88 | ABC transporter, ATP-binding protein | -3,70 |
| A0A0H2XGH6 | Tautomerase (EC 5.3.2.-) | -3,66 |
| esxA | Type VII secretion system extracellular protein A (Ess extracellular protein A) | -3,62 |
| A0A0H2XFV1 | HAD-superfamily hydrolase, subfamily IA, variant 1 | -3,60 |
| rpoY | DNA-directed RNA polymerase subunit epsilon (RNAP epsilon subunit) | -3,60 |
| A0A0H2XJW1 | Threonylcarbamoyl-AMP synthase (TC-AMP synthase) | -3,59 |
| fur | Ferric uptake regulation protein | -3,58 |
| rpsP | Small ribosomal subunit protein bS16 (30S ribosomal protein S16) | -3,55 |
| A0A0H2XHR2 | Protozoan/cyanobacterial globin family protein | -3,55 |
| Q2FJG3 | Nucleoid-associated protein SAUSA300_0453 | -3,54 |
| A0A0H2XDW9 | DinB-like domain-containing protein | -3,53 |
| lytR | Transcriptional regulatory protein LytR (Sensory transduction protein LytR) | -3,53 |
| A0A0H2XFU5 | NETI motif-containing protein | -3,51 |
| A0A0H2XJV3 | ABC transporter, ATP-binding protein | -3,51 |
| A0A0H2XHA6 | DUF4176 domain-containing protein | -3,50 |
| acpS | Holo-[acyl-carrier-protein] synthase (Holo-ACP synthase) | -3,47 |
| A0A0H2XGG3 | Hydrolase, haloacid dehalogenase-like family | -3,46 |
| nrdG | Anaerobic ribonucleoside-triphosphate reductase-activating protein | -3,45 |
| A0A0H2XJ71 | Kinase | -3,45 |
| A0A0H2XIS1 | D-isomer specific 2-hydroxyacid dehydrogenase | -3,44 |
| A0A0H2XH82 | Putative lipoprotein | -3,44 |
| A0A0H2XFW4 | Peptidase, U32 family | -3,43 |
| rpmJ | Large ribosomal subunit protein bL36 (50S ribosomal protein L36) | -3,42 |
| rplU | Large ribosomal subunit protein bL21 (50S ribosomal protein L21) | -3,42 |
| A0A0H2XFK7 | Transcriptional regulator, MarR family | -3,41 |
| A0A0H2XK08 | Oxidoreductase, short chain dehydrogenase/reductase family | -3,40 |
| oppF | Oligopeptide ABC transporter, ATP-binding protein | -3,40 |
| spoVG | Putative septation protein SpoVG | -3,38 |
| A0A0H2XHS5 | Putative thioredoxin | -3,36 |
| A0A0H2XI13 | DUF985 domain-containing protein | -3,34 |
| Q2FFL5 | UPF0435 protein SAUSA300_1861 | -3,32 |
| ipdC | Indole-3-pyruvate decarboxylase | -3,32 |
| A0A0H2XJG1 | Glyoxalase family protein | -3,31 |
| A0A0H2XIC7 | Putative Na+/H+ antiporter | -3,29 |
| A0A0H2XFT0 | HTH-type transcriptional regulator | -3,26 |
| A0A0H2XHV0 | Transcriptional regulator, MarR family | -3,23 |
| A0A0H2XDE4 | HTH-type transcriptional regulator MgrA | -3,22 |
| A0A0H2XGD8 | Aldo/keto reductase family protein | -3,22 |
| A0A0H2XJN3 | Glycolytic operon regulator | -3,22 |
| gatC | Aspartyl/glutamyl-tRNA(Asn/Gln) amidotransferase subunit C (Asp/Glu-ADT subunit C) | -3,21 |
| pheS | Phenylalanine--tRNA ligase alpha subunit | -3,20 |
| glnR | Glutamine synthetase repressor | -3,19 |
| A0A0H2XFU4 | TIGR01741 family protein | -3,19 |
| rpoZ | DNA-directed RNA polymerase subunit omega (RNAP omega subunit) | -3,18 |
| A0A0H2XH55 | Cold shock protein CspA | -3,18 |
| nnrD | ADP-dependent (S)-NAD(P)H-hydrate dehydratase (ADP-dependent NAD(P)HX dehydratase) | -3,17 |
| Q2FFY7 | Putative dipeptidase SAUSA300_1697 | -3,16 |
| isaB | Immunodominant staphylococcal antigen B | -3,16 |
| A0A0H2XHA3 | N-acetylmuramoyl-L-alanine amidase | -3,15 |
| A0A0H2XHR8 | DJ-1/PfpI domain-containing protein | -3,13 |
| A0A0H2XFA3 | Cyclase family protein | -3,10 |
| A0A0H2XE67 | Ribosomal protein L7Ae | -3,10 |
| ybeY | Endoribonuclease YbeY | -3,10 |
| A0A0H2XE63 | Putative transcriptional regulator | -3,08 |
| A0A0H2XED8 | YwdI family protein | -3,08 |
| A0A0H2XI88 | Transcriptional regulator, LysR family | -3,08 |
| A0A0H2XJ34 | DUF1806 family protein | -3,08 |
| clpL | ATP-dependent Clp protease ATP-binding subunit ClpL | -3,06 |
| A0A0H2XEC3 | Glutathione peroxidase | -3,04 |
| A0A0H2XE12 | DUF86 domain-containing protein | -3,03 |
| A0A0H2XKB6 | Cytosolic protein | -3,03 |
| A0A0H2XFK1 | Activator of Hsp90 ATPase homologue 1-like C-terminal domain-containing protein | -3,03 |
| rlmH | Ribosomal RNA large subunit methyltransferase H | -3,02 |
| nreC | Oxygen regulatory protein NreC (Nitrogen regulation protein C) | -3,01 |
| ptpB | Low molecular weight protein-tyrosine-phosphatase PtpB | -3,00 |
| A0A0H2XHP6 | Hydrolase, carbon-nitrogen family | -2,97 |
| tdk | Thymidine kinase | -2,96 |
| A0A0H2XJM3 | His repressor | -2,96 |
| A0A0H2XGR3 | Uncharacterized protein | -2,96 |
| A0A0H2XF36 | Uncharacterized protein | -2,95 |
| A0A0H2XGK6 | Cytosolic protein | -2,92 |
| A0A0H2XGT2 | Transcriptional regulator, Fur family | -2,90 |
| A0A0H2XGD6 | Thioesterase domain-containing protein | -2,89 |
| A0A0H2XHY2 | FAD/NAD(P)-binding Rossmann fold Superfamily | -2,89 |
| A0A0H2XH06 | DNA methylase adenine-specific domain-containing protein | -2,87 |
| thrS | Threonine--tRNA ligase (Threonyl-tRNA synthetase) (ThrRS) | -2,84 |
| A0A0H2XJA7 | isochorismate synthase | -2,84 |
| A0A0H2XHG2 | DUF1934 family protein | -2,83 |
| pepT | Peptidase T | -2,83 |
| Q2FG30 | Uncharacterized peptidase SAUSA300_1654 | -2,82 |
| rpoF | RNA polymerase sigma factor | -2,82 |
| rsbV | Anti-sigma factor antagonist | -2,81 |
| A0A0H2XIX6 | DUF2316 family protein | -2,81 |
| A0A0H2XF94 | Initiation-control protein YabA | -2,81 |
| mobB | Molybdopterin-guanine dinucleotide biosynthesis protein B | -2,80 |
| Q2FH10 | Bacilliredoxin SAUSA300_1321 | -2,79 |
| A0A0H2XI15 | Uncharacterized protein | -2,78 |
| pyrR | Bifunctional protein PyrR | -2,78 |
| A0A0H2XFW2 | UspA domain-containing protein | -2,77 |
| Q2FH83 | Uncharacterized protein SAUSA300_1248 | -2,77 |
| A0A0H2XEG5 | Polyribonucleotide nucleotidyltransferase | -2,75 |
| A0A0H2XH94 | ABC transporter permease protein | -2,74 |
| Q2FFH4 | Uncharacterized protein SAUSA300_1902 | -2,73 |
| nfrA | NADPH-dependent oxidoreductase | -2,73 |
| A0A0H2XHI3 | Inositol monophosphatase family protein | -2,73 |
| pcp | Pyrrolidone-carboxylate peptidase | -2,72 |
| A0A0H2XIX9 | Phage protein | -2,72 |
| ddh | D-lactate dehydrogenase | -2,72 |
| A0A0H2XIZ0 | OsmC/Ohr family protein | -2,72 |
| A0A0H2XI16 | Hydrolase, TatD family | -2,71 |
| Q2FIC1 | Putative peptidyl-prolyl cis-trans isomerase (PPIase) | -2,71 |
| A0A0H2XI41 | Inosine-uridine preferring nucleoside hydrolase | -2,70 |
| gcvH | Glycine cleavage system H protein (Octanoyl/lipoyl carrier protein) | -2,70 |
| Q2FGB0 | Putative pre-16S rRNA nuclease | -2,70 |
| A0A0H2XET3 | Lipoprotein | -2,69 |
| A0A0H2XG91 | Lactose phosphotransferase system repressor | -2,69 |
| Q2FE15 | Uncharacterized lipoprotein SAUSA300_2428/SAUSA300_2429 | -2,67 |
| A0A0H2XK06 | DNA polymerase beta | -2,67 |
| trkA | Potassium uptake protein | -2,67 |
| A0A0H2XEY5 | Uncharacterized protein | -2,67 |
| A0A0H2XHG1 | Flavin reductase like domain-containing protein | -2,66 |
| A0A0H2XI27 | Hydrolase, haloacid dehalogenase-like family | -2,65 |
| A0A0H2XGI5 | NADH-dependent flavin oxidoreductase | -2,65 |
| A0A0H2XIS0 | Uncharacterized protein | -2,65 |
| gmk | Guanylate kinase (GMP kinase) | -2,64 |
| Q2FEC8 | Uncharacterized lipoprotein SAUSA300_2315 | -2,61 |
| A0A0H2XGQ9 | DNA-binding response regulator, LuxR family | -2,61 |
| hslO | 33 kDa chaperonin (Heat shock protein 33 homolog) (HSP33) | -2,61 |
| A0A0H2XGN7 | Putative thioredoxin | -2,60 |
| A0A0H2XDD9 | Metallo-beta-lactamase domain-containing protein | -2,60 |
| A0A0H2XIQ1 | Putative TrmH family tRNA/rRNA methyltransferase | -2,59 |
| A0A0H2XGQ0 | YlbF family regulator | -2,59 |
| nagA | N-acetylglucosamine-6-phosphate deacetylase | -2,58 |
| leuS | Leucine--tRNA ligase (Leucyl-tRNA synthetase) (LeuRS) | -2,58 |
| A0A0H2XFR9 | YwpF protein | -2,57 |
| Q2FDS6 | Uncharacterized hydrolase SAUSA300_2518 | -2,57 |
| A0A0H2XI69 | Putative glutamyl aminopeptidase | -2,55 |
| A0A0H2XDT0 | Nitroreductase family protein | -2,55 |
| A0A0H2XH96 | DUF2750 domain-containing protein | -2,55 |
| A0A0H2XHK4 | TIGR04141 family sporadically distributed protein | -2,54 |
| A0A0H2XIG7 | TIGR00282 family metallophosphoesterase | -2,54 |
| adhE | Aldehyde-alcohol dehydrogenase | -2,54 |
| A0A0H2XHJ3 | Acetyltransferase, GNAT family | -2,52 |
| ruvB | Holliday junction branch migration complex subunit RuvB | -2,52 |
| rpsJ | Small ribosomal subunit protein uS10 (30S ribosomal protein S10) | -2,51 |
| msrA | Peptide methionine sulfoxide reductase MsrA (Protein-methionine-S-oxide reductase) (Peptide-methionine (S)-S-oxide reductase) (Peptide Met(O) reductase) | -2,50 |
| A0A0H2XH61 | HD domain protein | -2,49 |
| pcrA | ATP-dependent DNA helicase | -2,49 |
| A0A0H2XGI9 | MutT/nudix family protein | -2,49 |
| tyrS | Tyrosine--tRNA ligase (Tyrosyl-tRNA synthetase) (TyrRS) | -2,48 |
| A0A0H2XG57 | Thiamine-binding protein domain-containing protein | -2,48 |
| A0A0H2XI23 | DegV family protein | -2,48 |
| nagD | Acid sugar phosphatase | -2,48 |
| A0A0H2XHJ8 | Peptidase, M16 family | -2,47 |
| tag | DNA-3-methyladenine glycosidase | -2,46 |
| A0A0H2XG78 | D-isomer specific 2-hydroxyacid dehydrogenase family protein | -2,46 |
| A0A0H2XE66 | NfeD-like C-terminal domain-containing protein | -2,46 |
| modC | Molybdenum ABC transporter, ATP-binding protein ModC | -2,45 |
| nth | Endonuclease III (DNA-(apurinic or apyrimidinic site) lyase) | -2,43 |
| sceD | Probable transglycosylase SceD | -2,43 |
| A0A0H2XHY3 | Glycerate kinase | -2,42 |
| A0A0H2XH57 | HIT family protein | -2,42 |
| cvfB | Conserved virulence factor B | -2,42 |
| ahpC | Alkyl hydroperoxide reductase C (Peroxiredoxin) (Thioredoxin peroxidase) | -2,42 |
| A0A0H2XH22 | Putative glycerophosphoryl diester phosphodiesterase | -2,42 |
| A0A0H2XFY7 | Uncharacterized protein | -2,41 |
| A0A0H2XJ90 | D-lactate dehydrogenase | -2,41 |
| A0A0H2XIM6 | Uncharacterized protein | -2,40 |
| def | Peptide deformylase (PDF) | -2,40 |
| A0A0H2XFZ0 | DNA repair/chromosome segregation ATPase | -2,40 |
| A0A0H2XHS7 | thioredoxin-dependent peroxiredoxin | -2,38 |
| A0A0H2XJC0 | SUF system FeS assembly protein, NifU family | -2,38 |
| Q2FDY2 | Putative NAD(P)H nitroreductase SAUSA300_2462 | -2,37 |
| A0A0H2XJ54 | HD domain-containing protein | -2,37 |
| gcvPB | Probable glycine dehydrogenase (decarboxylating) subunit 2 | -2,37 |
| ureG | Urease accessory protein UreG | -2,37 |
| A0A0H2XEN4 | Glutaredoxin domain-containing protein | -2,37 |
| A0A0H2XJP7 | Hydrolase, haloacid dehalogenase-like family | -2,36 |
| A0A0H2XH98 | 8-oxo-dGTP diphosphatase | -2,36 |
| A0A0H2XE07 | Phage protein | -2,34 |
| mraZ | Transcriptional regulator MraZ | -2,34 |
| A0A0H2XJT5 | Acetyltransferase, GNAT family | -2,34 |
| oppA | Oligopeptide ABC transporter, substrate-binding protein | -2,34 |
| A0A0H2XH20 | Protein from nitrogen regulatory protein P-II (GLNB) family | -2,33 |
| rplW | Large ribosomal subunit protein uL23 (50S ribosomal protein L23) | -2,33 |
| A0A0H2XJK0 | Aminotransferase | -2,33 |
| Q2FFQ0 | UPF0342 protein SAUSA300_1795 | -2,31 |
| cap5B | non-specific protein-tyrosine kinase | -2,31 |
| A0A0H2XGA2 | DUF488 domain-containing protein | -2,30 |
| A0A0H2XFT5 | DNA-binding response regulator | -2,29 |
| Q2FGA9 | UPF0297 protein SAUSA300_1574 | -2,29 |
| narH | Respiratory nitrate reductase, beta subunit | -2,27 |
| A0A0H2XFI2 | BD-FAE-like domain-containing protein | -2,27 |
| A0A0H2XFX6 | Uncharacterized protein | -2,27 |
| A0A0H2XK62 | YbhB/YbcL family Raf kinase inhibitor-like protein | -2,27 |
| A0A0H2XGJ5 | Transcriptional regulator | -2,27 |
| A0A0H2XE45 | UDP-N-acetylglucosamine 2-epimerase (non-hydrolyzing) | -2,26 |
| A0A0H2XET4 | Transaldolase | -2,26 |
| fhuG | Ferrichrome transport permease protein fhuG | -2,25 |
| A0A0H2XK09 | Type II NADH:quinone oxidoreductase | -2,25 |
| walR | Transcriptional regulatory protein WalR | -2,25 |
| A0A0H2XJG5 | Alpha/beta hydrolase fold-3 domain-containing protein | -2,24 |
| A0A0H2XIQ9 | Uncharacterized protein | -2,24 |
| A0A0H2XK17 | Putative helicase | -2,24 |
| glmM | Phosphoglucosamine mutase | -2,23 |
| map | Methionine aminopeptidase (MAP) (MetAP) (Peptidase M) | -2,22 |
| A0A0H2XI75 | Transcriptional regulator, LysR family | -2,22 |
| ampA | Cytosol aminopeptidase | -2,21 |
| A0A0H2XFE1 | PepSY domain-containing protein | -2,21 |
| A0A0H2XH76 | Uncharacterized protein | -2,20 |
| A0A0H2XFJ9 | Peptidase, M20/M25/M40 family | -2,20 |
| A0A0H2XH25 | TIGR01741 family protein | -2,19 |
| A0A0H2XH60 | uroporphyrinogen-III C-methyltransferase | -2,19 |
| A0A0H2XGS6 | TIGR01741 family protein | -2,18 |
| isaA | Probable transglycosylase IsaA (Immunodominant staphylococcal antigen A) | -2,17 |
| A0A0H2XF90 | Geranyltranstransferase | -2,17 |
| A0A0H2XGA1 | Dehydrogenase family protein | -2,17 |
| A0A0H2XGV3 | Acetyltransferase, GNAT family | -2,17 |
| Q2FDH4 | UPF0312 protein SAUSA300_2620 | -2,16 |
| rsbW | Serine-protein kinase RsbW (Anti-sigma-B factor) (Sigma-B negative effector RsbW) | -2,15 |
| A0A0H2XG61 | thiamine diphosphokinase | -2,14 |
| ahpF | Alkyl hydroperoxide reductase subunit F | -2,14 |
| hup | DNA-binding protein HU | -2,14 |
| A0A0H2XFJ4 | Gcp-like domain-containing protein | -2,14 |
| A0A0H2XIA0 | Genomic island nu Sa alpha2 | -2,13 |
| engB | Probable GTP-binding protein EngB | -2,13 |
| Q2FES9 | Uncharacterized hydrolase SAUSA300_2163 | -2,13 |
| trmD | tRNA (guanine-N(1)-)-methyltransferase (M1G-methyltransferase) (tRNA [GM37] methyltransferase) | -2,12 |
| A0A0H2XJP4 | Putative thioredoxin | -2,11 |
| acsA | Putative long chain fatty acid-CoA ligase VraA | -2,10 |
| mutM | Formamidopyrimidine-DNA glycosylase | -2,10 |
| hprK | HPr kinase/phosphorylase (HPrK/P) | -2,10 |
| fmt | Methionyl-tRNA formyltransferase | -2,08 |
| rpsA | Small ribosomal subunit protein bS1 (30S ribosomal protein S1) | -2,08 |
| A0A0H2XIM7 | Scaffold protein Nfu/NifU N-terminal domain-containing protein | -2,08 |
| dnaG | DNA primase | -2,08 |
| atpF | ATP synthase subunit b (ATP synthase F(0) sector subunit b) | -2,07 |
| rpmE2 | Large ribosomal subunit protein bL31B (50S ribosomal protein L31 type B) | -2,07 |
| trpS | Tryptophan--tRNA ligase (Tryptophanyl-tRNA synthetase) (TrpRS) | -2,07 |
| dnaX | DNA polymerase III subunit gamma/tau | -2,07 |
| nusB | Transcription antitermination protein NusB (Antitermination factor NusB) | -2,07 |
| A0A0H2XFG3 | ComE operon protein 2 | -2,07 |
| A0A0H2XI33 | 5' nucleotidase family protein | -2,07 |
| A0A0H2XH40 | Uncharacterized protein | -2,06 |
| A0A0H2XF54 | Bacterial luciferase family protein | -2,06 |
| A0A0H2XG68 | Phi77 ORF011-like protein, phage transcriptional repressor | -2,06 |
| A0A0H2XJ55 | General stress protein 17M-like domain-containing protein | -2,05 |
| ptsH | Phosphocarrier protein HPr (Histidine-containing protein) | -2,05 |
| A0A0H2XEJ0 | Uncharacterized protein | -2,05 |
| A0A0H2XEY2 | Thioredoxin family protein | -2,05 |
| A0A0H2XED7 | DegV family protein | -2,05 |
| rplN | Large ribosomal subunit protein uL14 (50S ribosomal protein L14) | -2,04 |
| A0A0H2XGP2 | Disulfide oxidoreductase | -2,04 |
| lpdA | Dihydrolipoyl dehydrogenase | -2,04 |
| rsbU | Sigma-B regulation protein | -2,03 |
| A0A0H2XHV1 | Bacterial transcription activator effector binding domain-containing protein | -2,03 |
| A0A0H2XKJ0 | Cof-type HAD-IIB family hydrolase | -2,03 |
| ilvN | acetolactate synthase | -2,02 |
| rpmD | Large ribosomal subunit protein uL30 (50S ribosomal protein L30) | -2,01 |
| A0A0H2XGT0 | SIS domain protein | -2,01 |
| est | Carboxylesterase | -2,01 |
| uvrA | UvrABC system protein A (UvrA protein) (Excinuclease ABC subunit A) | -2,00 |
| A0A0H2XHJ1 | Acetyltransferase, GNAT family | -2,00 |

| **Table S4 Upregulated genes with no assigned pathway in 880 under Moxifloxacin treatment** | | |
| --- | --- | --- |
| **Gene Name** | **Protein name** | **log2 Fold Change (FC)** |
| ssaA | Secretory antigen SsaA | 7,15 |
| A0A0H2XGB2 | Uncharacterized protein | 6,60 |
| A0A0H2XI01 | N-acetylmuramoyl-L-alanine amidase domain protein | 5,82 |
| A0A0H2XIX8 | Putative lipoprotein | 5,57 |
| ssaA | Secretory antigen SsaA | 5,27 |
| A0A0H2XI20 | DUF2273 domain-containing protein | 4,60 |
| sbcC | Nuclease SbcCD subunit C | 4,56 |
| A0A0H2XEH7 | Staphopain A | 4,54 |
| A0A0H2XG38 | Phage protein | 4,40 |
| lytM | Glycyl-glycine endopeptidase LytM | 4,22 |
| atl | Bifunctional autolysin | 4,15 |
| opuD | Glycine betaine transporter opuD | 3,98 |
| A0A0H2XFV0 | Isoprenylcysteine carboxyl methyltransferase family protein | 3,65 |
| A0A0H2XFT2 | UPF0154 protein SAUSA300_1240 | 3,55 |
| A0A0H2XJG9 | Maebl | 3,50 |
| yajC | Preprotein translocase, YajC subunit | 3,39 |
| pknB | non-specific serine/threonine protein kinase | 3,38 |
| smpB | SsrA-binding protein (Small protein B) | 3,15 |
| A0A0H2XIC9 | Putative teichoic acid biosynthesis protein | 2,92 |
| Q2FFI4 | UPF0316 protein SAUSA300_1892 | 2,84 |
| A0A0H2XFD4 | DUF4889 domain-containing protein | 2,80 |
| sle1 (aaa) | N-acetylmuramoyl-L-alanine amidase sle1 | 2,79 |
| A0A0H2XG53 | Uncharacterized protein | 2,78 |
| Q2FFA2 | Uncharacterized leukocidin-like protein 2 | 2,77 |
| A0A0H2XJA4 | Cytochrome D ubiquinol oxidase, subunit II | 2,76 |
| sceD | Probable transglycosylase SceD | 2,73 |
| rpsR | Small ribosomal subunit protein bS18 (30S ribosomal protein S18) | 2,72 |
| A0A0H2XE91 | DUF3139 domain-containing protein | 2,71 |
| A0A0H2XIN7 | Chorismate binding enzyme domain protein | 2,69 |
| A0A0H2XJ17 | Amino acid permease | 2,68 |
| A0A0H2XHY6 | Urea amidolyase-related protein | 2,66 |
| A0A0H2XFG6 | YtxH domain-containing protein | 2,57 |
| treP | PTS system, trehalose-specific IIBC component | 2,55 |
| A0A0H2XJL8 | Polysaccharide biosynthesis protein | 2,52 |
| A0A0H2XJU8 | Lytic regulatory protein | 2,51 |
| Q2FJ50 | UPF0741 protein SAUSA300_0575 | 2,39 |
| topA | DNA topoisomerase 1 | 2,38 |
| A0A0H2XHF6 | Secretory antigen SsaA | 2,35 |
| pyc | Pyruvate carboxylase | 2,29 |
| A0A0H2XJQ0 | DNA2/NAM7 helicase-like C-terminal domain-containing protein | 2,28 |
| A0A0H2XIB2 | Uncharacterized protein | 2,26 |
| A0A0H2XHX8 | Putative membrane protein | 2,24 |
| Q2FFZ9 | UPF0478 protein SAUSA300_1685 | 2,22 |
| A0A0H2XFB1 | Uncharacterized protein | 2,21 |
| ezrA | Septation ring formation regulator EzrA | 2,21 |
| ermC | rRNA adenine N-6-methyltransferase | 2,19 |
| A0A0H2XHX2 | Putative membrane protein | 2,17 |
| A0A0H2XE61 | ABC transporter, ATP-binding protein | 2,15 |
| secF (secD) | Multifunctional fusion protein [Includes: Protein translocase subunit SecD; Protein-export membrane protein SecF] | 2,11 |
| A0A0H2XI39 | Alkaline shock response membrane anchor protein AmaP | 2,10 |
| rplT | Large ribosomal subunit protein bL20 (50S ribosomal protein L20) | 2,09 |
| A0A0H2XE83 | PTS system, sucrose-specific IIBC component | 2,09 |
| A0A0H2XJF7 | Putative membrane protein | 2,06 |
| A0A0H2XFZ6 | Transcriptional regulator | 2,05 |
| A0A0H2XH45 | Putative lipoprotein | 2,05 |
| A0A0H2XE66 | NfeD-like C-terminal domain-containing protein | 2,01 |
| A0A0H2XIL7 | PhiSLT ORF86-like protein | 2,00 |
| **Downregulated genes with no assigned pathway in 880 under Moxifloxacin treatment** | | |
| **Gene Name** | **Protein name** | **log2 Fold Change (FC)** |
| A0A0H2XIX9 | Phage protein | -11,57 |
| A0A0H2XH27 | Phage protein | -9,40 |
| A0A0H2XGH6 | Tautomerase | -8,86 |
| Q2FFQ0 | UPF0342 protein SAUSA300_1795 | -8,48 |
| A0A0H2XI35 | Phage protein | -8,42 |
| Q2FGA9 | UPF0297 protein SAUSA300_1574 | -7,93 |
| A0A0H2XFV1 | HAD-superfamily hydrolase, subfamily IA, variant 1 | -7,85 |
| Q2FGL9 | Bacilliredoxin SAUSA300_1463 | -7,40 |
| A0A0H2XJ71 | Kinase | -7,30 |
| A0A0H2XIY3 | HAD-superfamily hydrolase, subfamily IA, variant 1 | -6,90 |
| A0A0H2XE88 | YpoC-like domain-containing protein | -6,59 |
| A0A0H2XK62 | YbhB/YbcL family Raf kinase inhibitor-like protein | -6,53 |
| A0A0H2XG13 | Acetyltransferase, GNAT family | -6,38 |
| A0A0H2XGJ5 | Transcriptional regulator | -6,34 |
| rsbV | Anti-sigma factor antagonist | -6,29 |
| A0A0H2XH76 | Uncharacterized protein | -6,16 |
| A0A0H2XE76 | Glyoxalase family protein | -5,99 |
| A0A0H2XFW8 | Methylated-DNA--protein-cysteine methyltransferase | -5,86 |
| A0A0H2XJP4 | Putative thioredoxin | -5,75 |
| Q2FJJ6 | Uncharacterized lipoprotein SAUSA300_0419 | -5,72 |
| rlmH | Ribosomal RNA large subunit methyltransferase H | -5,59 |
| A0A0H2XF46 | S1 motif domain-containing protein | -5,57 |
| ybeY | Endoribonuclease YbeY | -5,56 |
| A0A0H2XHP6 | Hydrolase, carbon-nitrogen family | -5,48 |
| A0A0H2XGN7 | Putative thioredoxin | -5,47 |
| mtlA | Mannitol-specific phosphotransferase enzyme IIA component (EIIA) | -5,38 |
| A0A0H2XGV3 | Acetyltransferase, GNAT family | -5,37 |
| sufT | Fe-S protein maturation auxiliary factor SufT | -5,37 |
| A0A0H2XDN5 | Copper-sensing transcriptional repressor CsoR | -5,36 |
| A0A0H2XI29 | Acetyltransferase | -5,36 |
| oppD | Oligopeptide ABC transporter, ATP-binding protein | -5,32 |
| A0A0H2XFR9 | YwpF protein | -5,30 |
| A0A0H2XHZ2 | TfoX N-terminal domain-containing protein | -5,27 |
| A0A0H2XHS5 | Putative thioredoxin | -5,20 |
| A0A0H2XE07 | Phage protein | -5,18 |
| cvfC | Conserved virulence factor C | -5,16 |
| A0A0H2XJ54 | HD domain-containing protein | -5,12 |
| A0A0H2XFP7 | Transcriptional regulator, MarR family | -5,01 |
| A0A0H2XFI2 | BD-FAE-like domain-containing protein | -5,00 |
| A0A0H2XEU5 | Excinuclease ABC subunit B | -5,00 |
| mraZ | Transcriptional regulator MraZ | -4,85 |
| Q2FIF3 | Probable nitronate monooxygenase (NMO) (Propionate 3-nitronate monooxygenase) (P3N monooxygenase) | -4,83 |
| A0A0H2XG57 | Thiamine-binding protein domain-containing protein | -4,82 |
| A0A0H2XFD8 | Na+-translocating membrane potential-generating system MpsC domain-containing protein | -4,81 |
| graR | Response regulator protein GraR (Glycopeptide resistance-associated protein R) | -4,79 |
| A0A0H2XH30 | Peptidase, M20/M25/M40 family | -4,79 |
| A0A0H2XH89 | DNA-binding protein | -4,79 |
| msrB | Peptide methionine sulfoxide reductase MsrB (Peptide-methionine (R)-S-oxide reductase) | -4,75 |
| esxA | Type VII secretion system extracellular protein A (Ess extracellular protein A) | -4,68 |
| A0A0H2XFM9 | DUF2197 domain-containing protein | -4,68 |
| A0A0H2XGF0 | HAD family hydrolase | -4,66 |
| A0A0H2XHM9 | Cytosolic protein | -4,64 |
| scdA | Iron-sulfur cluster repair protein ScdA | -4,61 |
| A0A0H2XH25 | TIGR01741 family protein | -4,58 |
| Q2FH17 | UPF0346 protein SAUSA300_1314 | -4,56 |
| A0A0H2XF74 | Putative phage infection protein | -4,53 |
| A0A0H2XK60 | Uncharacterized protein | -4,53 |
| A0A0H2XIT3 | Diacetyl reductase [(S)-acetoin forming] | -4,52 |
| A0A0H2XH57 | HIT family protein | -4,50 |
| A0A0H2XEP1 | Uncharacterized protein | -4,50 |
| rot | HTH-type transcriptional regulator rot (Repressor of toxins) | -4,45 |
| recX | Regulatory protein RecX | -4,44 |
| mcsB | Protein-arginine kinase | -4,42 |
| A0A0H2XGC0 | Putative adenylate cyclase | -4,41 |
| A0A0H2XIB6 | ABC transporter, ATP-binding protein | -4,39 |
| A0A0H2XHV0 | Transcriptional regulator, MarR family | -4,37 |
| A0A0H2XIX6 | DUF2316 family protein | -4,37 |
| A0A0H2XH03 | YozC | -4,36 |
| A0A0H2XG12 | MaoC-like domain-containing protein | -4,33 |
| A0A0H2XFR6 | GntR family regulatory protein | -4,30 |
| ctsR | Transcriptional regulator CtsR | -4,30 |
| Q2FH83 | Uncharacterized protein SAUSA300_1248 | -4,30 |
| A0A0H2XIK5 | Uncharacterized protein | -4,30 |
| A0A0H2XHW3 | Uncharacterized protein | -4,29 |
| A0A0H2XFD5 | Transcriptional regulator, LysR family domain protein | -4,28 |
| A0A0H2XH81 | Heat shock protein | -4,28 |
| A0A0H2XFA3 | Cyclase family protein | -4,27 |
| A0A0H2XH20 | Protein from nitrogen regulatory protein P-II (GLNB) family | -4,25 |
| nrdD | Anaerobic ribonucleotide reductase, large subunit | -4,24 |
| A0A0H2XGP2 | Disulfide oxidoreductase | -4,23 |
| A0A0H2XIZ0 | OsmC/Ohr family protein | -4,23 |
| A0A0H2XIS0 | Uncharacterized protein | -4,23 |
| opuCa | Glycine betaine/carnitine/choline ABC transporter ATP-binding protein | -4,22 |
| A0A0H2XEP7 | Acetyltransferase family protein | -4,22 |
| A0A0H2XGJ8 | Uncharacterized protein | -4,21 |
| A0A0H2XIF0 | MutT/nudix family protein | -4,20 |
| A0A0H2XGU3 | Uncharacterized protein | -4,18 |
| tilS | tRNA(Ile)-lysidine synthase | -4,18 |
| A0A0H2XEC3 | Glutathione peroxidase | -4,14 |
| A0A0H2XHS7 | thioredoxin-dependent peroxiredoxin | -4,14 |
| A0A0H2XJB0 | Transcriptional regulator, AraC family | -4,12 |
| infA | Translation initiation factor IF-1 | -4,08 |
| A0A0H2XJ32 | DNA internalization-related competence protein ComEC/Rec2 | -4,06 |
| A0A0H2XE84 | NTP pyrophosphohydrolase MazG-like domain-containing protein | -4,04 |
| rpsA | Small ribosomal subunit protein bS1 (30S ribosomal protein S1) | -4,04 |
| hpf | Ribosome hibernation promotion factor (HPF) (Ribosome hibernation-promoting factor) | -4,03 |
| metN2 | Methionine import ATP-binding protein MetN 2 | -4,01 |
| A0A0H2XE18 | Putative tRNA (cytidine(34)-2'-O)-methyltransferase | -4,01 |
| gatC | Aspartyl/glutamyl-tRNA(Asn/Gln) amidotransferase subunit C (Asp/Glu-ADT subunit C) | -4,00 |
| A0A0H2XGD6 | Thioesterase domain-containing protein | -3,99 |
| crr | PTS system glucose-specific EIIA component (EIIA-Glc) | -3,98 |
| pyrR | Bifunctional protein PyrR [Includes: Pyrimidine operon regulatory protein; Uracil phosphoribosyltransferase (UPRTase) | -3,94 |
| A0A0H2XHF8 | DUF961 domain-containing protein | -3,93 |
| A0A0H2XHA6 | DUF4176 domain-containing protein | -3,93 |
| oppA | Oligopeptide ABC transporter, substrate-binding protein | -3,91 |
| azo1 | FMN-dependent NADPH-azoreductase | -3,90 |
| A0A0H2XG91 | Lactose phosphotransferase system repressor | -3,89 |
| A0A0H2XFK1 | Activator of Hsp90 ATPase homologue 1-like C-terminal domain-containing protein | -3,89 |
| msrA | Peptide methionine sulfoxide reductase MsrA (Protein-methionine-S-oxide reductase) | -3,89 |
| A0A0H2XFL7 | Putative hemin transport system permease protein HrtB | -3,88 |
| Q2FIC1 | Putative peptidyl-prolyl cis-trans isomerase (PPIase) (Rotamase) | -3,88 |
| A0A0H2XFJ6 | Aldo/keto reductase family protein | -3,86 |
| gcvT | Aminomethyltransferase (Glycine cleavage system T protein) | -3,85 |
| A0A0H2XHV3 | Cell division protein ZapA (Z ring-associated protein ZapA) | -3,83 |
| A0A0H2XJN3 | Glycolytic operon regulator | -3,80 |
| A0A0H2XGZ2 | Uncharacterized protein | -3,78 |
| Q2FIA7 | Uncharacterized protein SAUSA300_0871 | -3,78 |
| hup | DNA-binding protein HU | -3,76 |
| Q2FGB1 | UPF0473 protein SAUSA300_1572 | -3,74 |
| A0A0H2XJ60 | Putative arsenate reductase | -3,74 |
| Q2FE21 | Uncharacterized oxidoreductase SAUSA300_2422 | -3,74 |
| nirD | Nitrite reductase [NAD(P)H], small subunit | -3,74 |
| A0A0H2XDZ0 | NIF system FeS cluster assembly NifU C-terminal domain-containing protein | -3,74 |
| A0A0H2XGT2 | Transcriptional regulator, Fur family | -3,74 |
| oppF | Oligopeptide ABC transporter, ATP-binding protein | -3,73 |
| cdd | Cytidine deaminase | -3,73 |
| pxpA | 5-oxoprolinase subunit A (5-OPase subunit A) | -3,73 |
| A0A0H2XFY7 | Uncharacterized protein | -3,72 |
| luxS | S-ribosylhomocysteine lyase (AI-2 synthesis protein) (Autoinducer-2 production protein LuxS) | -3,72 |
| A0A0H2XHF9 | Putative cell wall enzyme EbsB | -3,72 |
| A0A0H2XEA1 | Lipoprotein | -3,72 |
| A0A0H2XGV6 | YigZ family protein | -3,70 |
| grpE | Protein GrpE (HSP-70 cofactor) | -3,70 |
| A0A0H2XH98 | 8-oxo-dGTP diphosphatase | -3,68 |
| rsmI | Ribosomal RNA small subunit methyltransferase I | -3,68 |
| A0A0H2XGQ9 | DNA-binding response regulator, LuxR family | -3,66 |
| cobB | NAD-dependent protein deacetylase | -3,66 |
| A0A0H2XF90 | Geranyltranstransferase | -3,63 |
| A0A0H2XJ11 | Phage protein | -3,63 |
| nreC | Oxygen regulatory protein NreC (Nitrogen regulation protein C) | -3,62 |
| rbfA | Ribosome-binding factor A | -3,61 |
| A0A0H2XE31 | Antitoxin | -3,61 |
| sodA | Superoxide dismutase [Mn/Fe] 1 | -3,61 |
| A0A0H2XJX3 | Acetyltransferase, GNAT family family | -3,60 |
| mazF | Endoribonuclease MazF (Toxin MazF) (mRNA interferase MazF) | -3,58 |
| A0A0H2XHP5 | DNA-binding response regulator | -3,57 |
| A0A0H2XFU4 | TIGR01741 family protein | -3,55 |
| A0A0H2XJH8 | Peptidase M20 domain-containing protein 2 | -3,55 |
| A0A0H2XFW0 | Pyruvate ferredoxin oxidoreductase, beta subunit | -3,52 |
| ureE | Urease accessory protein UreE | -3,49 |
| A0A0H2XI13 | DUF985 domain-containing protein | -3,49 |
| A0A0H2XH33 | PhnB-like domain-containing protein | -3,47 |
| nagD | Acid sugar phosphatase | -3,47 |
| A0A0H2XFX8 | LSM domain protein | -3,47 |
| A0A0H2XGD0 | PhnB-like domain-containing protein | -3,45 |
| A0A0H2XE12 | DUF86 domain-containing protein | -3,45 |
| arlR | Response regulator ArlR | -3,45 |
| A0A0H2XJ55 | General stress protein 17M-like domain-containing protein | -3,42 |
| A0A0H2XJQ4 | Acetyltransferase, GNAT family | -3,42 |
| ptpA | Low molecular weight protein-tyrosine-phosphatase PtpA (Phosphotyrosine phosphatase A) (PTPase A) | -3,41 |
| A0A0H2XKK6 | nitric oxide dioxygenase | -3,41 |
| A0A0H2XH55 | Cold shock protein CspA | -3,41 |
| A0A0H2XHR2 | Protozoan/cyanobacterial globin family protein | -3,40 |
| A0A0H2XE20 | Uncharacterized protein | -3,39 |
| walR | Transcriptional regulatory protein WalR | -3,39 |
| A0A0H2XHJ3 | Acetyltransferase, GNAT family | -3,39 |
| A0A0H2XEJ0 | Uncharacterized protein | -3,38 |
| A0A0H2XEM0 | Flavin reductase like domain-containing protein | -3,37 |
| A0A0H2XI88 | Transcriptional regulator, LysR family | -3,37 |
| deoD | Purine nucleoside phosphorylase DeoD-type (PNP) | -3,36 |
| sarX | HTH-type transcriptional regulator SarX (Staphylococcal accessory regulator X) | -3,35 |
| A0A0H2XHB3 | Purine nucleoside phosphorylase | -3,34 |
| A0A0H2XDW9 | DinB-like domain-containing protein | -3,34 |
| Q2FES9 | Uncharacterized hydrolase SAUSA300_2163 | -3,34 |
| A0A0H2XHN9 | Thiamine-binding protein domain-containing protein | -3,33 |
| tag | DNA-3-methyladenine glycosidase | -3,32 |
| cmk | (d)CMP kinase | -3,31 |
| pflA | Pyruvate formate-lyase-activating enzyme (PFL-activating enzyme) | -3,31 |
| clpL | ATP-dependent Clp protease ATP-binding subunit ClpL | -3,30 |
| A0A0H2XII8 | Cold shock protein CspA | -3,30 |
| A0A0H2XGT0 | SIS domain protein | -3,30 |
| hchA | Protein/nucleic acid deglycase HchA | -3,30 |
| A0A0H2XJ33 | Uncharacterized protein | -3,30 |
| A0A0H2XFJ9 | Peptidase, M20/M25/M40 family | -3,29 |
| A0A0H2XHU8 | Bacillithiol system redox-active protein YtxJ | -3,29 |
| phoP | Alkaline phosphatase synthesis transcriptional regulatory protein PhoP | -3,27 |
| A0A0H2XIQ1 | Putative TrmH family tRNA/rRNA methyltransferase | -3,27 |
| A0A0H2XI75 | Transcriptional regulator, LysR family | -3,27 |
| A0A0H2XHN2 | Haloacid dehalogenase-like hydrolase | -3,26 |
| A0A0H2XHW2 | Antigen, 67 kDa | -3,25 |
| Q2FH40 | Uncharacterized hydrolase SAUSA300_1291 | -3,24 |
| A0A0H2XHL7 | Cytosolic protein | -3,24 |
| A0A0H2XFU5 | NETI motif-containing protein | -3,24 |
| atpC | ATP synthase epsilon chain (ATP synthase F1 sector epsilon subunit) | -3,24 |
| A0A0H2XJA6 | TIGR01741 family protein | -3,21 |
| A0A0H2XGW9 | Deoxynucleoside kinase family protein | -3,21 |
| A0A0H2XG46 | Putative oxidoreductase | -3,20 |
| A0A0H2XIY8 | Oxidoreductase, aldo/keto reductase family | -3,20 |
| Q2FDY2 | Putative NAD(P)H nitroreductase SAUSA300_2462 | -3,20 |
| A0A0H2XIC8 | Staphylococcal protein | -3,19 |
| sarZ | HTH-type transcriptional regulator SarZ (Staphylococcal accessory regulator Z) | -3,18 |
| A0A0H2XEL6 | Uncharacterized protein | -3,17 |
| nrdG | Anaerobic ribonucleoside-triphosphate reductase-activating protein | -3,17 |
| esaA | Type VII secretion system accessory factor EsaA | -3,17 |
| A0A0H2XGG3 | Hydrolase, haloacid dehalogenase-like family | -3,15 |
| Q2FI62 | Putative phosphoesterase SAUSA300_0916 | -3,15 |
| rplI | Large ribosomal subunit protein bL9 (50S ribosomal protein L9) | -3,14 |
| clpC | ATP-dependent Clp protease ATP-binding subunit ClpC | -3,14 |
| A0A0H2XHU7 | DUF177 domain-containing protein | -3,14 |
| A0A0H2XEY2 | Thioredoxin family protein | -3,13 |
| A0A0H2XKB1 | DUF1641 domain-containing protein | -3,13 |
| A0A0H2XGK6 | Cytosolic protein | -3,12 |
| A0A0H2XJ48 | Rrf2 family transcriptional regulator | -3,12 |
| A0A0H2XFY9 | Aminotransferase | -3,11 |
| A0A0H2XIM7 | Scaffold protein Nfu/NifU N-terminal domain-containing protein | -3,11 |
| A0A0H2XHE8 | MutT/nudix family protein | -3,11 |
| clpP | ATP-dependent Clp protease proteolytic subunit (Endopeptidase Clp) | -3,10 |
| A0A0H2XHE7 | Rhodanese-like domain protein | -3,10 |
| A0A0H2XHG1 | Flavin reductase like domain-containing protein | -3,09 |
| A0A0H2XKD5 | GAF domain-containing protein | -3,09 |
| A0A0H2XJM3 | His repressor | -3,09 |
| A0A0H2XF54 | Bacterial luciferase family protein | -3,07 |
| trxB | Thioredoxin reductase | -3,04 |
| Q2FGB0 | Putative pre-16S rRNA nuclease | -3,04 |
| A0A0H2XF92 | NYN domain-containing protein | -3,03 |
| A0A0H2XH61 | HD domain protein | -3,02 |
| tetR | Biofilm operon icaADBC HTH-type negative transcriptional regulator IcaR (Intercellular adhesion protein R) | -3,01 |
| adh | Alcohol dehydrogenase (ADH) | -3,01 |
| A0A0H2XJ90 | D-lactate dehydrogenase | -3,01 |
| nreB | Oxygen sensor histidine kinase NreB (Nitrogen regulation protein B) | -2,99 |
| A0A0H2XIS5 | Kinase, pfkB family | -2,97 |
| nagA | N-acetylglucosamine-6-phosphate deacetylase | -2,95 |
| Q2FG30 | Uncharacterized peptidase SAUSA300_1654 | -2,95 |
| A0A0H2XG78 | D-isomer specific 2-hydroxyacid dehydrogenase family protein | -2,93 |
| A0A0H2XK09 | Type II NADH:quinone oxidoreductase | -2,92 |
| tpx | Thiol peroxidase (Tpx) | -2,92 |
| holB | DNA polymerase III subunit delta' | -2,92 |
| A0A0H2XFT0 | HTH-type transcriptional regulator | -2,91 |
| A0A0H2XKD6 | TIGR01741 family protein | -2,90 |
| A0A0H2XI15 | Uncharacterized protein | -2,90 |
| A0A0H2XFA8 | Aminotransferase | -2,89 |
| A0A0H2XFM1 | Putative DNA replication protein DnaD | -2,87 |
| perR | Peroxide-responsive repressor PerR | -2,87 |
| Q2FHK2 | UPF0122 protein SAUSA300_1129 | -2,85 |
| A0A0H2XGX1 | RNA methyltransferase, TrmH family | -2,85 |
| A0A0H2XG94 | Transcriptional regulator, PadR family | -2,85 |
| groES (groS) | Co-chaperonin GroES (10 kDa chaperonin) (Chaperonin-10) (Cpn10) | -2,84 |
| sraP | Serine-rich adhesin for platelets (Adhesin SraP) (Staphylococcus aureus surface protein A) | -2,83 |
| A0A0H2XGI5 | NADH-dependent flavin oxidoreductase | -2,81 |
| pheS | Phenylalanine--tRNA ligase alpha subunit (Phenylalanyl-tRNA synthetase alpha subunit) (PheRS) | -2,80 |
| A0A0H2XJW8 | Putative pyridoxal phosphate-dependent acyltransferase | -2,79 |
| rpoZ | DNA-directed RNA polymerase subunit omega (RNAP omega subunit) | -2,79 |
| rex | Redox-sensing transcriptional repressor Rex | -2,78 |
| dat | D-alanine aminotransferase | -2,78 |
| A0A0H2XJV3 | ABC transporter, ATP-binding protein | -2,77 |
| A0A0H2XFK7 | Transcriptional regulator, MarR family | -2,77 |
| A0A0H2XJX6 | Aminotransferase, class V | -2,76 |
| A0A0H2XK03 | Gluconeogenesis factor | -2,75 |
| narJ | Respiratory nitrate reductase, delta subunit | -2,75 |
| sarR | HTH-type transcriptional regulator SarR (Staphylococcal accessory regulator R) | -2,74 |
| A0A0H2XFM8 | Acetyltransferase, GNAT family | -2,74 |
| A0A0H2XGQ7 | DUF1027 domain-containing protein | -2,74 |
| Q2FIM8 | Nucleotide-binding protein SAUSA300_0748 | -2,73 |
| rsmA (ksgA) | Ribosomal RNA small subunit methyltransferase A | -2,70 |
| rpmC | Large ribosomal subunit protein uL29 | -2,69 |
| gpsB | Cell cycle protein GpsB (Guiding PBP1-shuttling protein) | -2,68 |
| A0A0H2XF94 | Initiation-control protein YabA | -2,68 |
| A0A0H2XHI0 | Uncharacterized protein | -2,67 |
| isdI | Heme oxygenase (staphylobilin-producing) 2 | -2,65 |
| vga | ABC transporter, ATP-binding protein | -2,65 |
| A0A0H2XKB6 | Cytosolic protein | -2,65 |
| A0A0H2XFK9 | Putative cobalt ABC transporter, ATP-binding protein | -2,64 |
| phoH | PhoH-like protein | -2,64 |
| A0A0H2XJG1 | Glyoxalase family protein | -2,64 |
| A0A0H2XHT6 | 5' nucleotidase family protein | -2,64 |
| cinA | Putative competence-damage inducible protein | -2,60 |
| thiD | pyridoxal kinase | -2,60 |
| UPF0297 protein SAUSA300_1574 | Uncharacterized protein | -2,60 |
| A0A0H2XET3 | Lipoprotein | -2,59 |
| A0A0H2XDT0 | Nitroreductase family protein | -2,59 |
| tadA | tRNA-specific adenosine deaminase | -2,59 |
| xerD | Tyrosine recombinase XerD | -2,59 |
| Q2FI72 | UPF0738 protein SAUSA300_0906 | -2,58 |
| lexA | LexA repressor | -2,57 |
| A0A0H2XIG4 | Putative N-acetyltransferase | -2,56 |
| vraB | Putative acetyl-CoA C-acetyltransferase VraB | -2,56 |
| A0A0H2XET4 | Transaldolase | -2,56 |
| icd | Isocitrate dehydrogenase [NADP] | -2,55 |
| trxA | Thioredoxin (Trx) | -2,54 |
| scpB | Segregation and condensation protein B | -2,54 |
| dagK | Diacylglycerol kinase (DAG kinase) (DAGK) | -2,52 |
| A0A0H2XI16 | Hydrolase, TatD family | -2,52 |
| Q2FH32 | TelA-like protein SAUSA300_1299 | -2,52 |
| A0A0H2XKF5 | YdhG-like domain-containing protein | -2,51 |
| A0A0H2XGD8 | Aldo/keto reductase family protein | -2,50 |
| hprK | HPr kinase/phosphorylase (HPrK/P) | -2,50 |
| ptsH | Phosphocarrier protein HPr (Histidine-containing protein) | -2,49 |
| A0A0H2XIL9 | AAA+ ATPase domain-containing protein | -2,49 |
| A0A0H2XIW6 | RNA-binding S4 domain-containing protein | -2,49 |
| glnR | Glutamine synthetase repressor | -2,48 |
| Q2FH10 | Bacilliredoxin SAUSA300_1321 | -2,48 |
| A0A0H2XGT6 | Carboxymuconolactone decarboxylase-like domain-containing protein | -2,47 |
| atpH | ATP synthase subunit delta (ATP synthase F(1) sector subunit delta) | -2,46 |
| A0A0H2XI85 | Chorismate mutase/phospho-2-dehydro-3-deoxyheptonate aldolase | -2,46 |
| ddh | D-lactate dehydrogenase | -2,46 |
| greA | Transcription elongation factor GreA (Transcript cleavage factor GreA) | -2,46 |
| Q2FHY3 | UPF0223 protein SAUSA300_0997 | -2,46 |
| A0A0H2XGP9 | DUF2187 domain-containing protein | -2,46 |
| A0A0H2XJR8 | 16S rRNA (Guanine(966)-N(2))-methyltransferase RsmD | -2,45 |
| gmk | Guanylate kinase (GMP kinase) | -2,45 |
| opuCc | Glycine betaine/carnitine/choline ABC transporter | -2,45 |
| A0A0H2XJD6 | Acyl-CoA thioesterase | -2,44 |
| rpoF | RNA polymerase sigma factor | -2,44 |
| A0A0H2XHY2 | FAD/NAD(P)-binding Rossmann fold Superfamily | -2,44 |
| A0A0H2XDD9 | Metallo-beta-lactamase domain-containing protein | -2,43 |
| Q2FG28 | Putative universal stress protein SAUSA300_1656 | -2,43 |
| msrA | Peptide methionine sulfoxide reductase MsrA (Protein-methionine-S-oxide reductase) | -2,43 |
| smc | Chromosome partition protein Smc | -2,42 |
| Q2FFH4 | Uncharacterized protein SAUSA300_1902 | -2,42 |
| A0A0H2XGA2 | DUF488 domain-containing protein | -2,41 |
| gcvPB | Probable glycine dehydrogenase (decarboxylating) subunit 2 | -2,40 |
| A0A0H2XI62 | dITP/XTP pyrophosphatase | -2,39 |
| A0A0H2XIC7 | Putative Na+/H+ antiporter | -2,39 |
| nusG | Transcription termination/antitermination protein NusG | -2,39 |
| fur | Ferric uptake regulation protein | -2,39 |
| rplQ | Large ribosomal subunit protein bL17 (50S ribosomal protein L17) | -2,39 |
| A0A0H2XEU1 | Acetyltransferase, GNAT family | -2,38 |
| A0A0H2XFG3 | ComE operon protein 2 | -2,37 |
| A0A0H2XG74 | Oxidoreductase, aldo/keto reductase family | -2,37 |
| A0A0H2XHK4 | TIGR04141 family sporadically distributed protein | -2,37 |
| dtd | D-aminoacyl-tRNA deacylase (DTD) (Gly-tRNA(Ala) deacylase) | -2,36 |
| Q2FIX0 | Probable transcriptional regulatory protein SAUSA300_0655 | -2,36 |
| A0A0H2XEF1 | 2-oxoisovalerate dehydrogenase, E1 component, beta subunit | -2,35 |
| A0A0H2XIG3 | Ribosomal RNA small subunit methyltransferase E | -2,34 |
| ampA | Cytosol aminopeptidase | -2,34 |
| cysS | Cysteine--tRNA ligase (Cysteinyl-tRNA synthetase) (CysRS) | -2,33 |
| A0A0H2XGR3 | Uncharacterized protein | -2,33 |
| A0A0H2XEM9 | CBS domain pair protein | -2,31 |
| A0A0H2XJ58 | Putative Pyridine nucleotide-disulphide oxidoreductase | -2,31 |
| A0A0H2XHV1 | Bacterial transcription activator effector binding domain-containing protein | -2,30 |
| lytR | Transcriptional regulatory protein LytR (Sensory transduction protein LytR) | -2,30 |
| A0A0H2XFN8 | Isochorismatase family protein | -2,29 |
| dnaN | Beta sliding clamp | -2,28 |
| pepT | Peptidase T (Aminotripeptidase) (Tripeptidase) (Tripeptide aminopeptidase) | -2,28 |
| cbf1 | Cmp-binding-factor 1 | -2,28 |
| A0A0H2XFM0 | NmrA-like domain-containing protein | -2,28 |
| prmA | Ribosomal protein L11 methyltransferase (L11 Mtase) | -2,27 |
| A0A0H2XE63 | Putative transcriptional regulator | -2,26 |
| recN | DNA repair protein RecN (Recombination protein N) | -2,26 |
| ndk | Nucleoside diphosphate kinase (NDK) (NDP kinase) (Nucleoside-2-P kinase) | -2,26 |
| A0A0H2XH60 | uroporphyrinogen-III C-methyltransferase | -2,25 |
| Q2FGG0 | Putative pyruvate, phosphate dikinase regulatory protein (PPDK regulatory protein) | -2,24 |
| A0A0H2XED8 | YwdI family protein | -2,24 |
| pheT | Phenylalanine--tRNA ligase beta subunit (Phenylalanyl-tRNA synthetase beta subunit) (PheRS) | -2,24 |
| A0A0H2XH10 | Transcriptional regulator, TetR family | -2,24 |
| msrA | peptide-methionine (S)-S-oxide reductase | -2,23 |
| A0A0H2XH88 | ABC transporter, ATP-binding protein | -2,23 |
| cvfB | Conserved virulence factor B | -2,23 |
| Q2FFY7 | Putative dipeptidase SAUSA300_1697 | -2,22 |
| lytH | Probable cell wall amidase LytH | -2,20 |
| A0A0H2XEM2 | DUF1963 domain-containing protein | -2,20 |
| Q2FDS6 | Uncharacterized hydrolase SAUSA300_2518 | -2,19 |
| trpS | Tryptophan--tRNA ligase | -2,19 |
| nusB | Transcription antitermination protein NusB (Antitermination factor NusB) | -2,18 |
| A0A0H2XJ15 | TPR domain protein | -2,18 |
| acyP | Acylphosphatase (Acylphosphate phosphohydrolase) | -2,17 |
| A0A0H2XGT1 | Luciferase-like domain-containing protein | -2,17 |
| rpsH | Small ribosomal subunit protein uS8 (30S ribosomal protein S8) | -2,17 |
| A0A0H2XG03 | Glycine cleavage H-protein | -2,16 |
| A0A0H2XDE4 | HTH-type transcriptional regulator MgrA | -2,15 |
| A0A0H2XGQ0 | YlbF family regulator | -2,15 |
| ccpA | Catabolite control protein A | -2,15 |
| A0A0H2XF07 | Putative zinc-binding dehydrogenase | -2,14 |
| A0A0H2XGS6 | TIGR01741 family protein | -2,14 |
| A0A0H2XHI3 | Inositol monophosphatase family protein | -2,14 |
| A0A0H2XED7 | DegV family protein | -2,14 |
| A0A0H2XID1 | Putative transcriptional regulator | -2,14 |
| A0A0H2XH36 | Glyoxylase family protein | -2,14 |
| ansA | L-asparaginase | -2,14 |
| nfrA | NADPH-dependent oxidoreductase | -2,12 |
| Q2FFL5 | UPF0435 protein SAUSA300_1861 | -2,11 |
| tcaR | Transcriptional regulator TcaR | -2,10 |
| A0A0H2XIK9 | Cytosolic protein | -2,10 |
| A0A0H2XGU4 | Iron-sulfur cluster carrier protein | -2,10 |
| A0A0H2XIL8 | Serine hydrolase family protein | -2,10 |
| UPF0297 protein SAUSA300_1574 | Uncharacterized protein | -2,08 |
| A0A0H2XG61 | thiamine diphosphokinase | -2,08 |
| A0A0H2XI33 | 5' nucleotidase family protein | -2,08 |
| A0A0H2XIX0 | Uncharacterized protein | -2,07 |
| rpoA | DNA-directed RNA polymerase subunit alpha (RNAP subunit alpha) (RNA polymerase subunit alpha) (Transcriptase subunit alpha) | -2,07 |
| lpdA | Dihydrolipoyl dehydrogenase | -2,06 |
| A0A0H2XDE9 | Acetyltransferase, GNAT family | -2,06 |
| groEL (groL) | Chaperonin GroEL (60 kDa chaperonin) (Chaperonin-60) (Cpn60) | -2,05 |
| pepF | Oligoendopeptidase F | -2,04 |
| lpdA | Dihydrolipoyl dehydrogenase | -2,04 |
| A0A0H2XHH8 | Pyridoxal phosphate homeostasis protein (PLP homeostasis protein) | -2,03 |
| A0A0H2XFU2 | Sce7725 family protein | -2,03 |
| pbpA | Penicillin-binding protein 1 | -2,03 |
| A0A0H2XJ65 | Putative 5'(3')-deoxyribonucleotidase | -2,03 |
| accC | Acetyl-CoA carboxylase, biotin carboxylase | -2,03 |
| A0A0H2XG09 | Transcriptional regulator, GntR family | -2,03 |
| A0A0H2XFW2 | UspA domain-containing protein | -2,02 |
| arsR | Transcriptional repressor, ArsR family | -2,02 |
| A0A0H2XI27 | Hydrolase, haloacid dehalogenase-like family | -2,02 |
| A0A0H2XEM8 | Uncharacterized protein | -2,01 |
| tyrS | Tyrosine--tRNA ligase (Tyrosyl-tRNA synthetase) (TyrRS) | -2,01 |
| Q2FIJ2 | Organic hydroperoxide resistance protein-like | -2,01 |
| spoVG | Putative septation protein SpoVG | -2,01 |
| scpA | Segregation and condensation protein A | -2,01 |

| **Table S5. DEPs proteins detected from the comparison between TCH1516 at early moxifloxacin treatment (5-90 min) compared to persister state (240-330 min). NA= non assigned pathway.** | | | |
| --- | --- | --- | --- |
| **Gene Names** | **Pathway** | **Protein description** | **log2 Fold Change (FC)** |
| A0A0H2XGX3 | NA | Putative phage-related DNA recombination protein | 10.44 |
| A0A0H2XJ00 | NA | Conserved hypothetical phage protein | 9.50 |
| A0A0H2XI78 | NA | PhiPVL ORF41-like protein | 8.62 |
| A0A0H2XGG0 | NA | PhiPVL ORF39-like protein | 8.44 |
| A0A0H2XFK3 | NA | Phi77 ORF014-like protein, phage anti-repressor protein | 8.43 |
| A0A0H2XIZ5 | NA | Putative phage transcriptional regulator | 8.14 |
| A0A0H2XH41 | NA | Pathogenicity island protein | 8.07 |
| A0A0H2XIJ7 | NA | Conserved hypothetical phage protein | 7.79 |
| A0A0H2XDW8 | NA | Pathogenicity island protein | 7.78 |
| rpsR | NA | Small ribosomal subunit protein bS18 | 7.69 |
| dut | NA | dUTP diphosphatase | 7.37 |
| rpmI | NA | Large ribosomal subunit protein bL35 | 6.63 |
| rpmF | NA | Large ribosomal subunit protein bL32 | 6.41 |
| A0A0H2XJ35 | NA | Staphylococcal protein | 6.06 |
| rpsZ rpsN1 | NA | Small ribosomal subunit protein uS14B | 5.96 |
| A0A0H2XFG8 | NA | PhiPVL ORF050-like protein | 5.90 |
| rplT | NA | Large ribosomal subunit protein bL20 | 5.86 |
| coa | NA | Staphylocoagulase | 5.77 |
| A0A0H2XJY5 | NA | Single-stranded DNA-binding protein (SSB) | 5.67 |
| A0A0H2XIZ8 | NA | Putative DNA primase | 5.66 |
| accB | Lipid metabolism | Biotin carboxyl carrier protein of acetyl-CoA carboxylase | 5.32 |
| rpmB | NA | Large ribosomal subunit protein bL28 | 5.20 |
| A0A0H2XGJ9 | NA | Putative transcriptional regulator | 5.08 |
| rplR | NA | Large ribosomal subunit protein uL18 | 4.95 |
| rplB | NA | Large ribosomal subunit protein uL2 | 4.92 |
| A0A0H2XKN2 | NA | Uncharacterized protein | 4.82 |
| A0A0H2XFT2 | NA | UPF0154 protein SAUSA300_1240 | 4.72 |
| pyc | NA | Pyruvate carboxylase | 4.71 |
| A0A0H2XK54 | NA | Pathogenicity island protein | 4.52 |
| A0A0H2XGY6 | NA | Pathogenicity island protein | 4.39 |
| A0A0H2XFV0 | NA | Isoprenylcysteine carboxyl methyltransferase | 4.31 |
| nuc | NA | Thermonuclease | 4.28 |
| A0A0H2XGA4 | NA | DUF4930 family protein | 4.26 |
| A0A0H2XIX8 | NA | Putative lipoprotein | 4.18 |
| rpsL | NA | Small ribosomal subunit protein uS12 | 4.17 |
| rpmJ | NA | Large ribosomal subunit protein bL36 | 4.17 |
| A0A0H2XFC0 | NA | ABC transporter, ATP-binding protein, MsbA family | 3.99 |
| oppB | NA | Oligopeptide ABC transporter, permease protein | 3.92 |
| rplW | NA | Large ribosomal subunit protein uL23 | 3.92 |
| A0A0H2XGZ3 | NA | Formate/nitrite transporter family protein | 3.88 |
| A0A0H2XI02 | NA | 5'-nucleotidase family protein | 3.87 |
| rplP | NA | Large ribosomal subunit protein uL16 | 3.76 |
| clfB | NA | Clumping factor B | 3.75 |
| A0A0H2XKL2 | NA | YolD-like family protein | 3.66 |
| leuD | L-leucine biosynthesis | 3-isopropylmalate dehydratase small subunit | 3.65 |
| A0A0H2XHQ0 | NA | Putative transcriptional regulator | 3.61 |
| A0A0H2XFB1 | NA | Uncharacterized protein | 3.56 |
| rpmA | NA | Large ribosomal subunit protein bL27 | 3.53 |
| nrdI | NA | Protein NrdI | 3.53 |
| rplO | NA | Large ribosomal subunit protein uL15 | 3.50 |
| A0A0H2XFD4 | NA | DUF4889 domain-containing protein | 3.50 |
| rpsS | NA | Small ribosomal subunit protein uS19 | 3.48 |
| A0A0H2XH45 | NA | Putative lipoprotein | 3.48 |
| A0A0H2XHI4 | NA | Phage protein | 3.47 |
| rpsU | NA | Small ribosomal subunit protein bS21 | 3.46 |
| topB | NA | DNA topoisomerase 3 | 3.46 |
| A0A0H2XIB2 | NA | Uncharacterized protein | 3.46 |
| A0A0H2XH49 | NA | Phi77 ORF026-like protein | 3.44 |
| rplS | NA | Large ribosomal subunit protein bL19 | 3.44 |
| A0A0H2XH00 | NA | Fibrinogen-binding protein | 3.41 |
| A0A0H2XHX8 | NA | Putative membrane protein | 3.36 |
| A0A0H2XEU8 | NA | Permease | 3.34 |
| A0A0H2XJ51 | NA | Phage capsid protein | 3.34 |
| A0A0H2XHA6 | NA | DUF4176 domain-containing protein | 3.28 |
| rpsQ | NA | Small ribosomal subunit protein uS17 | 3.28 |
| A0A0H2XIP5 | NA | Uncharacterized protein | 3.28 |
| A0A0H2XH31 | NA | Amino acid ABC transporter, permease/substrate-binding protein | 3.28 |
| rplC | NA | Large ribosomal subunit protein uL3 | 3.27 |
| A0A0H2XKJ6 | NA | DUF805 domain-containing protein | 3.27 |
| A0A0H2XHE4 | NA | Cell wall-active antibiotics response LiaF-like | 3.25 |
| rplD | NA | Large ribosomal subunit protein uL4 | 3.25 |
| A0A0H2XJA4 | NA | Cytochrome D ubiquinol oxidase, subunit II | 3.24 |
| fnbB | NA | Fibronectin-binding protein B | 3.20 |
| uppP | NA | Undecaprenyl-diphosphatase | 3.18 |
| A0A0H2XJL8 | NA | Polysaccharide biosynthesis protein | 3.17 |
| A0A0H2XE56 | NA | DUF2188 domain-containing protein | 3.17 |
| A0A0H2XEB8 | NA | Pathogenicity island protein | 3.17 |
| A0A0H2XEB7 | NA | Integral membrane protein | 3.17 |
| A0A0H2XES4 | NA | Ribonucleoside-diphosphate reductase | 3.15 |
| A0A0H2XF08 | NA | Uncharacterized protein | 3.13 |
| A0A0H2XF42 | NA | Cytochrome D ubiquinol oxidase, subunit I | 3.13 |
| nupC | NA | Pyrimidine nucleoside transport protein | 3.13 |
| smpB | NA | SsrA-binding protein (Small protein B) | 3.13 |
| A0A0H2XIC5 | NA | Acetyltransferase, GNAT family | 3.12 |
| A0A0H2XF33 | NA | ImpB/MucB/SamB family protein | 3.11 |
| srrB | NA | Sensor protein SrrB | 3.10 |
| infC | NA | Translation initiation factor IF-3 | 3.10 |
| lctP | NA | L-lactate permease | 3.06 |
| yidC | NA | Membrane protein insertase YidC (Foldase YidC) | 3.05 |
| A0A0H2XGY2 | NA | Abi family protein | 3.05 |
| rpsE | NA | Small ribosomal subunit protein uS5 | 3.03 |
| A0A0H2XHI5 | NA | Uncharacterized protein | 3.02 |
| A0A0H2XJ93 | NA | Alpha/beta hydrolase | 2.99 |
| lytS | NA | Sensor histidine kinase/phosphatase LytS | 2.98 |
| A0A0H2XH55 | NA | Cold shock protein CspA | 2.97 |
| A0A0H2XHT5 | NA | YggT family protein | 2.97 |
| A0A0H2XJU8 | NA | Lytic regulatory protein | 2.96 |
| sbi | NA | Immunoglobulin-binding protein Sbi | 2.96 |
| A0A0H2XDI6 | NA | Putative restriction/modification system specificity protein | 2.95 |
| rpsG | NA | Small ribosomal subunit protein uS7 | 2.95 |
| A0A0H2XJ27 | NA | Ferredoxin | 2.94 |
| Q2FHW8 | NA | UPF0358 protein SAUSA300_1012 | 2.94 |
| rpsT | NA | Small ribosomal subunit protein bS20 | 2.93 |
| A0A0H2XG53 | NA | Uncharacterized protein | 2.89 |
| A0A0H2XES0 | NA | Putative nucleoside transporter | 2.88 |
| comK | NA | Competence transcription factor | 2.86 |
| rpsK | NA | Small ribosomal subunit protein uS11 | 2.85 |
| acpP | Lipid metabolism | Acyl carrier protein (ACP) | 2.85 |
| A0A0H2XHP7 | NA | Staphylococcal complement inhibitor | 2.85 |
| rpmD | NA | Large ribosomal subunit protein uL30 | 2.84 |
| gltT | NA | Proton/sodium-glutamate symport protein | 2.83 |
| A0A0H2XJ17 | NA | Amino acid permease | 2.82 |
| A0A0H2XIR9 | NA | Uncharacterized protein | 2.81 |
| A0A0H2XFZ5 | NA | Putative membrane protein | 2.80 |
| A0A0H2XI20 | NA | DUF2273 domain-containing protein | 2.80 |
| rplU | NA | Large ribosomal subunit protein bL21 | 2.80 |
| A0A0H2XK84 | NA | DUF2871 domain-containing protein | 2.80 |
| A0A0H2XG02 | NA | Uncharacterized protein | 2.79 |
| A0A0H2XFZ7 | NA | Putative ABC transporter protein EcsB | 2.76 |
| A0A0H2XHE1 | NA | Peptidase, rhomboid family | 2.76 |
| A0A0H2XJD2 | NA | Zinc metalloprotease | 2.76 |
| A0A0H2XEX3 | NA | DUF697 domain-containing protein | 2.73 |
| A0A0H2XJC8 | NA | Endoribonuclease MazF (Toxin MazF) | 2.71 |
| Q2FFP9 | NA | UPF0754 membrane protein SAUSA300_1796 | 2.71 |
| uvrC | NA | UvrABC system protein C (Protein UvrC) | 2.69 |
| rpsM | NA | Small ribosomal subunit protein uS13 | 2.65 |
| rplE | NA | Large ribosomal subunit protein uL5 | 2.64 |
| ldh1 | Fermentation | L-lactate dehydrogenase 1 | 2.60 |
| cshA | NA | DEAD-box ATP-dependent RNA helicase CshA | 2.59 |
| ubiE | NA | Menaquinone biosynthesis methyltransferase ubiE | 2.58 |
| argG | L-arginine biosynthesis | Argininosuccinate synthase | 2.58 |
| A0A0H2XII5 | NA | Phosphotransferase system, fructose-specific | 2.57 |
| A0A0H2XFG6 | NA | YtxH domain-containing protein | 2.56 |
| A0A0H2XGM6 | NA | ABC transporter, ATP-binding protein | 2.56 |
| rimI | NA | [Ribosomal protein bS18]-alanine N-acetyltransferase | 2.55 |
| A0A0H2XIN9 | NA | Amino acid carrier protein | 2.54 |
| ispE | NA | Putative 4-diphosphocytidyl-2-C-methyl-D-erythritol kinase | 2.53 |
| rpsD | NA | Small ribosomal subunit protein uS4 | 2.53 |
| A0A0H2XGA6 | NA | L-lactate permease | 2.53 |
| Q2FFI4 | NA | UPF0316 protein SAUSA300_1892 | 2.52 |
| rpsF | NA | Small ribosomal subunit protein bS6 | 2.52 |
| plsY | Lipid metabolism | Glycerol-3-phosphate acyltransferase | 2.51 |
| nagE | NA | Phosphotransferase system, N-acetylglucosamine-specific IIBC component | 2.50 |
| sdrH | NA | Serine-aspartate repeat family protein, SdrH | 2.46 |
| A0A0H2XI26 | NA | ribonucleoside-diphosphate reductase | 2.46 |
| corA | NA | Magnesium transport protein CorA | 2.46 |
| A0A0H2XG38 | NA | Phage protein | 2.45 |
| A0A0H2XFR8 | NA | Putative exonuclease | 2.45 |
| egtUBC | NA | Probable ergothioneine transporter EgtUBC | 2.44 |
| A0A0H2XFS3 | NA | Putative lipoprotein | 2.44 |
| A0A0H2XE91 | NA | DUF3139 domain-containing protein | 2.43 |
| secF secD | NA | Multifunctional fusion protein | 2.43 |
| A0A0H2XJ47 | NA | Veg protein | 2.40 |
| glcB | NA | PTS system glucoside-specific EIICBA component | 2.39 |
| A0A0H2XJ78 | NA | Replication protein | 2.39 |
| rpsI | NA | Small ribosomal subunit protein uS9 | 2.39 |
| rpsJ | NA | Small ribosomal subunit protein uS10 | 2.37 |
| A0A0H2XII8 | NA | Cold shock protein CspA | 2.37 |
| Q2FJ50 | NA | UPF0741 protein SAUSA300_0575 | 2.36 |
| fmt | NA | Fmt protein | 2.35 |
| A0A0H2XE83 | NA | protein-N(pi)-phosphohistidine--sucrose phosphotransferase | 2.35 |
| nnrD | NA | ADP-dependent (S)-NAD(P)H-hydrate dehydratase | 2.34 |
| psuG | NA | Pseudouridine-5'-phosphate glycosidase | 2.31 |
| A0A0H2XI10 | NA | Glycosyl transferase, group 1 family protein | 2.31 |
| topA | NA | DNA topoisomerase 1 | 2.30 |
| cshB | NA | DEAD-box ATP-dependent RNA helicase CshB | 2.29 |
| glpP | NA | Glycerol uptake operon antiterminator regulatory protein | 2.28 |
| A0A0H2XHY6 | NA | Urea amidolyase-related protein | 2.26 |
| rplL | NA | Large ribosomal subunit protein bL12 | 2.26 |
| ung | NA | Uracil-DNA glycosylase (UDG) | 2.25 |
| rpsC | NA | Small ribosomal subunit protein uS3 | 2.24 |
| thrS | NA | Threonine--tRNA ligase | 2.24 |
| ptsG glcA | NA | PTS system glucose-specific EIICBA component | 2.24 |
| A0A0H2XIU2 | NA | Monooxygenase family protein | 2.24 |
| rplK | NA | Large ribosomal subunit protein uL11 | 2.23 |
| A0A0H2XI92 | NA | 1-acyl-sn-glycerol-3-phosphate acyltransferases | 2.23 |
| menA | Quinol/quinone metabolism | 1,4-dihydroxy-2-naphthoate octaprenyltransferase | 2.22 |
| potD | NA | Spermidine/putrescine ABC transporter | 2.21 |
| prsA | NA | Foldase protein PrsA | 2.20 |
| A0A0H2XK72 | NA | Ribosome biogenesis GTPase A | 2.20 |
| rplM | NA | Large ribosomal subunit protein uL13 | 2.19 |
| rpsH | NA | Small ribosomal subunit protein uS8 | 2.19 |
| A0A0H2XGF4 | NA | L-cystine uptake protein TcyP | 2.17 |
| A0A0H2XDY4 | NA | Uncharacterized protein | 2.17 |
| rlmN | NA | Probable dual-specificity RNA methyltransferase RlmN | 2.16 |
| vraS | NA | Sensor protein VraS | 2.16 |
| A0A0H2XGK7 | NA | PTS system, IIBC components | 2.15 |
| nfo | NA | Probable endonuclease 4 | 2.13 |
| glvC | NA | PTS system, arbutin-like IIBC component | 2.13 |
| A0A0H2XIM3 | NA | Uncharacterized protein | 2.13 |
| saeS | NA | Histidine protein kinase SaeS | 2.11 |
| A0A0H2XDX7 | NA | Putative membrane protein | 2.10 |
| nuc | NA | Thermonuclease | 2.10 |
| qoxB | Energy metabolism | Probable quinol oxidase subunit 1 | 2.10 |
| A0A0H2XDN2 | NA | Oxidoreductase, Gfo/Idh/MocA family | 2.09 |
| rpoY | NA | DNA-directed RNA polymerase subunit epsilon | 2.09 |
| serS | Aminoacyl-tRNA biosynthesis | Serine--tRNA ligase | 2.09 |
| A0A0H2XES5 | NA | Transcriptional regulator, MarR family | 2.08 |
| dps | NA | General stress protein 20U | 2.08 |
| A0A0H2XJG9 | NA | Maebl | 2.08 |
| A0A0H2XG55 | NA | UPF0291 protein SAUSA300_1238 | 2.08 |
| A0A0H2XEE7 | NA | nitrate reductase (quinone) | 2.05 |
| A0A0H2XG58 | NA | Putative lipoprotein | 2.03 |
| thiI | Cofactor biosynthesis | Probable tRNA sulfurtransferase | 2.03 |
| pgsA | Lipid metabolism | CDP-diacylglycerol--glycerol-3-phosphate 3-phosphatidyltransferase | 2.03 |
| rpoB | NA | DNA-directed RNA polymerase subunit beta | 2.03 |
| A0A0H2XGW2 | NA | Transcription regulatory protein | 2.03 |
| A0A0H2XIC9 | NA | Putative teichoic acid biosynthesis protein | 2.01 |
| qoxA | NA | Probable quinol oxidase subunit 2 | 2.01 |
| xseB | NA | Exodeoxyribonuclease 7 small subunit | 2.00 |
| sodA | NA | Superoxide dismutase [Mn/Fe] 1 | -2.01 |
| Q2FDY2 | NA | Putative NAD(P)H nitroreductase SAUSA300_2462 | -2.04 |
| prmA | NA | Ribosomal protein L11 methyltransferase | -2.05 |
| mtlA | NA | Mannitol-specific phosphotransferase enzyme | -2.05 |
| A0A0H2XDE9 | NA | Acetyltransferase, GNAT family | -2.07 |
| A0A0H2XFU8 | NA | Putative homoserine O-acetyltransferase | -2.11 |
| A0A0H2XH22 | NA | Putative glycerophosphoryl diester phosphodiesterase | -2.15 |
| clpC | NA | ATP-dependent Clp protease ATP-binding subunit ClpC | -2.20 |
| A0A0H2XJQ4 | NA | Acetyltransferase, GNAT family | -2.24 |
| purC | Purine metabolism | Phosphoribosylaminoimidazole-succinocarboxamide synthase | -2.30 |
| A0A0H2XH33 | NA | PhnB-like domain-containing protein | -2.31 |
| A0A0H2XGI5 | NA | NADH-dependent flavin oxidoreductase | -2.34 |
| A0A0H2XGT0 | NA | SIS domain protein | -2.36 |
| A0A0H2XH30 | NA | Peptidase, M20/M25/M40 family | -2.38 |
| folD | One-carbon metabolism | Bifunctional protein FolD | -2.40 |
| groEL groL | NA | Chaperonin GroEL | -2.43 |
| A0A0H2XIZ0 | NA | OsmC/Ohr family protein | -2.48 |
| Q2FE21 | NA | Uncharacterized oxidoreductase SAUSA300_2422 | -2.55 |
| A0A0H2XE07 | NA | Phage protein | -2.69 |
| A0A0H2XEU5 | NA | Excinuclease ABC subunit B | -2.80 |
| mcsB | NA | Protein-arginine kinase | -2.93 |
| A0A0H2XFV1 | NA | HAD-superfamily hydrolase, subfamily IA, variant 1 | -2.95 |
| A0A0H2XFB9 | NA | DNA-directed RNA polymerase subunit delta | -3.01 |
| A0A0H2XF54 | NA | Bacterial luciferase family protein | -3.03 |
| hrcA | NA | Heat-inducible transcription repressor HrcA | -3.08 |
| ctsR | NA | Transcriptional regulator CtsR | -3.65 |
| purS | Purine metabolism | Phosphoribosylformylglycinamidine synthase | -3.92 |
| groES groS | NA | Co-chaperonin GroES | -3.99 |
| A0A0H2XKB6 | NA | Cytosolic protein | -4.71 |
| A0A0H2XHK4 | NA | TIGR04141 family sporadically distributed protein | -6.63 |

**Additional References**

1. Jensen, C., et al., *Staphylococcus aureus ClpX localizes at the division septum and impacts transcription of genes involved in cell division, T7-secretion, and SaPI5-excision.* Sci Rep, 2019. **9**(1): p. 16456.

2. Stahlhut, S.G., et al., *The ClpXP protease is dispensable for degradation of unfolded proteins in Staphylococcus aureus.* Sci Rep, 2017. **7**(1): p. 11739.

3. Boyle-Vavra, S., et al., *VraT/YvqF is required for methicillin resistance and activation of the VraSR regulon in Staphylococcus aureus.* Antimicrob Agents Chemother, 2013. **57**(1): p. 83-95.

4. Abdelmalek, N., et al., *The Secondary Resistome of Methicillin-Resistant Staphylococcus aureus to β-Lactam Antibiotics.* Antibiotics (Basel), 2025. **14**(2).

5. Suzuki, S., et al., *Structure of N-formylglycinamide ribonucleotide amidotransferase II (PurL) from Thermus thermophilus HB8.* Acta Crystallogr Sect F Struct Biol Cryst Commun, 2012. **68**(Pt 1): p. 14-9.

6. Anand, R., et al., *A model for the Bacillus subtilis formylglycinamide ribonucleotide amidotransferase multiprotein complex.* Biochemistry, 2004. **43**(32): p. 10343-52.
